# Actaticas A−G, Cycloartane Triterpenes From *Actaea asiatica* With Their Antiproliferative Activity

**DOI:** 10.3389/fchem.2021.695456

**Published:** 2021-07-29

**Authors:** Meigeng Hu, Dan Zhao, Xudong Xu, Guoxu Ma, Haifeng Wu, Xi Chen

**Affiliations:** ^1^ Key Laboratory of Bioactive Substances and Resource Utilization of Chinese Herbal Medicine, Ministry of Education, Beijing Key Laboratory of Innovative Drug Discovery of Traditional Chinese Medicine (Natural Medicine) and Translational Medicine, Institute of Medicinal Plant Development, Peking Union Medical College and Chinese Academy of Medical Sciences, Beijing, China; ^2^ Beijing Key Laboratory of Innovative Drug Discovery of Traditional Chinese Medicine (Natural Medicine) and Translational Medicine, Institute of Medicinal Plant Development, Peking Union Medical College and Chinese Academy of Medical Sciences, Beijing, China; ^3^ Key Laboratory of Efficacy Evaluation of Chinese Medicine Against Glycolipid Metabolic Disorders, State Administration of Traditional Chinese Medicine, Institute of Medicinal Plant Development, Peking Union Medical College and Chinese Academy of Medical Sciences, Beijing, China

**Keywords:** Actaea asiatica, cycloartane triterpenes, antiproliferative activity, HT-29 cell lines, MCF-7 cell lines

## Abstract

Phytochemical studies on the rhizomes of *Actaea asiatica* led to the isolation of seven new cycloartane triterpenes, actaticas A−G (**1−7**). Their structures were determined by NMR, HRESIMS, and chemical analysis. All the isolates were evaluated for their antiproliferative activity against HT-29 and McF-7 cell lines. The results showed that all the compounds displayed cytotoxicity. All compounds showed significant inhibitory effects with IC_50_ values of 9.2–26.4 μM.

## Introduction

**FIGURE 1:**
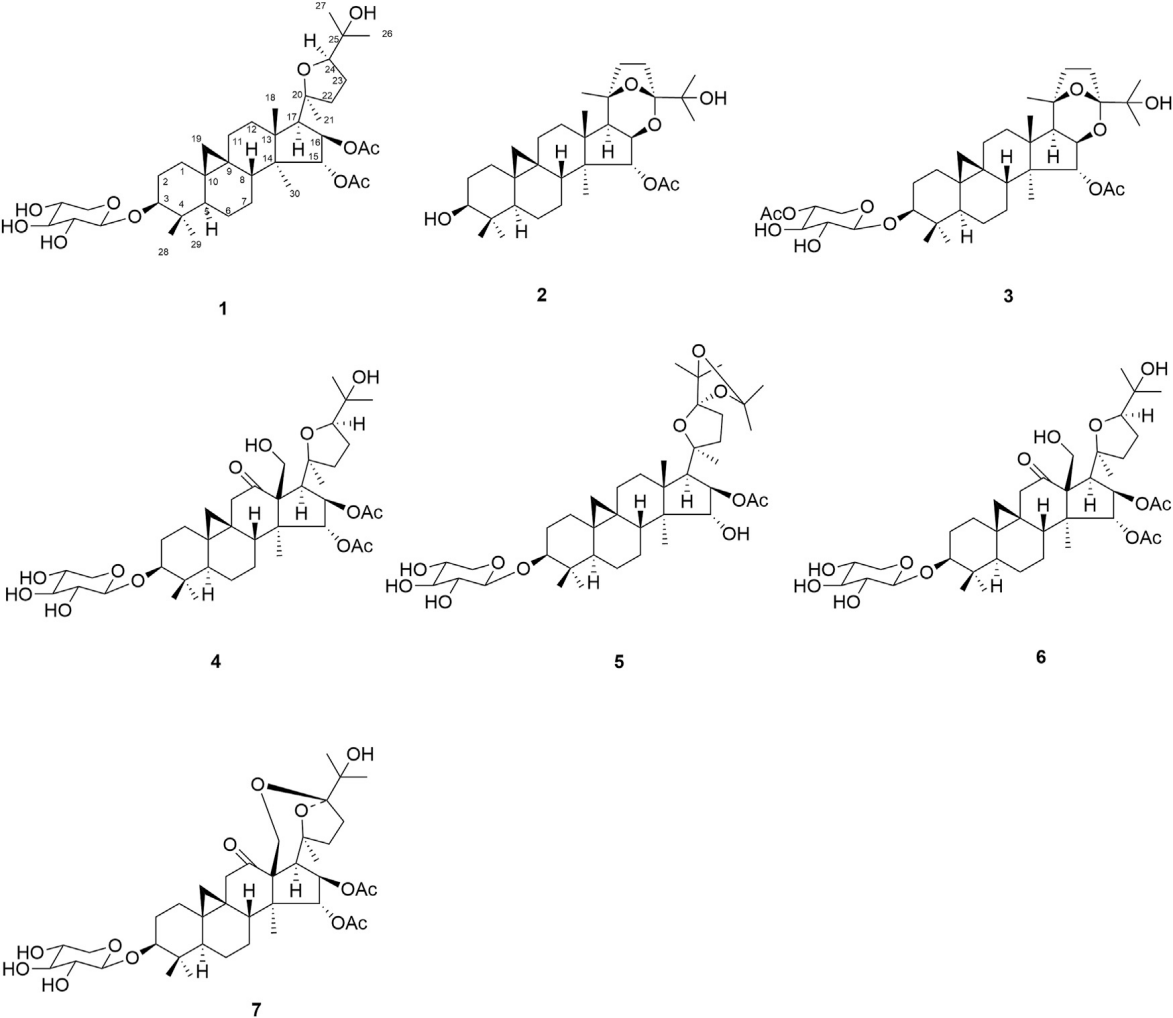
Structures of compounds **1–7**.

*Actaea asiatica* H. Hara, a perennial herb belonging to the family Ranunculaceae, is mainly distributed in the southwest and northwest of China. Its roots have been traditionally used among the Tujia folk in Hubei Province for treating headache, sore throat, rheumatic pain, rubella, measles, pertussis, uterine prolapse, and dog bites (Gao et al., 2006a; Gao et al., 2006b; Fan et al., 2007; Gao et al., 2007). Phytochemical studies indicated that the genus *Actaea* contained cycloartane triterpene glycosides with cytotoxic activities (Kusano et al., 1998; Kusano et al., 1999; Gao et al., 2006b). However, little systematic chemical work on *A. asiatica* has been carried out so far. In order to find the bioactive constituents from *A. asiatica*, chemical research were carried out, resulting in the isolation of seven new cycloartane triterpene glycosides, namely, actaticas A–G (**1–7**) (Figure 1). Their structures were determined by spectroscopic analysis and chemical methods. Herein, structural elucidation of compounds **1–7** was reported as well as their cytotoxic activities.

## Materials and Methods

### General Experimental Procedures

Optical rotations were obtained on a PerkinElmer 341 digital polarimeter. IR spectra were recorded on Shimadzu FTIR-8400S spectrometers. NMR spectra were obtained with a Bruker AV III 600 NMR spectrometer (chemical shift values are presented as *δ* values with TMS as the internal standard). HR-ESIMS spectra were performed on a LTQ-Obitrap XL spectrometer. Preparative HPLC was performed on a Lumtech K-1001 analytic LC equipped with two pumps of K-501, a UV detector of K-2600, and an YMC Pack C_18_ column (250 × 10 mm, i.d., 5 μm, YMC Co. Ltd., Japan) eluted with CH_3_OH-H_2_O at a flow rate of 2 ml/min. C_18_ reversed–phase silica gel (40–63 μm, Merk, Darmstadt, Germany), MCI gel (CHP 20P, 75–150 μm, Mitsubishi Chemical Corporation, Tokyo, Japan), and silica gel (100–200 mesh, Qingdao Marine Chemical plant, Qingdao, the People’s Republic of China) were used for column chromatography. Pre-coated silica gel GF254 plates (Zhi Fu Huang Wu Pilot Plant of Silica Gel Development, Yantai, the People’s Republic of China) were used for TLC. All solvents used were of analytical grade (Beijing Chemical Works).

### Plant Material

The plants of *A. asiatica* were collected at Jinfuo Mountain in Chongqing province, the People’s Republic of China, in November 2016, and were authenticated by Professor Sirong Yi. The voucher specimen (CS161108) has been deposited at the Institute of Medicinal Plant Development, Chinese Academy of Medical Sciences.

#### Extraction and Isolation

The air-dried powdered rhizomes *A. asiatica* (6.8 kg) was extracted with 95% EtOH (20 L) three times (each time for 2 h). Removal of the EtOH under reduced pressure yielded the extract (879 g). The residue was suspended in H_2_O (1.5 L) and partitioned with petroleum ether (3 × 1 L), EtOAc (3 × 1 L), acetone (3 × 1 L), and *n*-BuOH (3 × 1 L) successively. The EtOAc fraction (510 g) was subjected to CC over silica gel (100–200 mesh, 8 × 100 cm) eluting with a stepwise gradient of CH_2_Cl_2_-MeOH (from 1:0 to 0:1) to afford six fractions A–F. Fraction B (29.4 g) was subjected to MCI column chromatography (4 × 80 cm) elution with MeOH-H_2_O (40:60; 60:40; 70:30; 80:20; 100:0, v/v) giving five subfractions (Fr. B1–B5). Subfraction B3 (911 mg) was chromatographed by semi-preparative HPLC using acetonitrile–H_2_O (75: 25, v/v) to yield compound 1 (9.4 mg, *t*_R_ = 26.3 min) and **7** (7.2 mg, *t*_R_ = 29.5 min). Subfraction B4 (503 mg) was purified through preparative HPLC elution using an acetonitrile–H_2_O (65: 35, v/v) system to give compound 2 (12.1 mg, *t*_R_ = 23.0 min). Fraction D (5.8 g) was loaded on an ODS C_18_ column (2 × 80 cm) eluted with MeOH-H_2_O (40:60; 60:40; 70:30; 80:20; 100:0, v/v) to give five subfractions (Fr. D1–D5). Subfraction D3 (503 mg) was chromatographed by semi-preparative HPLC using acetonitrile–H_2_O (70: 30, v/v) to yield compounds 3 (6.1 mg, *t*_R_ = 18.5 min), 4 (8.7 mg, *t*_R_ = 21.4 min), and 5 (7.0 mg, *t*_R_ = 28.3 min). Fraction F (6.7 g) was fractioned on an MCI-gel column chromatography eluted with MeOH–H_2_O (40:60; 60:40; 70:30; 80:20; 100:0, v/v) to give five subfractions (Fr. F1–F5). Subfraction F3 (223 mg) was chromatographed by preparative HPLC using acetonitrile–H_2_O (75: 25, v/v) to yield compounds 6 (5.8 mg, *t*_R_ = 22.7 min).

**Actatica A (1)**: C_39_H_62_O_11_, white amorphous powder; [*α*]20 D + 21.6 (*c* = 0.18, MeOH); IR (KBr) *ν*_max_: 3,440, 3,397, 2,924, 1,733, 1,457, 1,044 cm^−1^; UV (MeOH) *λ*_max_ (log *ε*): 200 nm; for ^1^H NMR (600 MHz, pyridine-*d*_5_) and ^13^C-APT (150 MHz, pyridine-*d*_5_) spectroscopic data, see Tables 1, 2; HR-ESIMS *m*/*z*: 729.4233 (calcd for C_39_H_62_O_11_Na [M + Na]^+^, 729.4184).

**TABLE 1:** ^1^H NMR Spectroscopic Data (600 MHz, in pyridine-*d*_5_) for compounds **1**–**7**.

No	Compounds						
	1	2	3	4	5	6	7
1	1.09 m; 1.52 m	1.22 m; 1.58 m	1.20 m; 1.58 m	1.10 m; 1.35 m	1.01 m; 1.52 m	1.06 m; 1.49 m	1.24 m; 1.37 m
2	1.92 m; 2.34 m	1.83 m; 2.40 m	1.96 m; 2.25 m	1.90 m; 2.35 m	1.92 m; 2.34 m	1.90 m; 2.34 m	1.12 m; 2.26 m
3	3.47dd (12.0, 3.6)	3.52 dd (12.0, 4.2)	3.36 dd (11.4, 3.0)	3.47 dd (12.0, 3.6)	3.49 dd (12.0, 3.6)	3.48 dd (12.0, 3.6)	3.58 dd (12.0, 3.6)
5	1.27 m	1.29, m	1.28, m	1.20 m	1.27 m	1.24, m	1.28 m
6	0.59 q (12.0);	0.71 q (6.6)	0.72 m; 1.41 m	0.52 q (12.0); 1.38 m	0.57 q (12.0);	0.63 q (12.0);	0.84 m; 1.42 m
	1.38 m				1.33 m	1.38 m	
7	1.06 m; 1.31 m	1.09 m; 2.09 m	2.02 m; 1.08 m	1.10 m; 1.29 m	1.05 m; 1.29, m	1.06 m; 1.38, m	1.21 m; 1.39 m
8	1.73, m	1.77 dd (4.8)	1.72, m	1.63 m	1.72, m	1.77 m	1.78 m
11	2.03 m; 1.10, m	2.02 m; 1.21 m	2.00 m; 1.08 m	2.04 d (10.8);	2.02 m; 1.10m	2.43 d (10.8);	2.25 d (10.8);
				1.98 d (10.8)		1.99 d (10.8)	1.92 d (10.8)
12	2.37 m; 1.76, m	1.55 m; 2.30 m	2.32 m; 1.71 m	—	2.36 m; 1.76 m	—	—
15	5.49 d (4.8)	5.70 d (3.0)	5.67 d (3.0)	5.88 d (4.8)	5.54 d (4.8)	1.47 m; 2.50 d (8.4)	5.50 d (4.8)
16	5.82 dd (4.8, 1.2)	4.47 q (3.0)	4.30 dd (8.4, 3.0)	5.90 dd (9.6, 4.8)	5.95 dd (9.6, 4.8)	5.47 t (8.4)	5.78 dd (9.6, 4.8)
17	2.76, s	2.00, m	2.00 m	2.01 m	2.80 d (10.2)	2.92 d (9.6)	3.02 t (8.4)
18	3.29, s	1.72, m	1.89, s	3.52 d (12.0);	1.49 s	3.51 d (11.4);	3.54 d (11.4);
				4.18 d (12.0)		4.03 d (11.4)	4.13 d (11.4)
19	0.18 d (4.2);	0.53, d (4.2);	0.21 d (4.2);	0.18 d (4.2);	0.26 d (4.2);	0.12 d (4.2);	0.28 d (4.2);
	0.47 d (4.2)	0.32 d (4.2)	0.42 d(4.2)	0.39 d (4.2)	0.51 d (4.2)	0.44 d (4.2)	0.41 d (4.2)
21	1.47 s	1.61 s	1.58 s	1.30 s	1.74 s	1.30 s	1.32 s
22	2.37 m; 1.76 m	2.02 m; 1.78 dd (4.8)	2.03 m; 1.80 m	2.36 m; 1.77 m	2.33 m; 1.76 m	2.39 m; 1.77 m	2.24 m; 1.63 m
23	2.29 m; 1.90, m	2.02 m; 1.57 m	2.02 m; 1.73 m	2.18 m; 1.90 m	2.29 m; 1.90 m	2.18 m; 1.90 m	2.12 m; 1.92 m
24	3.83 t (7.2)	—	—	3.72 t (7.2)	—	3.72, t (7.2)	—
26	1.32 s	1.66 s	1.66 s	1.15 s	1.33 s	1.23 s	1.24 s
26′	—	—	—	—	1.31 s	—	—
27	1.36 s	1.49, s	1.48 s	1.55 s	1.42 s	1.53 s	1.51 s
27′	—	—	—	—	1.45 s	—	—
28	1.35 s	1.40, s	1.38 s	1.55 s	1.17 s	1.55 s	1.52 s
29	1.02 s	1.10, s	1.07 s	1.00 s	1.04 s	1.02 s	1.01 s
30	1.09, s	1.23, s	1.12 s	1.31 s	1.25 s	1.22 s	1.20 s
15-Ac	2.11 s	2.07 s	2.06 s	1.96 s	2.06 s	—	2.01 s
16-Ac	2.13 s	—	—	1.91 s	2.09 s	1.97 s	2.03 s
1′	4.86 d (7.2)^a^	—	4.81 d (7.8)^b^	4.85 d (7.2)	4.88 d (7.2)	4.86 d (7.2)	4.86 d (7.2)
2′	4.02 t (8.4)	—	3.97 m	4.02 t (8.4)	4.04 t (8.4)	4.02 t (8.4)	4.02 t (8.4)
3′	4.15 t (9.0)	—	4.18 m	4.15 t (9.0)	4.16 t (9.0)	4.15 t (9.0)	4.15 t (9.0)
4′	4.23 m	—	4.54 m	4.22 m	4.23 m	4.23 m	4.24 m
5′	3.73 t (8.0);	—	4.34 m; 3.72 m	3.72 m; 4.35 m	3.74 m; 4.37 m	3.72 m; 4.35 m	3.72 m; 4.35 m
	4.35 dd (4.8)						
4′-Ac	—	—	2.07 s	—	—	—	—

a Xylose.

b 4′-acetylxylose.

**TABLE 2:** ^13^C NMR Data (150 MHz, in pyridine-*d*_5_) for compounds **1**–**7** (*δ*_H_ in ppm, *J* in Hz).

Position	Compounds						
	1	2	3	4	5	6	7
1	32.6	33.0	32.6	32.8	32.6	32.9	32.8
2	30.9	31.7	30.9	32.3	30.1	31.1	32.3
3	88.8	82.8	86.7	88.8	88.7	88.8	88.7
4	41.7	40.5	40.5	41.7	41.7	41.7	41.7
5	47.7	47.8	47.8	47.7	47.6	47.7	47.4
6	21.2	21.7	21.4	21.1	21.1	21.4	20.8
7	26.5	26.5	26.4	26.5	26.1	26.7	26.5
8	48.3	48.3	48.2	48.5	48.0	50.3	49.0
9	19.9	20.1	20.1	20.2	19.8	20.7	20.2
10	26.7	27.1	26.7	26.9	27.1	26.9	26.9
11	26.3	26.6	26.5	31.2	27.0	31.9	31.2
12	37.5	25.9	26.4	216.6	39.1	216.6	216.6
13	48.6	50.3	50.3	59.3	48.3	59.0	59.1
14	47.6	47.0	47.0	42.4	46.4	42.7	42.4
15	86.4	80.3	76.7	86.1	85.7	42.7	82.4
16	79.8	78.3	80.3	80.1	79.8	77.3	79.1
17	56.1	51.6	51.6	52.7	59.0	53.2	56.5
18	21.7	14.1	33.5	64.4	26.1	64.5	64.7
19	30.5	31.2	30.4	26.1	30.5	26.2	26.5
20	85.2	86.7	82.9	86.6	87.3	86.6	87.2
21	27.6	25.1	25.1	27.7	22.3	24.4	24.7
22	33.7	41.5	41.4	39.0	39.1	39.1	32.3
23	27.0	29.0	29.0	29.7	30.5	31.2	30.9
24	83.8	111.2	111.2	81.7	115.2	81.6	114.7
25	70.9	72.4	72.4	71.7	82.8	71.7	71.6
26	27.6	25.9	25.8	26.1	30.3	26.1	26.4
27	27.6	26.5	25.9	21.7	22.3	21.5	26.3
28	26.1	25.7	25.6	21.7	26.1	21.5	26.3
29	15.8	15.3	15.6	14.5	15.7	15.8	15.7
30	13.8	14.1	14.1	13.7	13.9	14.5	15.2
15-Ac	170.9	170.7	170.6	171.5	171.2		171.5
	21.2	22.0		21.5	21.7		21.6
16-Ac	171.2			171.1	170.2	171.8	171.1
	22.0			21.6	21.8	24.4	21.4
3-Ac						76.0	
1′	108.1^a^		105.1^b^	108.1^a^	107.9^a^	108.0^a^	108.1^a^
2′	76.0		71.7	76.0	76.0	76.0	76.0
3′	79.1		76.0	79.4	79.1	79.1	79.1
4′	71.6		89.0	71.1	71.7	71.7	71.7
5′	67.6		67.6	67.6	67.6	67.6	67.6
4′-Ac			170.6 21.9				
26′					30.3		
27′					27.4		

a Xylose.

b 4′-acetylxylose.

**Actatica B (2)**: C_32_H_50_O_6_, white amorphous powder; [*α*]20 D + 19.0 (*c* = 0.15, MeOH); IR (KBr) *ν*_max_: 3,376, 2,957, 1,738, 1,373, 1,032 cm^−1^; UV (MeOH) *λ*_max_ (log *ε*): 201 nm; for ^1^H NMR (600 MHz, pyridine-*d*_5_) and ^13^C-APT (150 MHz, pyridine-*d*_5_) spectroscopic, data see Tables 1, 2; HR-ESIMS *m*/*z*: 553.3533 (calcd for C_32_H_50_O_6_Na, 553.3500).

**Actatica C (3**): C_39_H_60_O_11_, white amorphous powder; [*α*]20 D+ 35.1 (*c* = 0.31, MeOH); IR (KBr) *ν*_max_: 3,493, 2,928, 1,730, 1,375, 1,044 cm^−1^; UV (MeOH) *λ*_max_ (log *ε*): 201 nm; for ^1^H NMR (600 MHz, pyridine-*d*_5_) and ^13^C-APT (150 MHz, pyridine-*d*_5_) spectroscopic data, see Tables 1, 2; HR-ESIMS *m*/*z*: 727.4100 (calcd for C_39_ H_60_O_11_Na, [M + Na]^+^, 727.4088).

**Actatica D (4)**: C_40_H_62_O_13_, white amorphous powder; [*α*]20 D + 22.4 (*c* = 0.22, MeOH); IR (KBr) *ν*_max_: 3,439, 2,934, 1,734, 1,264, 1,033, 1,033, 962 cm^−1^; *λ*_max_ (log *ε*): 201 nm; for ^1^H NMR (600 MHz, pyridine-*d*_5_) and ^13^C-APT (150 MHz, pyridine-*d*_5_) spectroscopic data, see Tables 1, 2; HR-ESIMS *m*/*z*: 759.3974 (calcd for C_40_H_62_O_13_Na [M + Na]^+^, 759.3926).

**Actatica E (5)**: C_42_H_66_O_12_, white amorphous powder; [*α*]20 D + 42.7 (*c* = 0.35, MeOH); IR (KBr) *ν*_max_: 3,449, 2,935, 1,741, 1,375, 1,045 cm^−1^; *λ*_max_ (log *ε*): 200 nm; for ^1^H NMR (600 MHz, pyridine) and ^13^C-APT (150 MHz, pyridine-*d*_5_) spectroscopic data, see Tables 1, 2; HR-ESIMS *m*/*z*: 785.4341 (calcd for C_42_H_66_O_12_Na [M + Na]^+^, 785.4341).

**Actatica F (6)**: C_37_H_58_O_11_, white amorphous powder; [*α*]20 D + 23.3 (*c* = 0.12, MeOH); IR (KBr) *ν*_max_: 3,350, 2,820, 1,710, 1,534, 952 cm^−1^; *λ*_max_ (log *ε*): 202 nm; for ^1^H NMR (600 MHz, pyridine-*d*_5_) and ^13^C-APT (150 MHz, pyridine-*d*_5_) spectroscopic data, see Tables 1, 2; HR-ESIMS *m*/*z*: 701.3904 ([M + Na]^+^, calcd for C_37_H_58_O_11_Na, 701.3926).

**Actatica G (7)**: C_39_H_58_O_13_, white amorphous powder; [*α*]20 D + 22.6 (*c* = 0.13, MeOH); IR (KBr) *ν*_max_: 3,298, 2,781, 1,716, 1,422, 950 cm^−1^; for ^1^H NMR (600 MHz, pyridine-*d*_5_) and ^13^C-APT (150 MHz, pyridine-*d*_5_) spectroscopic data, see Tables 1, 2; HR-ESIMS *m*/*z*: 757.3765 ([M + Na]^+^, calcd for C_39_H_58_O_13_Na, 757.3770).

#### Hydrolysis of Compounds

Acid hydrolysis of **1** and **4–7**: A solution of compounds **1, 4, 5, 6, and 7** (5 mg) in 2 M HCl (1 ml) was heated at reflux for 24 h. The reaction mixture was neutralized with 2 M NaOH and extracted by partition with EtOAc (5 × 1 ml). 10 ml of water was added to the residue and extracted with CH_2_Cl_2_ three times. Sugars were analyzed by TLC and GC analysis and compared with authentic sample of D-sugars. The spots were visualized by spraying with EtOH–H_2_SO_4_–anisaldehyde (9:0.5:0.5, v/v), then heated at 150°C. Furthermore, the absolute configurations of the sugars were determined by gas chromatography according to a method previously described (Ma et al., 2016; Li et al., 2017). Compound **3** was dissolved in MeOH (15 ml), then 4% K_2_CO_3_ (15 ml) was added and each solution was stirred at room temperature overnight. Each solution was neutralized by 10% AcOH, and extracted with EtOAc (2 × 20 ml). EtOAc extract after removal of solvent, was dissolved in MeOH (10 ml) and refluxed with 0.5 N HCl (3 ml) for 4 h (Yin et al., 2010).

#### Cytotoxic Assay

The cytotoxicity of compounds **1−7** was evaluated using the MTT procedure with HT-29 and McF-7 cancer cell lines. The cells were incubated in DMEM supplemented with 10% fetal bovine serum and cultured at a density of 1.2 × 10^4^ cells/ml in a 96-well microtiter plate. Five various concentrations of each agent dissolved in dimethyl sulfoxide (DMSO) were then put in the wells. Each concentration was evaluated three times. After incubation under 5% CO_2_ at 37°C for 48 h, 10 ml of MTT (4 mg/ml) was placed into each well, and the cells were incubated for an additional 4 h. Then, the liquid was taken out, and DMSO (200 ml) was put into the wells. The absorbance was documented with a microplate reader at wave length of 570 nm.

## Results and Discussion

Compound **1** was obtained as white amorphous powder. Its IR spectrum showed absorptions of hydroxyl group at 3,440 and 3,397 cm^−1^ and carbonyl at 1,733 cm^−1^. The HRESIMS spectrum showed a pseudo-molecular ion at *m*/*z* 729.4233 [M + Na]^+^ in the positive ion mode from which in conjunction with NMR data the molecular formula was established as C_39_H_62_O_11_, consistent with nine degrees of unsaturation. In the ^1^H NMR spectrum (Table 1) two cyclopropane–methylene protons as an AX system at *δ*_H_ 0.18 and 0.47 (each 1H, d, *J* = 4.0 Hz, H_2_-19) together with seven tertiary methyl groups at *δ*_H_ 1.49, 1.47, 1.32, 1.36, 1.35, 1.02, and 1.09, indicated a cycloartane triterpenoid structure (Ju et al., 2002a; Mohamed, 2014; Gan et al., 2015; Wu et al., 2017). The ^1^H NMR spectrum also showed two oxygenated proton signals at *δ*_H_ 5.49 (d, *J* = 4.8 Hz) and *δ*_H_ 5.82 (d, *J* = 4.8 Hz), indicating two acetyl groups at C-15 and C-16. Except for sugar carbons, the ^13^C-NMR spectrum (Table 2) of **1** displayed 39 carbon resonances including methylene carbon of cyclopropane ring at *δ*_C_ 30.5 (C-19), an oxymethine carbon at *δ*_C_ 88.8 (C-3), an oxygenated quaternary carbon at *δ*_C_ 85.2 (C-20), and an anomeric carbon at *δ*_C_ 108.1, together with acetyl signals at *δ*_C_ 170.9, 171.2, 21.2, and 22.0. The ^1^H and ^13^C NMR spectroscopic data of **1** confirmed that the compound was a cycloartane triterpene glycoside (Jung et al., 2002; Wu et al., 2017; Wua et al., 2017).

**FIGURE 2:**
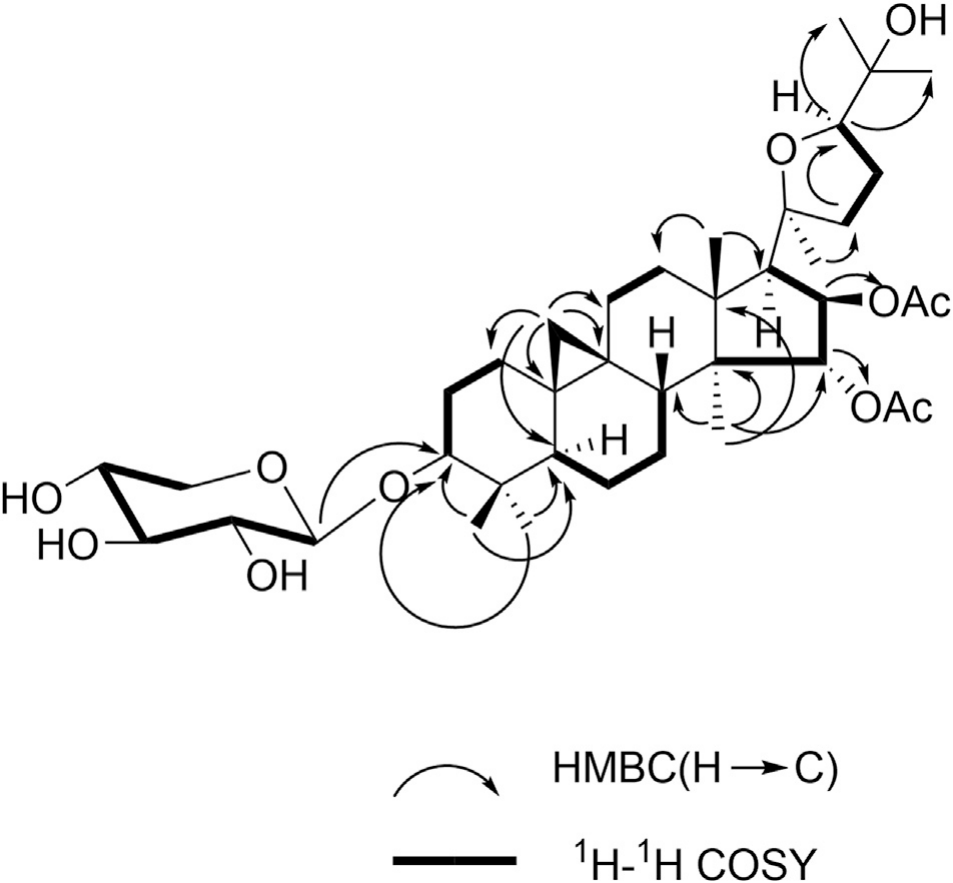
Key HMBC and ^1^H-^1^H COSY correlations for 1.

All proton signals were assigned to the corresponding carbons through direct ^1^H and ^13^C correlations in the HSQC spectrum. Inspection of the ^1^H-^1^H COSY spectrum showed fragments of C-1/C-2/C-3, C-5/C-6/C-7/C-8, C-11/C-12, C-15/C-16/C-17, and C-22/C-23/C-24. In the HMBC spectrum (Figure 2), the correlations were observed from H-28/29 to C-3 and C-5, H-19 to C-1, C-5, C-6, C-9, and C-11, and H-18 to C-12 and C-17, H-30 to C-8, C-14, C-16 and C-18, H-21 to C-22, H-22 to C-24, and H-24 to C-26 and C-2 fully confirmed the basic skeleton cycloartane triterpene of compound **1**, which was consistent with the above deduction. The acetyl groups were connected with C-15 and C-16 supported by the correlations from H-15 to *δ*_C_ 170.9 (the carbonyl carbon of OAc) and H-16 to *δ*_C_ 171.2 (the carbonyl carbon of OAc). The sugar was connected with C-3 based on the key HMBC correlation between H-1′ (*δ*_H_ 4.86, d, *J* = 7.2 Hz) and C-3 (*δ*_C_ 88.8), which was identified as D-xylose by TLC in comparison with authentic monosaccharides (visualization with ethanol-5% H_2_SO_4_ spraying) followed by gas chromatography.

**FIGURE 3:**
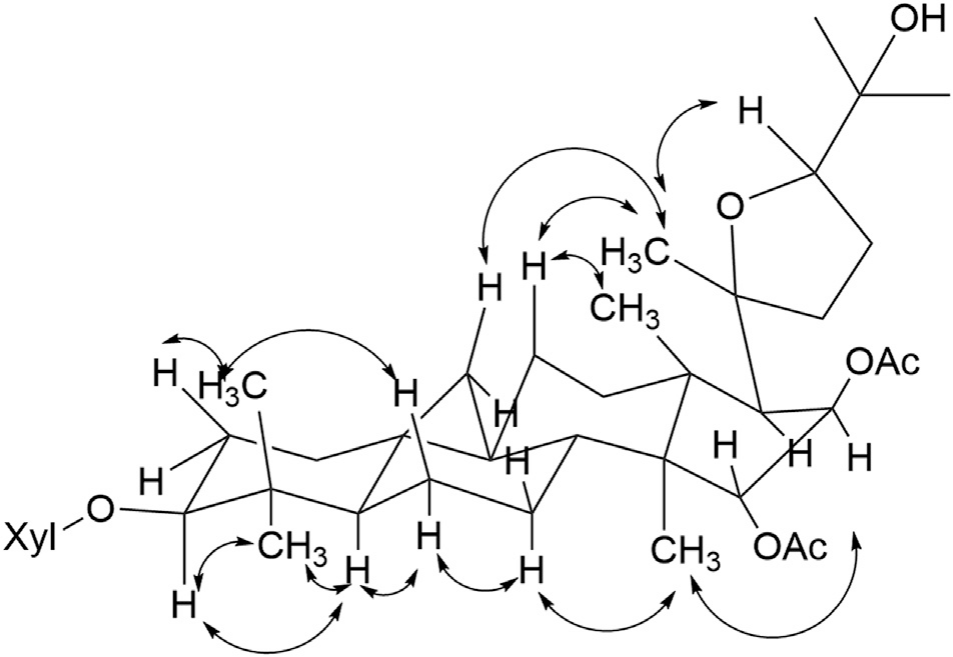
Key NOESY correlations of compound 1.

The NOESY experiment and coupling constants established the relative configuration of compound **1** (Figure 3), in which correlation of H-3/H-5 showed *α*-orientation of H-3. The larger coupling constants (^3^ *J*_1,2_ > 7.0 Hz) of the anomeric protons indicated the *β* configuration of the sugar unit. The significant cross peaks from H-15 to H_3_-18, H-17*α* to Me-21, H-16 to H_3_-30, and H-24 to Me-21 were observed, which enabled the establishment of OAc-15*α* and OAc-16*β*. Until now, all the isolated cycloartane triterpene share the identical absolute configuration with *trans* A/B, B/C, C/D rings. Considering the same cycloartane triterpene skeleton and identical carbon signals at C-20/C-24, compound **1** was established as 20*S* and 24*R* configurations (Ju et al., 2002a). Therefore, the structure of the compound was identified as shown and given the trivial name actatica A.

Compound **2** was determined to have the molecular formula C_32_H_50_O_6_, by the observation of the ion peak at *m*/*z* 553.3533 (calcd for C_32_H_50_O_6_Na, 553.3500). The ^1^H-NMR spectrum (Tables 1, 2) displayed signals for seven tertiary methyls (*δ*_H_ 1.67, 1.62, 1.54, 1.54, 1.58, 1.08, and 1.25), two typical signals at *δ*_H_ 0.32 (1H, d, *J* = 4.2 Hz) and 0.53 (1H, d, *J* = 4.2 Hz) ascribable to a cyclopropane moiety, indicating that **2** might be a cycloartane-type triterpenoid. The ^13^C NMR spectrum of 2 displayed 32 carbon signals, three signals attributable to oxygen-bearing quaternary carbons at *δ* 82.8, 111.2, and 72.4. The NMR data were similar to the reported one (20*S*, 24*S*)-16*β*, 24; 20, 24-diepoxy-9, 19-cycloeanostane-3*β*, 15*α*, 18, 25-tetraol-3-O-β-_D_-xylopyranoside (Mu et al., 2014). The differences were the absence of the sugar at C-3, and the appearance of acetyl group at C-15 in compound **2**. In the HMBC spectrum, the correlation observed from H-15 to OAc together with the molecular formula confirmed the deduction above. *The α configurations of H-16 and H-17 were confirmed by the NOESY correlations between δ*_*H*_ *1.86*(*H-17*) *and δ*_*H*_ *1.23* (*H3-30*)*, δ*_*H*_ *4.47* (*H-16*) *and δ*_*H*_ *2.00* (*H-17*)*.* Taken together with the 2D-NMR spectra data, compound 2 was characterized and named actatica B.

Compound **3**, which was isolated as a white amorphous powder, was assigned as C_39_H_60_O_11_, based on its positive HRESIMS ion at *m*/*z* 727.4100 (calcd for C_39_H_60_O_11_Na, [M + Na] ^+^, 727.4088). The ^1^H NMR spectrum showed that **3** possesses a cyclopropane ring, seven methyl groups, and an AB-type hydroxymethyl group (H_2_-18). The NMR (Tables 1, 2) spectroscopic data for this compound were analogous to **2**, except for the appearance of the anomeric proton at *δ*_H_ 4.81 (d, *J* = 7.8 Hz) and *δ*_C_ 105.1, 71.7, 76.0, 89.0, 67.6, 170.6, and 22.0. The sugar was identified as a 4′-O-β-_D_- xylose after acid hydrolysis. Inspection of the ^1^H-^1^H COSY spectrum showed fragments of C-1/C-2/C-3, C-5/C-6/C-7/C-8, C-11/C-12, C-15/C-16/C-17/C-18, and C-22/C-23. In the HMBC spectrum, the correlation from H-3 (3.36, dd, *J* = 11.4, 3.0 Hz) to the anomeric carbon signal at *δ*_C_ 86.7 supported that the sugar unit was attached to C-3. Thus, the structure of **3** was determined as actatica C.

Compound **4** has a molecular formula of C_39_H_60_O_13_ according to the HRESIMS (*m*/*z* 759.3974 [M + Na] ^+^, calcd for C_39_H_60_O_13_Na, 759.3926). Its IR spectrum showed strong hydroxyl (3,439, 1,044 cm^−1^) and carboxyl (1730 cm^−1^) absorptions. The ^1^H and ^13^CNMR spectra indicated that **4** had two acetoxyl groups. Detailed NMR spectral analysis revealed that **4** possessed a cyclopropane ring, six methyl groups, a hydroxymethyl group at C-18, and a D-xylosyl unit at C-3. The ^1^H and ^13^C NMR spectra of **4** were similar to those of beesioside J (Ju et al., 2002b), except for a carbonyl group (C=O) connected to C-12 of **4**, which causes the downfield chemical shift of C-12 (*δ*_C_ 216.6). The correlation from *δ*_H_ 4.54 (H-11) to *δ*_C_ 216.6 (C=O) according to the HMBC supported the above result. Therefore, compound **4** was tentatively determined and named actatica D.

Compound **5** has the molecular formula C_42_H_66_O_12_ determined by HR-ESIMS (*m*/*z* 785.4301, calcd for C_42_H_66_O_12_Na [M + Na] ^+^, 785.4341). In the ^1^H NMR spectrum (Table 1) two cyclopropane–methylene protons as an AX system at *δ*_H_ 0.21 and 0.59 (each 1H, d, *J* = 4.0 Hz, H_2_-19) together with nine tertiary methyl groups indicated a cycloartane triterpenoid structure. The ^1^H NMR and ^13^C APT data for this compound were analogous to 1, except for the additional NMR signals at *δ*_C_ 30.3 and 27.4, and *δ*_H_ 1.31 (3H, s), and 1.45 (3H, s). The differences showed that **5** had one more hydroxyisopropyl group connected at C-24. In the HMBC spectrum, the correlations from H-24 to C-26, C-26′, C-27, and C-27′ confirmed the above deduction. Taken together with the NOESY spectra data, compound **5** was established as 24*R* configurations. Thus, compound **5** was established and named actatica E.

Compound **6** was determined to have the molecular formula of C_37_H_58_O_11_ based on the ^13^C APT data and by the HRESIMS ion peak at *m*/*z* 701.3904 ([M + Na] ^+^, calcd for C_37_H_58_O_11_Na, 701.3926). The ^1^H-NMR spectrum (Table 1) displayed signals for seven tertiary methyls (*δ* 1.02, 1.22, 1.23, 1.30, 1.53, and 1.55), two typical signals at *δ* 0.12 (1H, d, *J* = 4.2 Hz) and 0.44 (1H, d, *J* = 4.2 Hz) ascribable to a cyclopropane methylene group, indicating that **6** might be a cycloartane-type triterpenoid. Examination of the ^1^H and ^13^C APT data (Tables 1, 2) showed the structure of **6** to be similar to **4**. The NMR spectrum showed that compound 6 has only one set of acetyl group data. On the basis of ^1^H-^1^H COSY and HSQC and comparison with related **4**, all signals were assigned as shown in Tables 1, 2. The correlation from H-16 (*δ*_H_ 5.47) to acetyl carbon (*δ*_C_ 171.8) was observed in HMBC spectrum, which means the acetyl group was connected to C-16. Therefore, compound **6** was clearly determined and named actatica F.

Compound **7** has the molecular formula C_39_H_58_O_13_ according to its HRESIMS result (*m*/*z* 757.3765 [M + Na] ^+^, calcd for C_39_H_58_O_13_Na, 757.3770). In the ^1^H NMR spectrum (Table 1) two cyclopropane-methylene protons as an AX system at *δ*_H_ 0.28 and 0.41 (each 1H, d, *J* = 4.2 Hz, H_2_-19) together with nine tertiary methyl groups indicated a cycloartane triterpenoid structure. The ^1^H NMR and ^13^C APT data were closely related to those of beesioside I (Tables 1, 2) (Sakurai et al., 1990). The differences showed that **7** had a carbonyl group attached to C-12, which caused C-12 to move to a lower field, and the chemical shift is greatly increased to *δ*_C_ 216.6). The HMBC spectrum shows that *δ*_H_ 4.54 (H-11) is related to *δ*_C_ 216.6 (C = O), confirming the above inference. Moreover, in the NOESY spectrum, correlations were also detected between Me-21/H-22*α*/H-23*α*/H-24*α*, H-22*α*/H-22*β*, H-23*α*/H-23*β*, H-22*β*/H-23*β*, and H-24*α*/Me-26/Me-27. Considering the same cycloartane triterpene skeletonand identical carbon signals at C-20/C-24, compound **7** enabled a determination of a 20S*, 24R* configuration (Ju et al., 2016). As a result, the structure of **7** was established and named actatica G.

### Bioactive Activity

The cytotoxic of all compounds **1–7** were tested for their inhibitory activity against human HT-29 and McF-7 cancer cell lines using MTT assay. All compounds showed significant inhibitory effects with IC_50_ values of 9.2–26.4 μM (Table 3). Compound 7, with an oxygen bridge between C-18 and C-24, showed the best potency among the isolated constituents. With a tetrahydrofuran fragment connected by C-20 and C-24, compounds **1** and **4–7** showed better activity than **2** and **3**.

**TABLE 3:** Cytotoxicity of compounds **1**–**7** against HT-29 and McF-7 cancer cell lines.

Compounds	HT-29 (μM)	McF-7 (μM)
1	10.4 ± 1.9^a^	11.8 ± 2.6
2	24.6 ± 2.6	26.4 ± 1.8
3	21.7 ± 2.3	23.2 ± 1.6
4	12.6 ± 2.8	12.1 ± 1.5
5	11.0 ± 1.1	23.9 ± 3.0
6	17.5 ± 2.2	12.3 ± 0.7
7	9.2 ± 3.0	11.4 ± 1.9
5-FU^b^	3.0 ± 2.1	2.3 ± 1.2

a Value present mean ± SD of triplicate experiments.

b Positive control substance.

Seven new 9,19-cycloartane glycosides were isolated from the rhizomes of *A. asiatica* H. Hara. Until now, nearly 200 naturally occurring triterpenes with a 9,19-cycloartane have been reported (Su et al., 2016; Hassan et al., 2020). However, compound **5** with one more hydroxy isopropyl group was first isolated from the genus *Actaea*. All compounds displayed inhibitory activity against human HT-29 and McF-7 cancer cell lines. Further analysis of the data showed that compounds **1** and **4–7** exhibited better protective effect than other compounds, which indicated that the tetrahydrofuran fragment connected by C-20 and C-24 may affect the inhibitory activity regarding HT-29 and McF-7.

## Data Availability

The original contributions presented in the study are included in the article/Supplementary Material; further inquiries can be directed to the corresponding authors.

## References

[B1] Fan Y. S. Yao Z. Teng J. Pan Q. Duan H. Q. (2007). Triterpenoids from Actaea Asiatica with Antitumor Activity. Chin. Traditional Herbal Drugs 38 (2), 167–170. 10.3321/j.issn:0253-2670.2007.02.003

[B2] Gan L.-S. Zheng D.-J. Liu Q. Zhou J. Zhang M.-Z. Yao W. (2015). Eight New Cycloartane Triterpenoids from Beesia Calthifolia with Hepatoprotective Effects against D-Galactosamine Induced L02 Cell Damage. Bioorg. Med. Chem. Lett. 25 (18), 3845–3849. 10.1016/j.bmcl.2015.07.070 26238319

[B3] Gao J.-C. Zhang J.-C. Lu Z.-J. Zhu G.-Y. Yang M.-S. Xiao P.-G. (2006b). Chemical Constituents of Actaea Asiatica Hara and Their Anti-osteoporosis Activities. Biochem. Syst. Ecol. 34 (9), 710–713. 10.1016/j.bse.2006.02.004

[B4] Gao J.-C. Zhang J.-C. Zhu G.-Y. Yang M.-S. Xiao P.-G. (2007). Chromones and Indolinone Alkaloids from Actaea Asiatica Hara. Biochem. Syst. Ecol. 35 (7), 467–469. 10.1016/j.bse.2007.01.012

[B5] Gao J. Huang F. Zhang J. Zhu G. Yang M. Xiao P. (2006a). Cytotoxic Cycloartane Triterpene Saponins fromActaeaasiatica. J. Nat. Prod. 69 (10), 1500–1502. 10.1021/np060113h 17067171

[B6] Hassan A. R. Ashour A. Amen Y. Nagata M. El-Toumy S. A. Shimizu K. (2020). A New Cycloartane Triterpene and Other Phytoconstituents from the Aerial Parts of Euphorbia Dendroides. Nat. Product. Res. 1–9. 10.1080/14786419.2020.1800693 32722993

[B7] Ju J.-H. Liu D. Lin G. Xu X. D. Han B. Yang J.-S. (2002a). Beesiosides A−F, Six New Cycloartane Triterpene Glycosides from Beesia Calthaefolia. J. Nat. Prod. 65 (1), 42–47. 10.1021/np010293p 11809062

[B8] Ju J.-H. Liu D. Lin G. Zhang Y.-M. Yang J.-S. Lu Y. (2002b). Beesiosides G, H, and J−N, Seven New Cycloartane Triterpene Glycosides fromBeesiacalthifolia. J. Nat. Prod. 65 (2), 147–152. 10.1021/np010294h 11858746

[B9] Jung D.-W. Lee J. M. Sung C. K. (2002). Enzyme-linked Immunosorbent Assay for the Determination of 20(S)-protopanaxatriol. Analytica Chim. Acta 462 (2), 157–163. 10.1016/S0003-2670(02)00340-9

[B10] Kusano A. Takahira M. Shibano M. Miyase T. Kusano G. Bulletin P. (1999). Studies on the Constituents of Cimicifuga Species. XXVI. Twelve New Cyclolanostanol Glycosides from the Underground Parts of Cimicifuga Simplex WORMSK. Chem. Pharm. Bull. 47 (4), 511–516. 10.1248/cpb.47.511

[B11] Kusano A. Takahira M. Shibano M. Miyase T. Okuyama T. Kusano G. (1998). ChemInform Abstract: Studies on the Constituents of Cimicifuga Species. Part 22. Structures of Two New Cyclolanostanol Xylosides, Cimiacerosides A and B. ChemInform 5 (48), 1003–1013. 10.1002/chin.199839202

[B12] Li P. Zhu N. Hu M. Wu H. Yu T. Wu T. (2017). New Cucurbitane Triterpenoids with Cytotoxic Activities from Hemsleya Penxianensis. Fitoterapia 120, 158–163. 10.1016/j.fitote.2017.06.009 28625732

[B13] Ma G.-X. Feng W. Sun Z.-H. Li P.-F. Zhu N.-L. Yang J.-S. (2016). New Stigmastane Type of Steroidal Glycosides from the Roots of Vernonia Cumingiana. J. Carbohydr. Chem. 35 (3), 172–179. 10.1080/07328303.2016.1170137

[B14] Mohamed G. A. (2014). New Cytotoxic Cycloartane Triterpene from Cassia Italica Aerial Parts. Nat. Product. Res. 28 (13), 976–983. 10.1080/14786419.2014.902820 24684761

[B15] Mu L.-H. Li H.-J. Guo D.-H. Zhao J.-Y. Liu P. (2014). Cycloartane Triterpenes from Beesia Calthaefolia (Maxim.). Fitoterapia 92, 41–45. 10.1016/j.fitote.2013.10.005 24144800

[B16] Sakurai N. Goto T. Nagai M. Inoue T. Xiao P. (1990). Studies on the Constituents of Beesia Calthaefolia, and Souliea Vaginata. III, Breesiodide IV, a Cyclolanostanol Xyloside from the Rhizomes of B. Calthaefolia and S. Vaginata. Heterocycles 30 (2), 897–904. 10.3987/COM-89-S78

[B17] Su Y. Chi W.-C. Wu L. Wang Q.-H. Kuang H.-X. (2016). Photochemistry and Pharmacology of 9, 19-cyclolanostane Glycosides Isolated from Genus Cimicifuga. Chin. J. Nat. Medicines 14 (10), 721–731. 10.1016/S1875-5364(16)30087-5 PMC7129281 28236402

[B18] Wu H.-F. Liu X. Zhu Y.-D. Zhou J. Gong Y.-Y. Ma G.-X. (2017). A New Cycloartane Triterpenoid Glycoside from Souliea Vaginata. Nat. Product. Res. 31 (21), 2484–2490. 10.25135/rnp.10.17.06.10310.1080/14786419.2017.1314283 28403639

[B19] Wua H. Yang Z. Wang Q. Zhub N. Xub X. Zou Q. (2017). A New Cytotoxic Cyclolanostane Triterpenoid Xyloside from Souliea Vaginata. Nat. Prod. Commun. 12 (2), 229–232. 10.1177/1934578X1701200222 30428218

[B20] Yin N. Yan-Li Z. Chen J.-C. Lu L. Qiu M.-H. Qiug C. (2010). Cytotoxic Chemical Constituents from the Roots of *Cimicifuga Fetida* . J. Nat. Prod. 73 (2), 1192. 10.1021/np9003855 20121210

