# Supplementary material for: Actaticas A−G, Cycloartane Triterpenes From Actaea asiatica With Their Antiproliferative Activity
Source: Front Chem. 2021 Jul 29;9:695456. doi: 10.3389/fchem.2021.695456 (PMC8358065; doi:10.3389/fchem.2021.695456)
Supplement: Supplementary file 1 [file DataSheet1.docx]

**Supporting Information**

Actaticas A-G, new triterpenes from *Actaea asiatica* with their potential antiproliferative activity

Key Laboratory of Bioactive Substances and Resource Utilization of Chinese Herbal Medicine, Ministry of Education, Beijing Key Laboratory of Innovative Drug Discovery of Traditional Chinese Medicine (Natural Medicine) and Translational Medicine, Key Laboratory of Efficacy Evaluation of Chinese Medicine against Glycolipid Metabolic Disorders,State Administration of Traditional Chinese Medicine, Institute of Medicinal Plant Development, Peking Union Medical College and Chinese Academy of Medical Sciences，Beijing 100193, China

Figure S1 ^1^H NMR (600 MHz, C5D5N) spectrum of **1** ................................................................... 4

Figure S2 ^13^C-APT (150 MHz, C5D5N) spectrum of **1** ............................................................. 4

Figure S3 ^1^H- ^1^H COSY (C5D5N) spectrum of **1** .................................................................... 5

Figure S4 HMBC (C5D5N) spectrum of **1** ................................................... 5

Figure S5 HSQC (C5D5N) spectrum of **1** ........................................................................................ 6

Figure S6 NOESY (C5D5N) spectrum of **1** ........................................................................................ 6

Figure S7 UV spectrum of **1** ........................................................................................ 7

Figure S8 HRESIMS spectrum of **1** ........................................................................................ 8

Figure S9 IR spectrum of **1** ........................................................................................ 8

Figure S10 ^1^H NMR (600 MHz, C5D5N) spectrum of **2** ........................................................... 9

Figure S11 ^13^C-APT (150 MHz, C5D5N) spectrum of **2** .................................................................... 9

Figure S12 ^1^H -^1^H COSY (C5D5N) spectrum of **2** ................................................................... 10

Figure S13 HSQC (C5D5N) spectrum of **2** ............................................................................................. 10

Figure S14 HMBC (C5D5N) spectrum of **2** ......................................................................................... 11

Figure S15 NOESY (C5D5N) spectrum of **2** .............................................................................. 11

Figure S16 UV spectrum of **2** ........................................................................................ 12

Figure S17 HRESIMS spectrum of **2** ........................................................................................ 13

Figure S18 IR spectrum of **2** ........................................................................................ 13

Figure S19 ^1^H NMR (600 MHz, C5D5N) spectrum of **3** ..........................................................................14

Figure S20 ^13^C-APT (150 MHz, C5D5N) spectrum of **3** ................................................................. 14

Figure S21 ^1^H -^1^H COSY (C5D5N) spectrum of **3** .................................................................................. 15

Figure S22 HSQC (C5D5N) spectrum of **3** ............................................................................................. 15

Figure S23 HMBC (C5D5N) spectrum of **3** .......................................................................... 16

Figure S24 NOESY (C5D5N) spectrum of **3** ................................................... 16

Figure S25 UV spectrum of **3** ........................................................................................ 17

Figure S26 HRESIMS spectrum of **3** ........................................................................................ 18

Figure S27 IR spectrum of **3** ........................................................................................ 18

Figure S28 ^1^H NMR (600 MHz, C5D5N) spectrum of **4** .................................................... 19

Figure S29 ^13^C-APT (150 MHz, C5D5N) spectrum of **4** ................................................... 19

Figure S30 ^1^H- ^1^H COSY (C5D5N) spectrum of **4** ................................................................. 20

Figure S31 HSQC (C5D5N) spectrum of **4** ........................................................... 20

Figure S32 HMBC (C5D5N) spectrum of **4** ................................................... 21

Figure S33 NOSEY (C5D5N) spectrum of **4** ................................................... 21

Figure S34 UV spectrum of **4**........................................................................................ 22

Figure S35 HRESIMS spectrum of **4** ........................................................................................ 23

Figure S36 IR spectrum of **4** ........................................................................................ 23

Figure S37 ^1^H NMR (600 MHz, C5D5N) spectrum of **5** ........................................................ 24

Figure S38 ^13^C-APT (150 MHz, C5D5N) spectrum of **5** ....................................................... 24

Figure S39 ^1^H- ^1^H COSY (C5D5N) spectrum of **5** .................................................................... 25

Figure S40 HSQC (C5D5N) spectrum of **5** ........................................................................ 25

Figure S41 HMBC (C5D5N) spectrum of **5** .................................................................................... 26

Figure S42 NOESY (C5D5N) spectrum of **5** ................................................... 26

Figure S43 UV spectrum of **5** ........................................................................................ 27

Figure S44 HRESIMS spectrum of **5** ........................................................................................ 28

Figure S45 IR spectrum of **5**........................................................................................ 28

Figure S46 ^1^H NMR (600 MHz, C5D5N) spectrum of **6** ............................................................... 29

Figure S47 ^13^C-APT (150 MHz, C5D5N) spectrum of **6** ................................................... 29

Figure S48 ^1^H- ^1^H COSY (C5D5N) spectrum of **6** ................................................................ 30

Figure S49 HSQC (C5D5N) spectrum of **6** ................................................................................... 30

Figure S50 HMBC (C5D5N) spectrum of **6** ........................................................................................... 31

Figure S51 NOESY (C5D5N) spectrum of **6** ...................................................................... 31

Figure S52 UV spectrum of **6** ........................................................................................ 32

Figure S53 HRESIMS spectrum of **6** ........................................................................................ 33

Figure S54 IR spectrum of **6** ........................................................................................ 33

Figure S55 ^1^H NMR (600 MHz, C5D5N) spectrum of **7** ........................................................................... 34

Figure S56 ^13^C-APT (150 MHz, C5D5N) spectrum of **7** .................................................................... 34

Figure S57 ^1^H- ^1^H COSY (C5D5N) spectrum of **7** ............................................................... 35

Figure S58 HSQC (C5D5N) spectrum of **7** ...................................................................................... 35

Figure S59 HMBC (C5D5N) spectrum of **7** .................................................................................................36

Figure S60 NOESY (C5D5N) spectrum of **7** ...................................................................... 36

Figure S61 UV spectrum of **7** ........................................................................................ 37

Figure S62 HRESIMS spectrum of **7** ........................................................................................ 38

Figure S63 IR spectrum of **7** ........................................................................................ 38


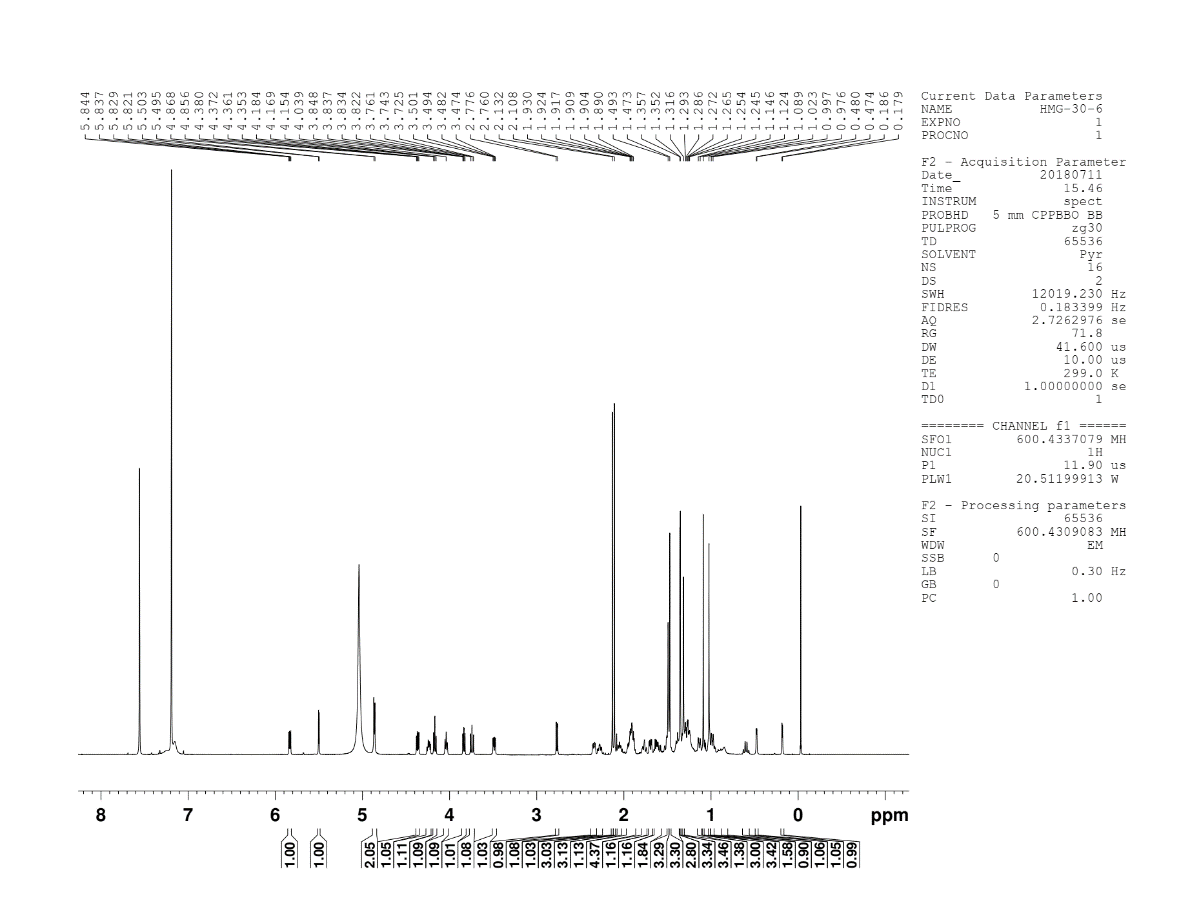


**Figure S1** ^1^H NMR (600 MHz, C5D5N) spectrum of **1**


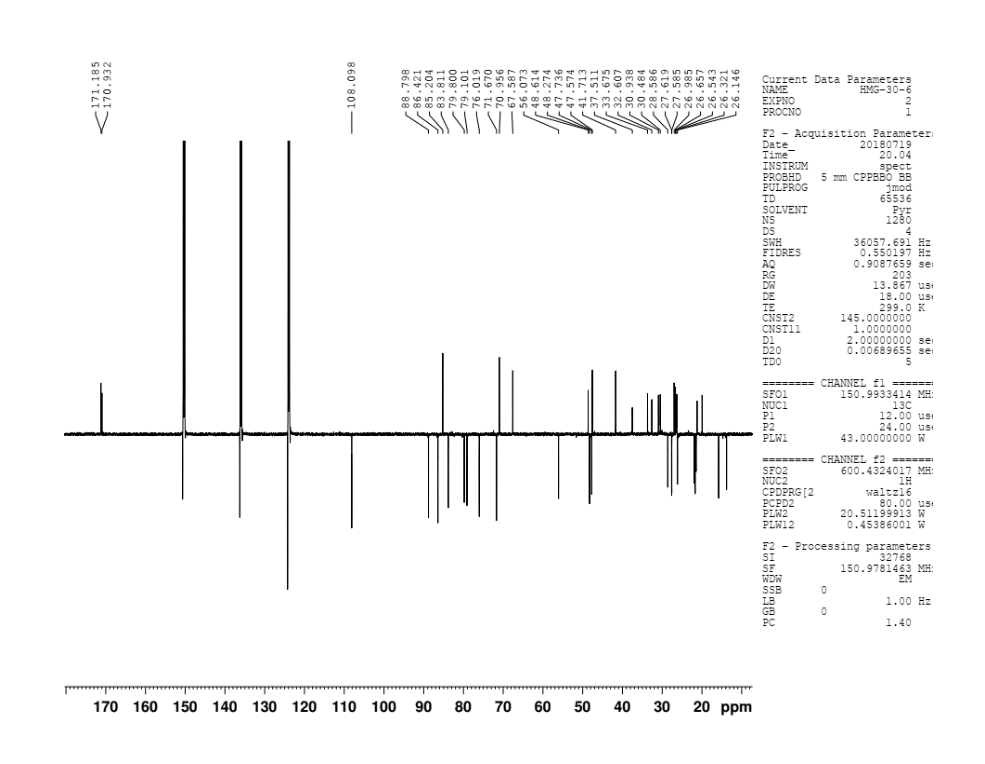


**Figure S2** ^13^C-APT (150 MHz, C5D5N) spectrum of **1**


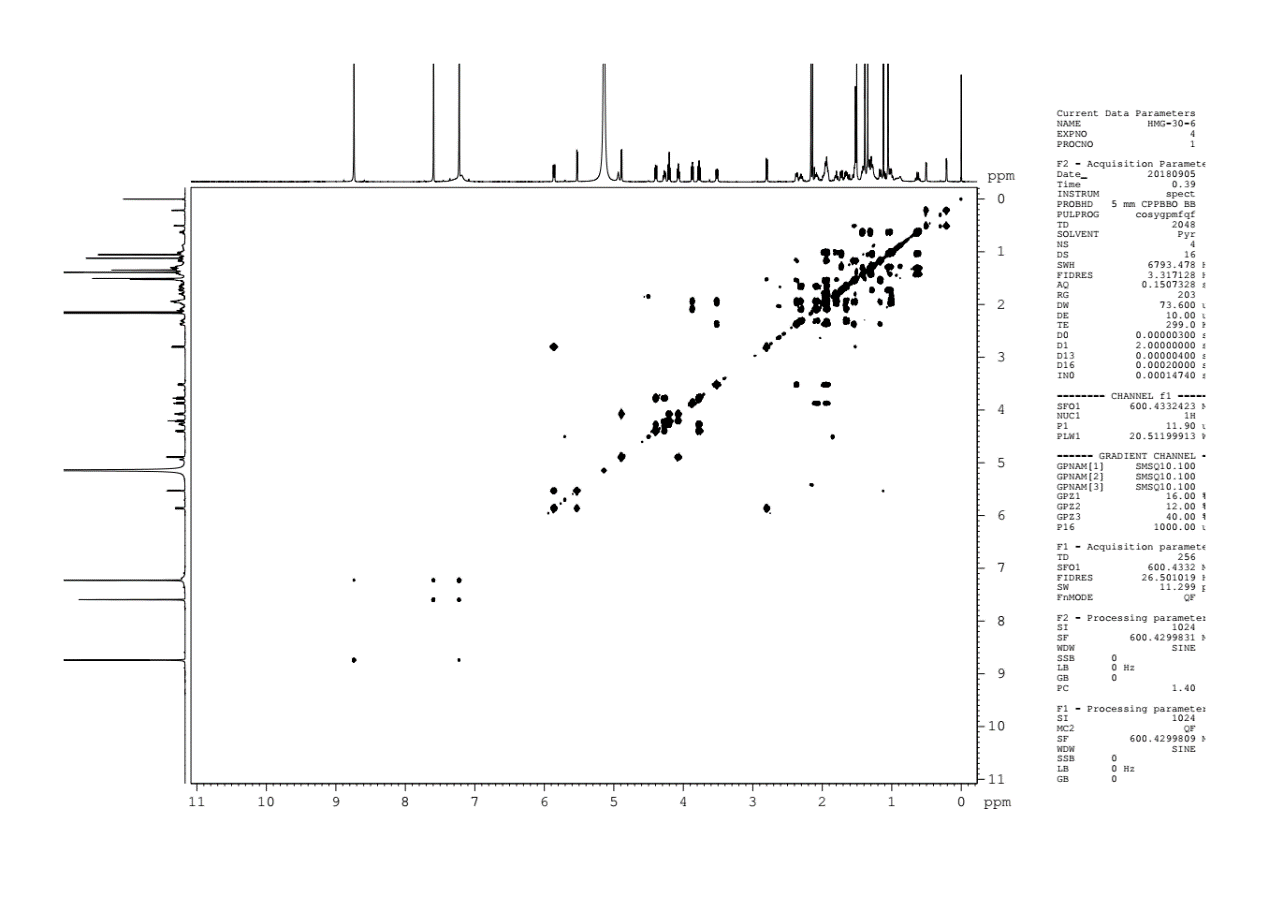


**Figure S3** ^1^H- ^1^H COSY (C5D5N) spectrum of **1**


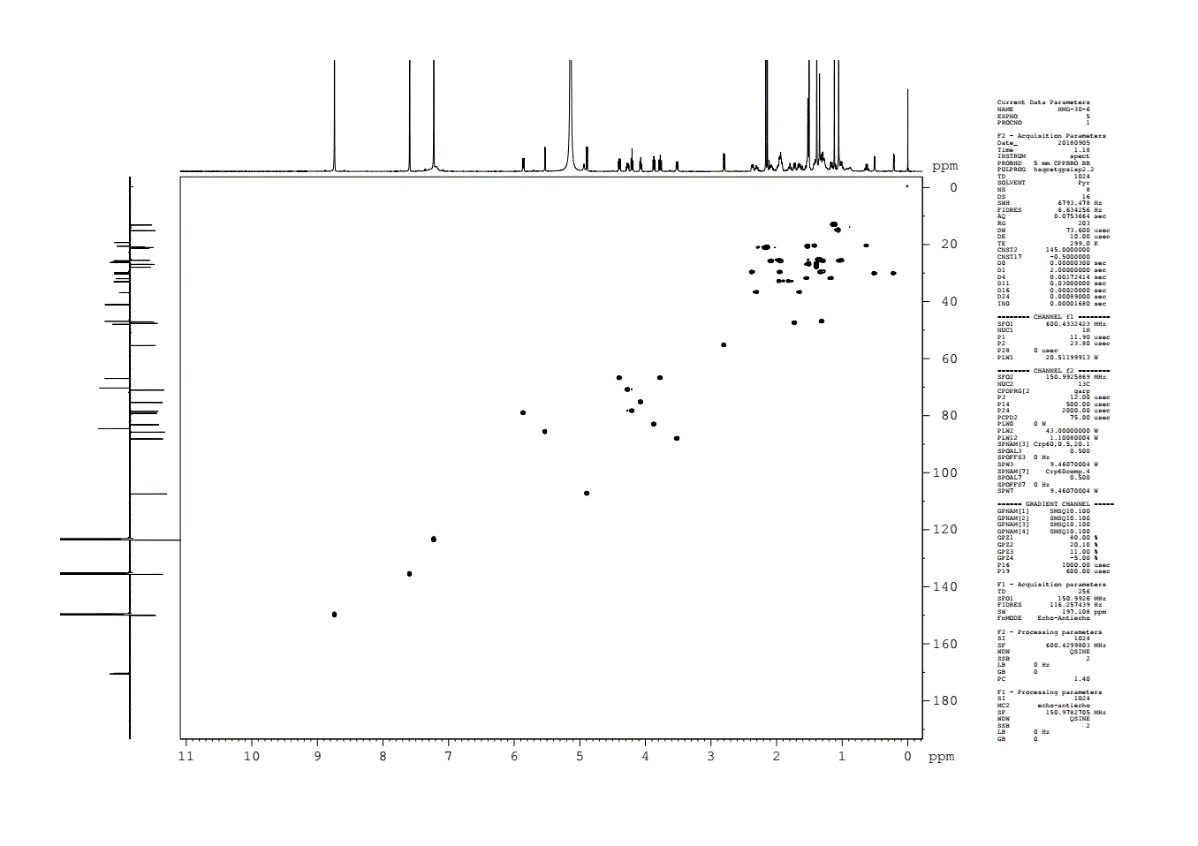


**Figure S4** HSQC (C5D5N) spectrum of **1**


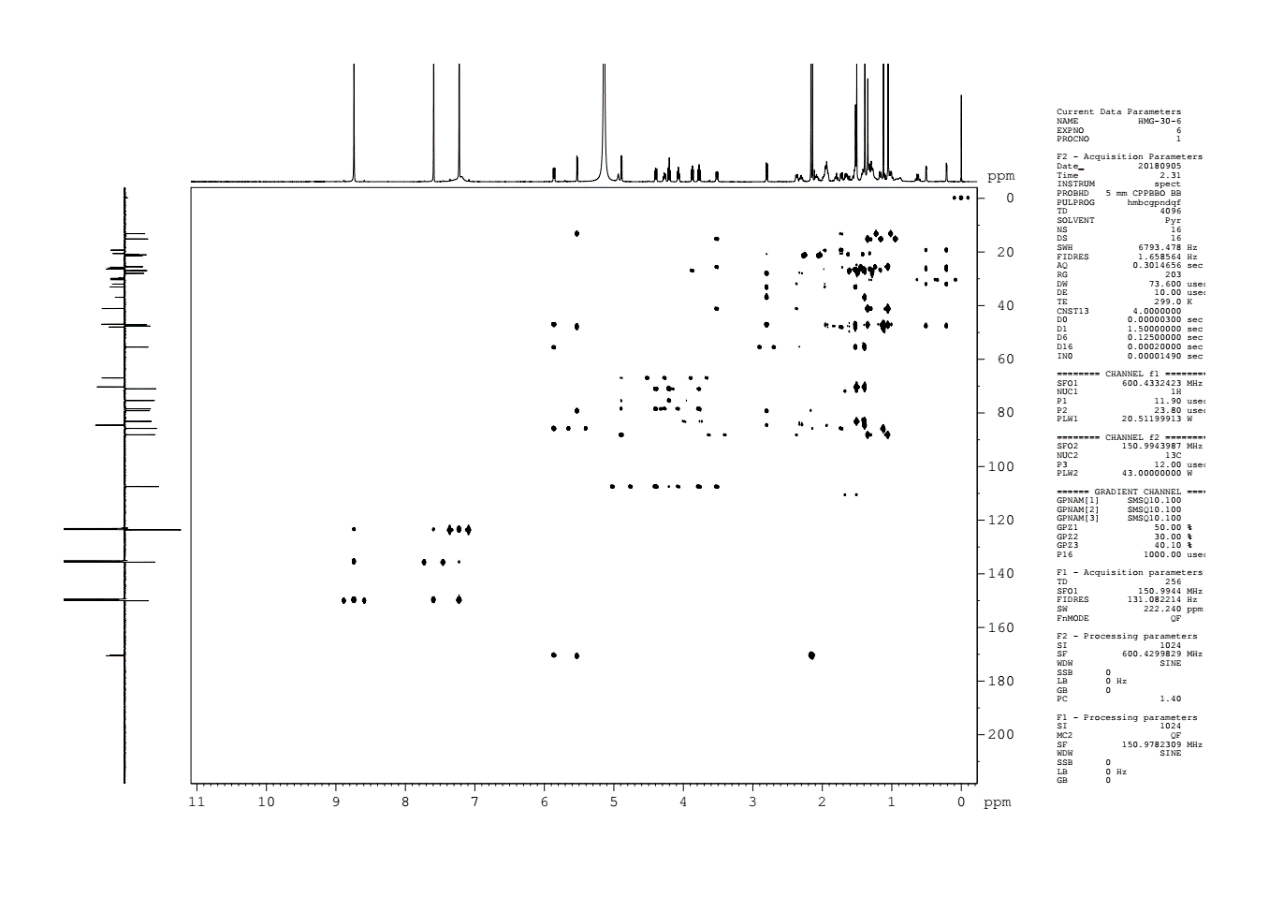


**Figure S5** HMBC (C5D5N) spectrum of **1**


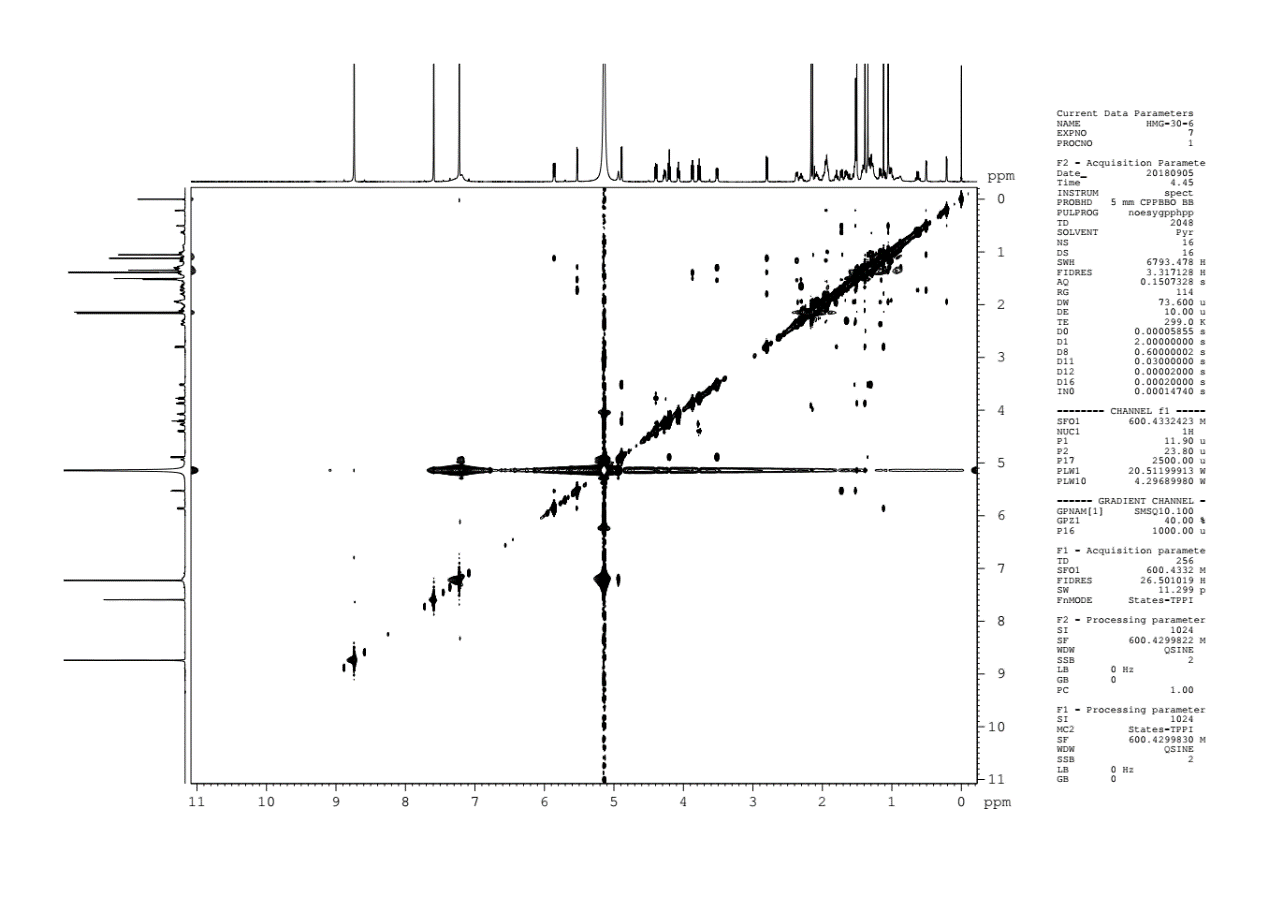


**Figure S6** NOESY (C5D5N) spectrum of **1**


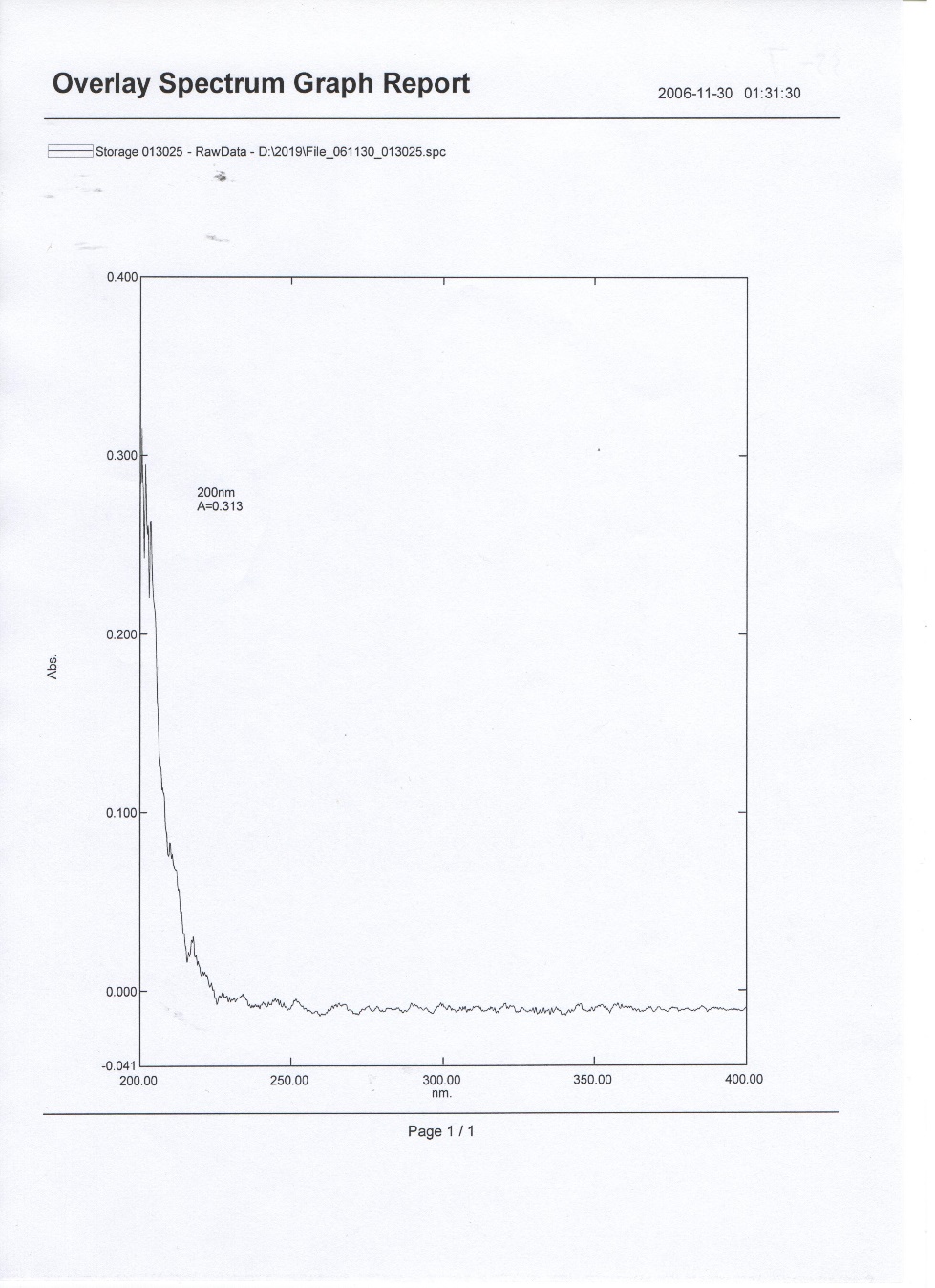


**Figure S7** UV spectrum of **1**


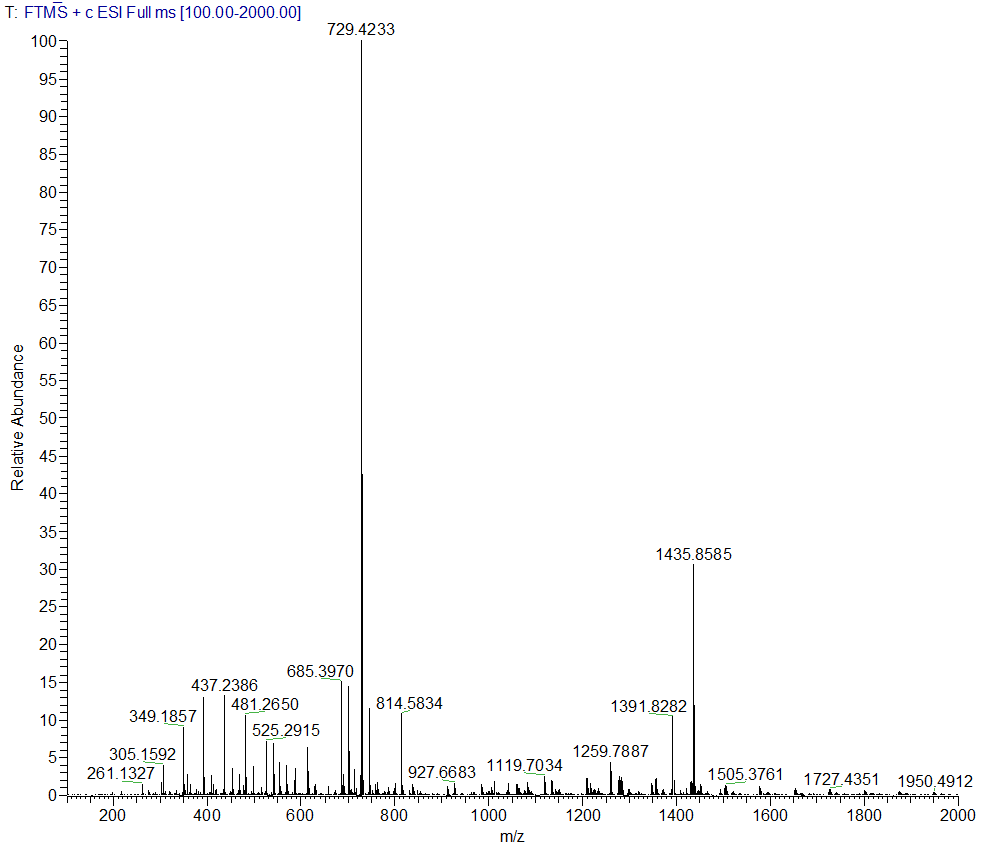


**Figure S8** HRESIMS spectrum of **1**


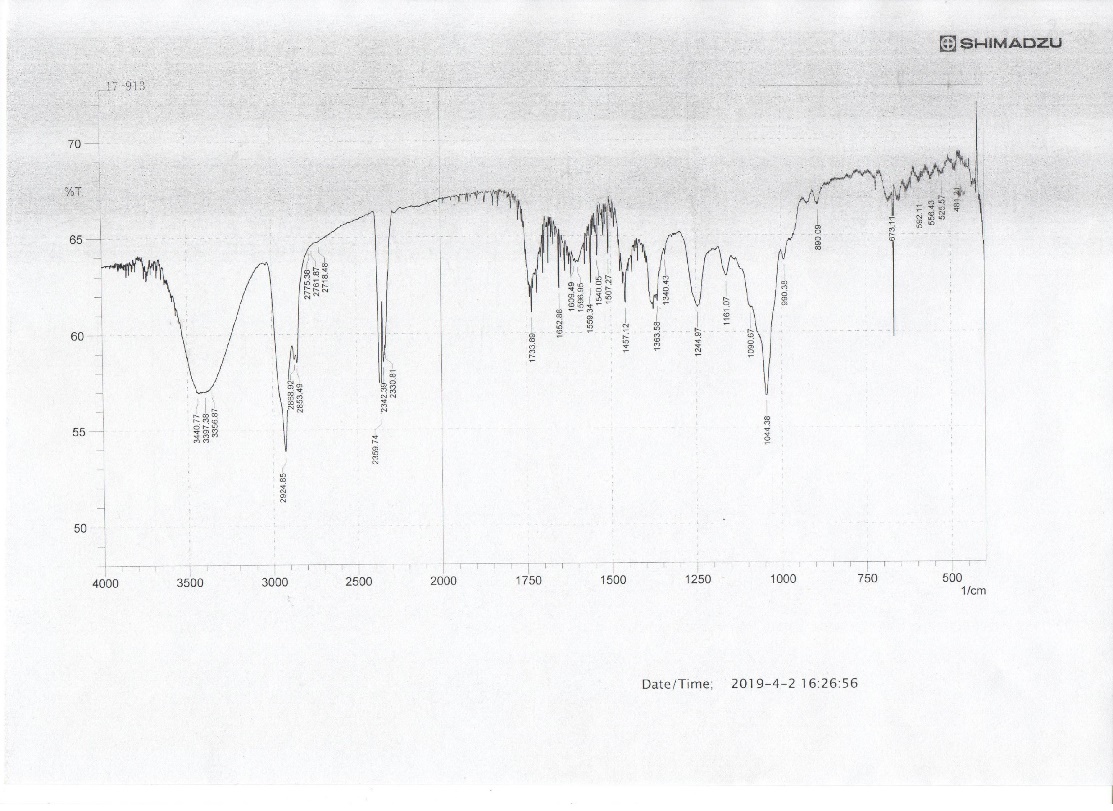


**Figure S9** IR spectrum of **1**


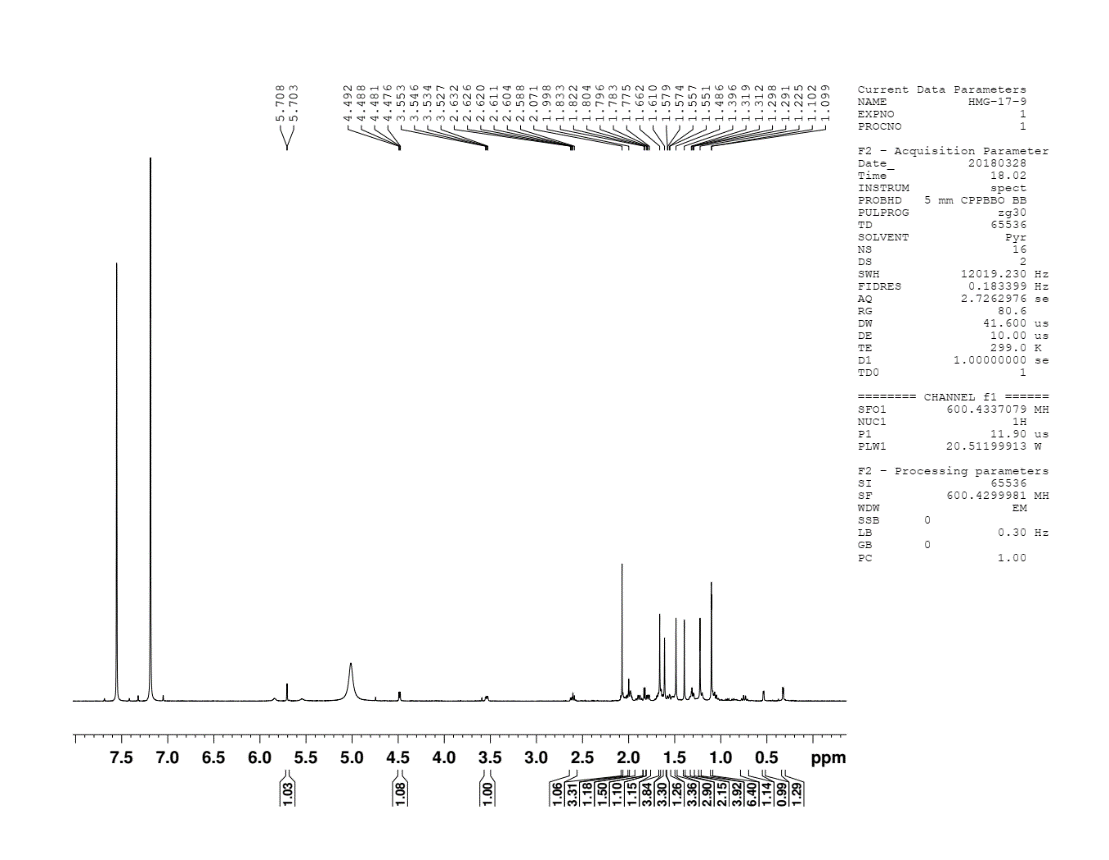


**Figure S10** ^1^H NMR (600 MHz, C5D5N) spectrum of **2**


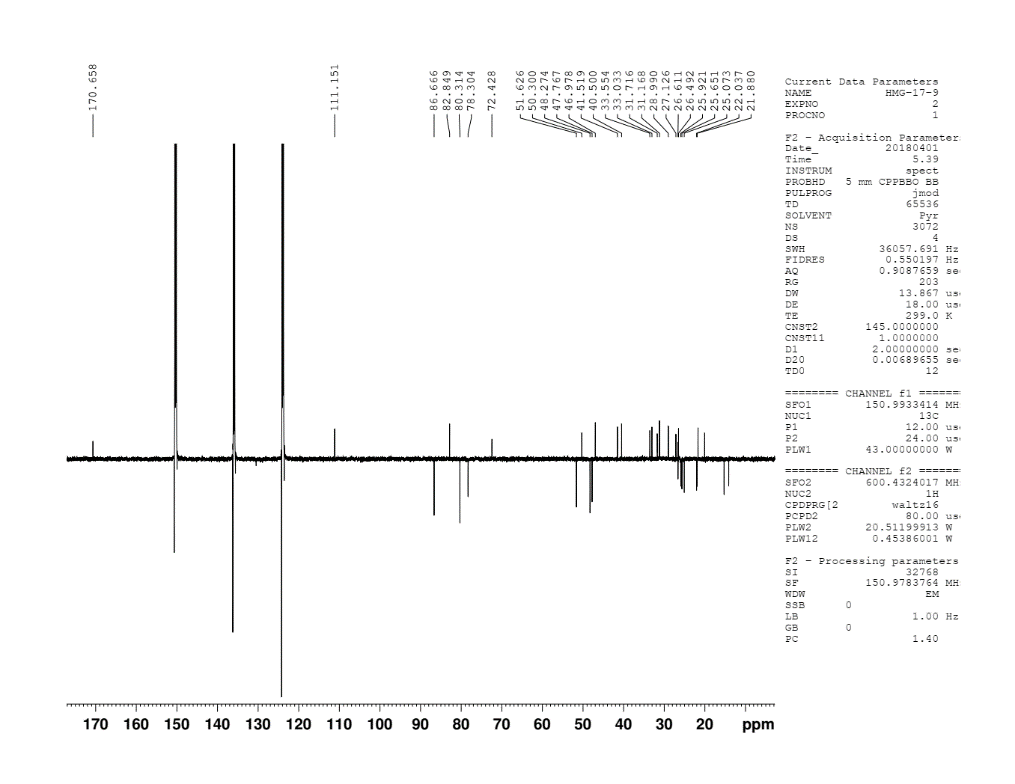


**Figure S11** ^13^C-APT (150 MHz, C5D5N) spectrum of **2**


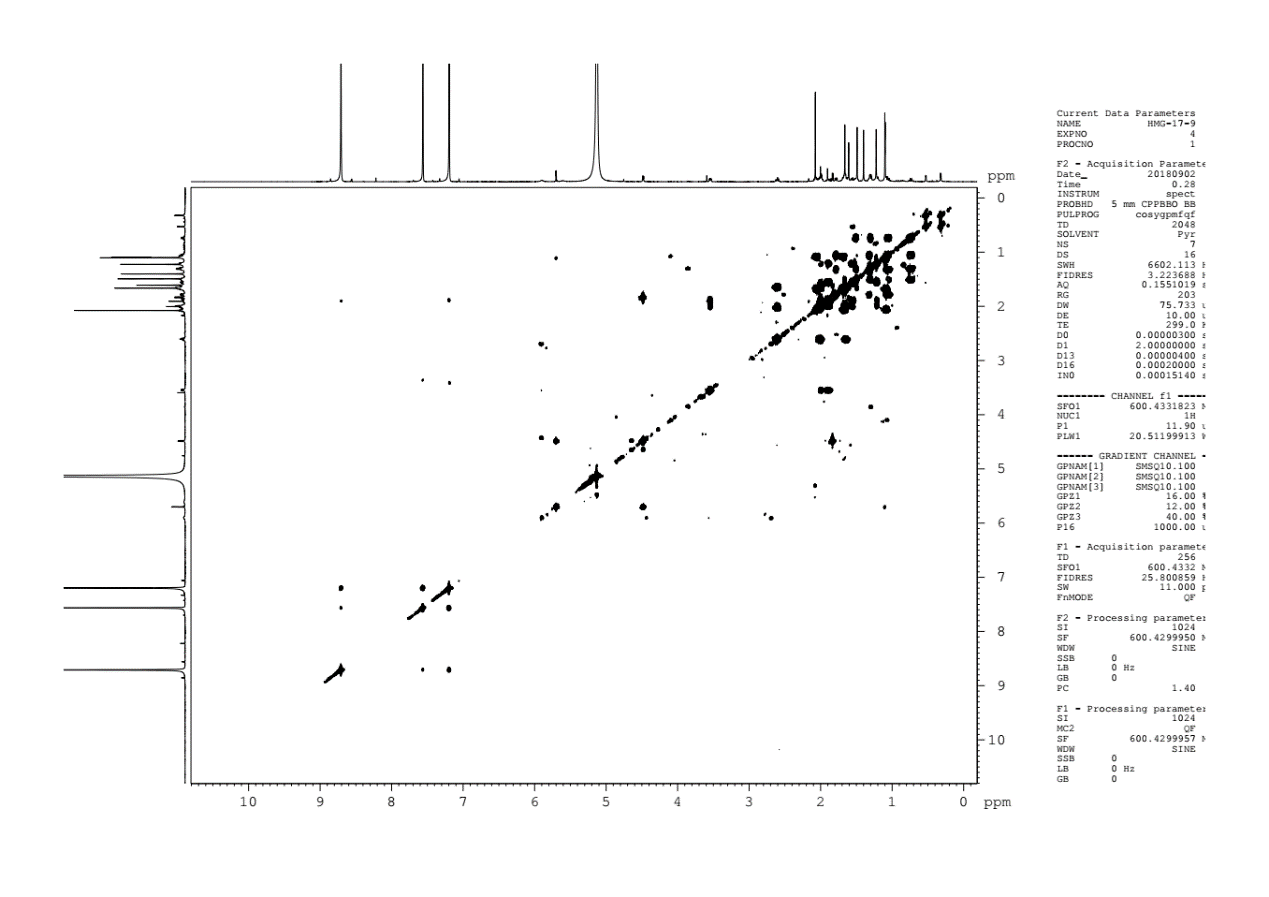


**Figure S12** ^1^H -^1^H COSY (C5D5N) spectrum of **2**


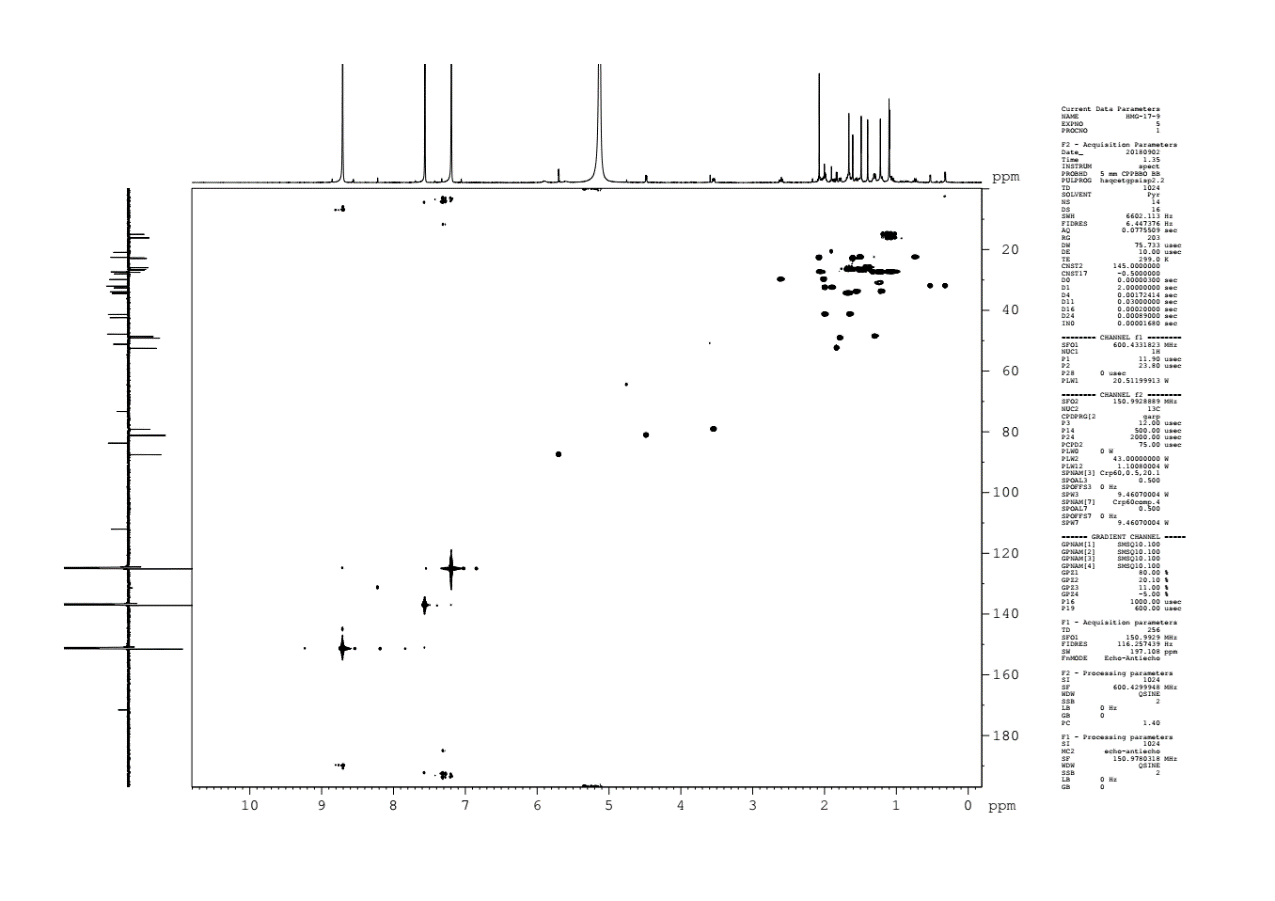


**Figure S13** HSQC (C5D5N) spectrum of **2**


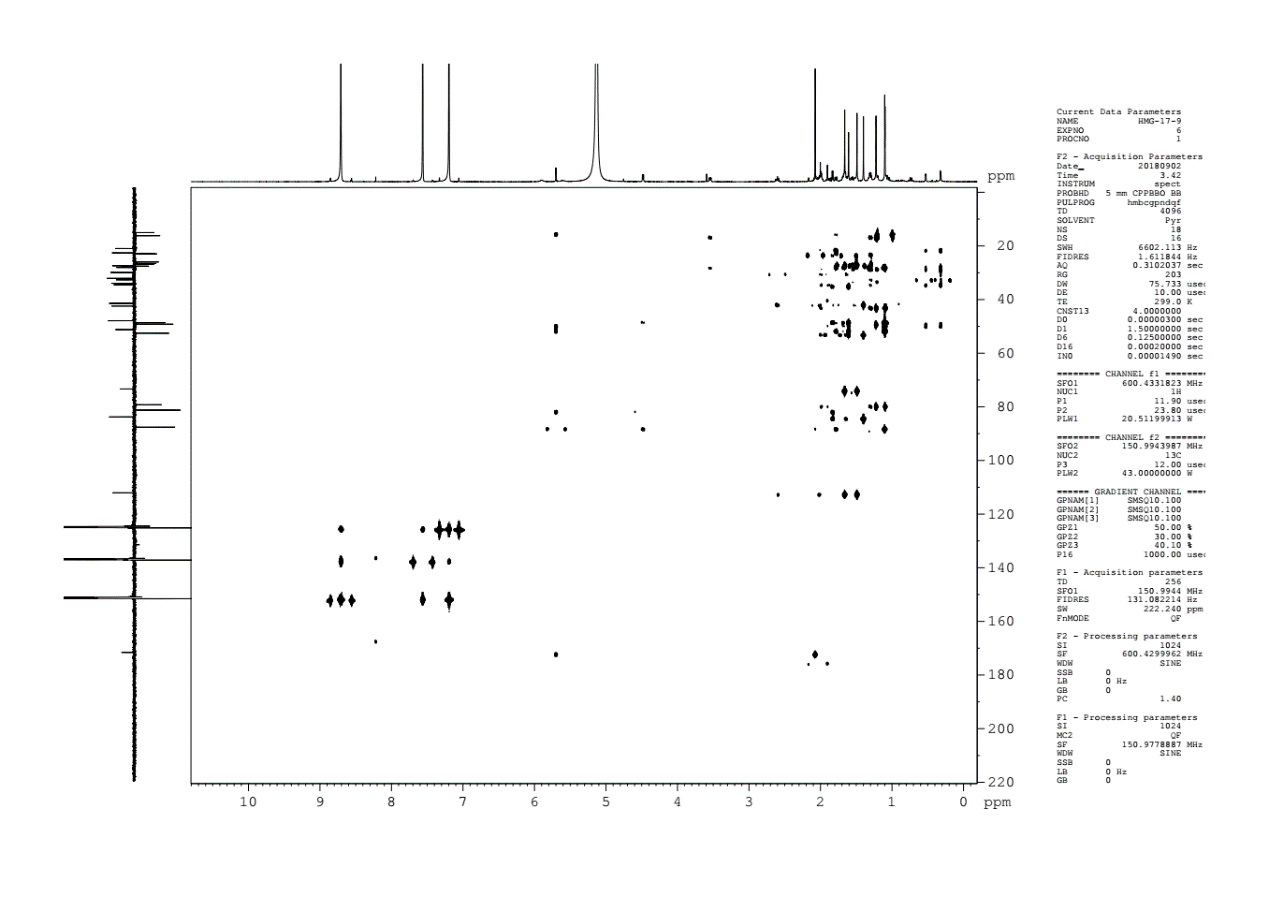


**Figure S14** HMBC (C5D5N) spectrum of **2**


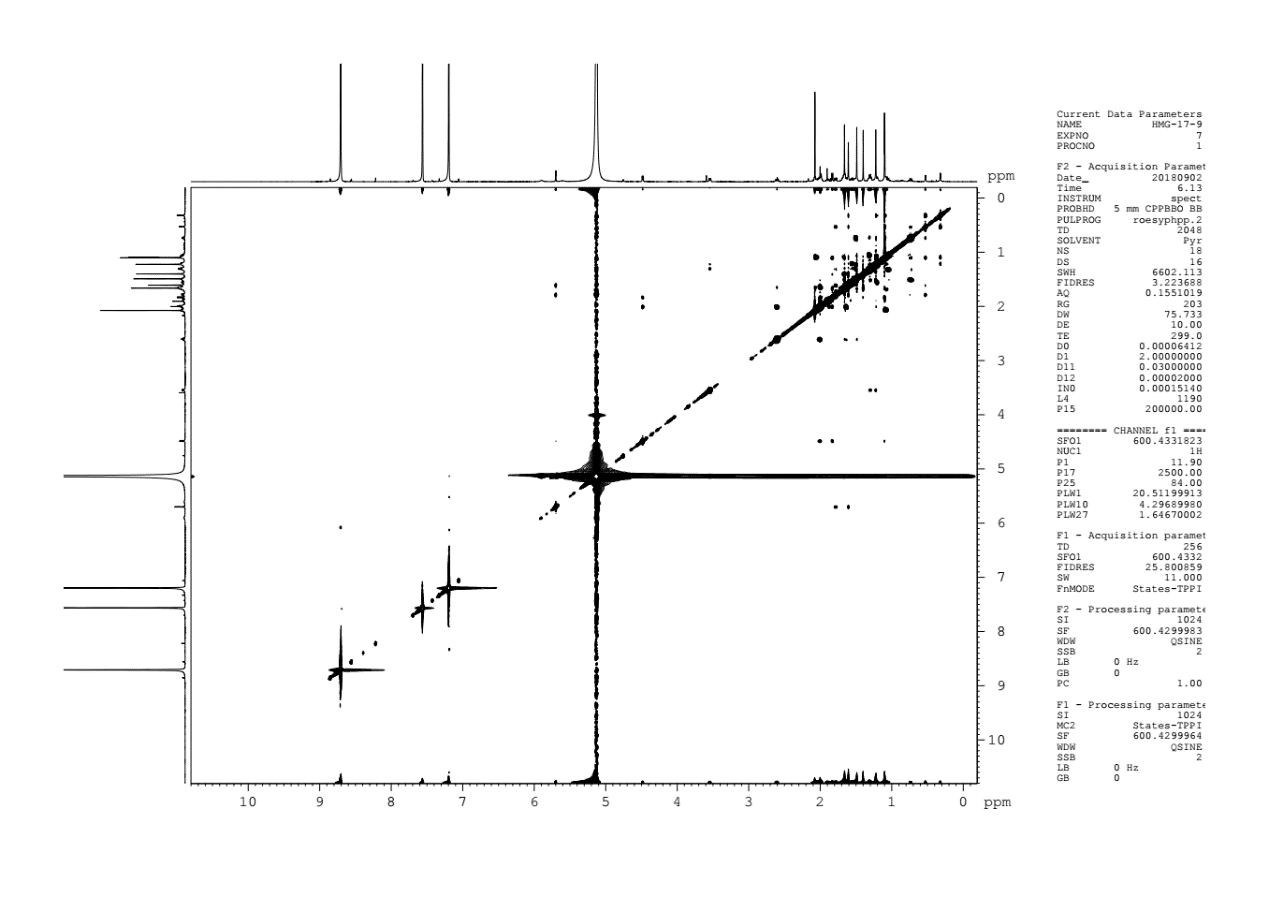


**Figure S15** NOESY (C5D5N) spectrum of **2**


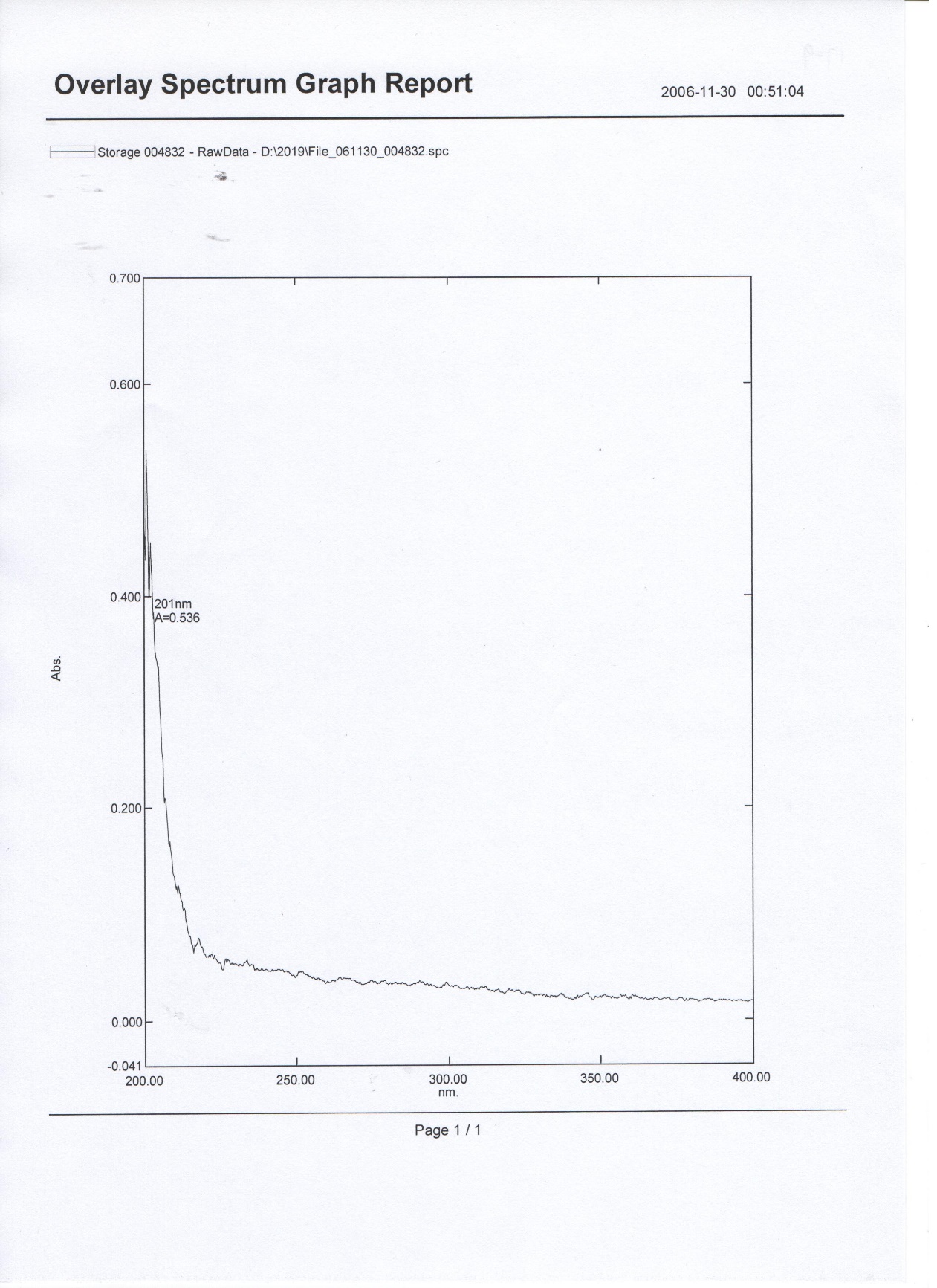


**Figure S16** UV spectrum of **2**


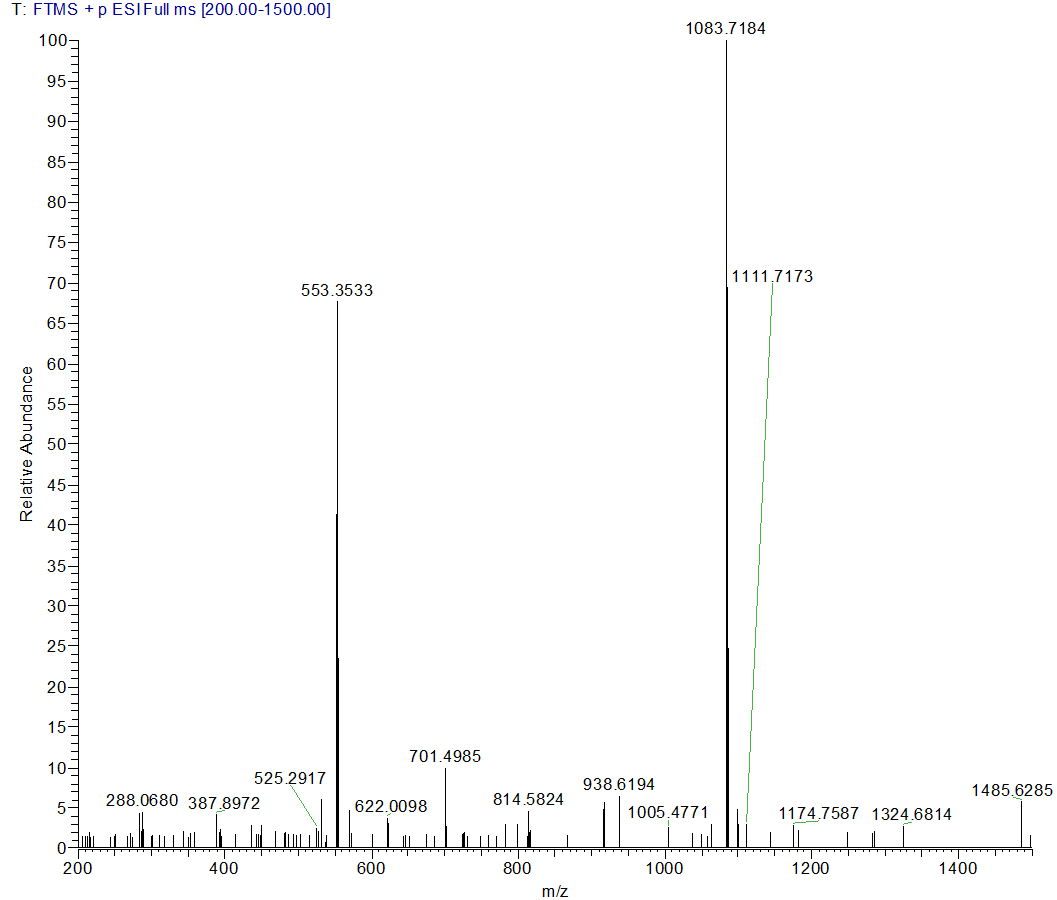


**Figure S17** HRESIMS spectrum of **2**


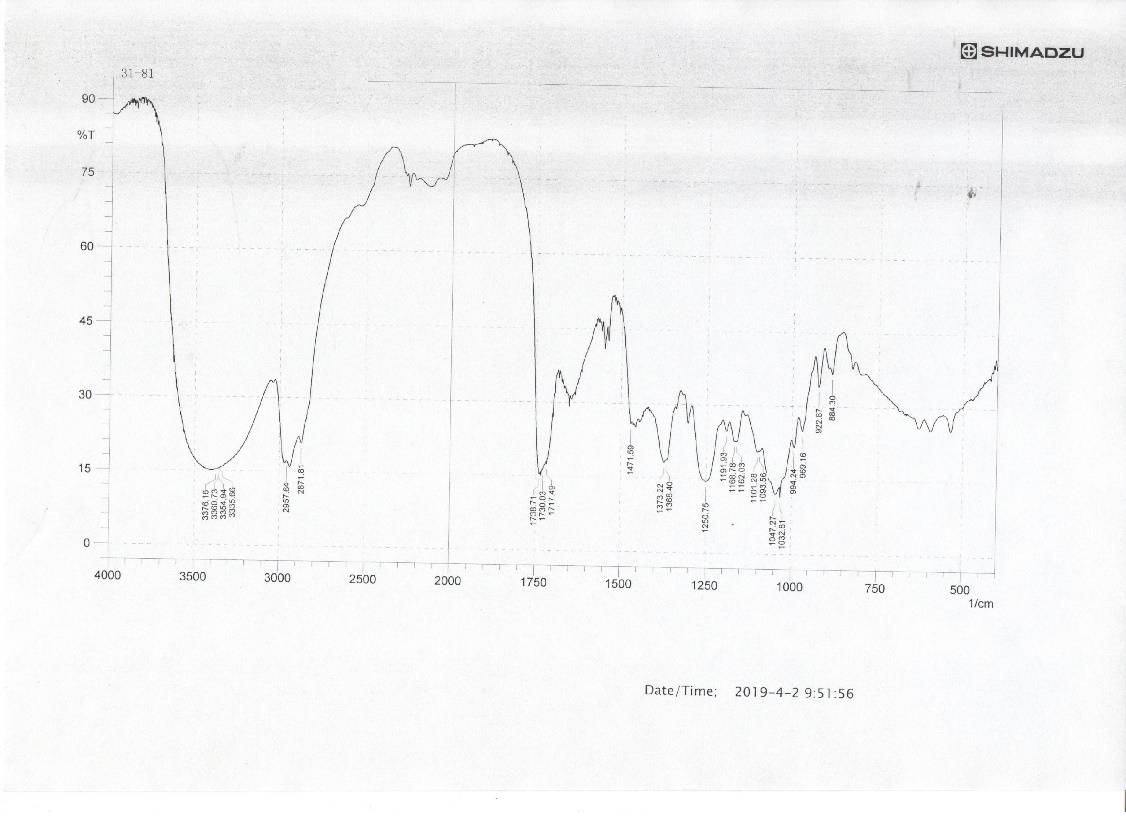


**Figure S18** IR spectrum of **2**


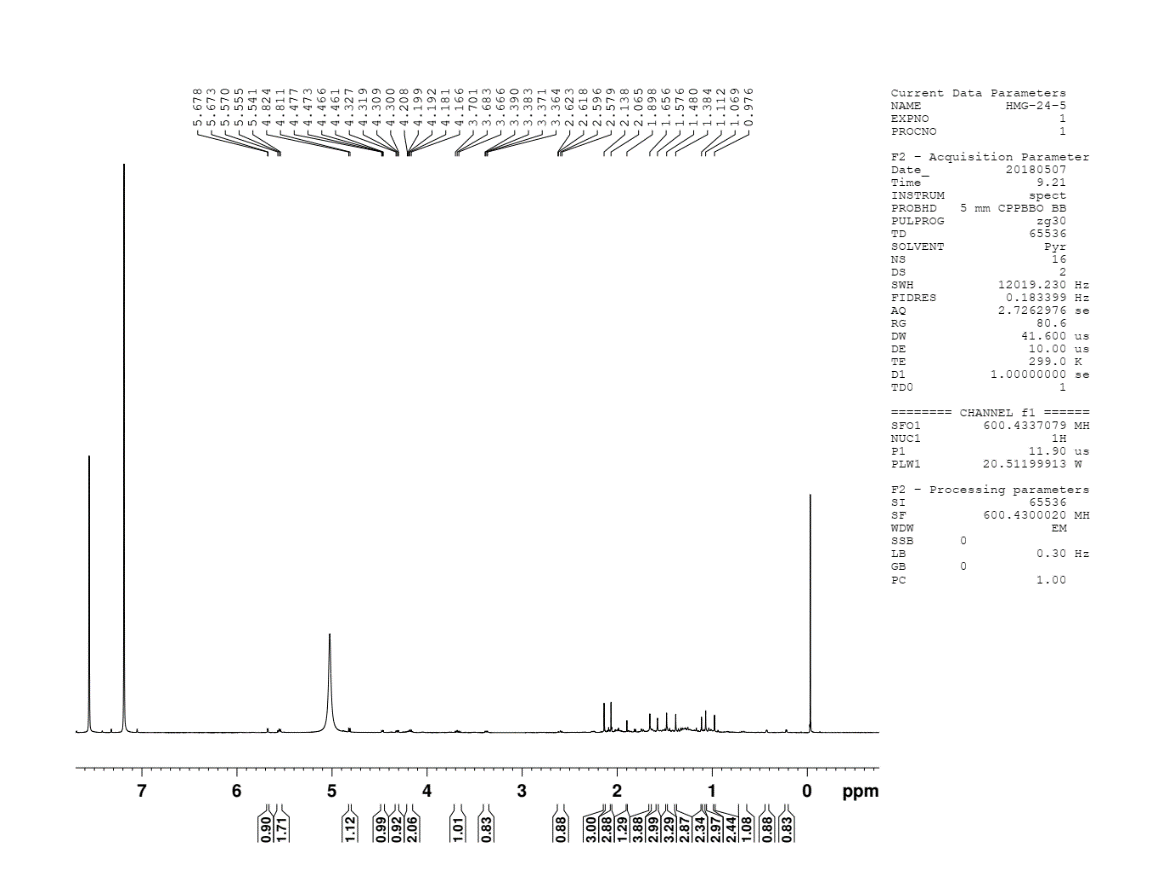


**Figure S19** ^1^H NMR (600 MHz, C5D5N) spectrum of **3**


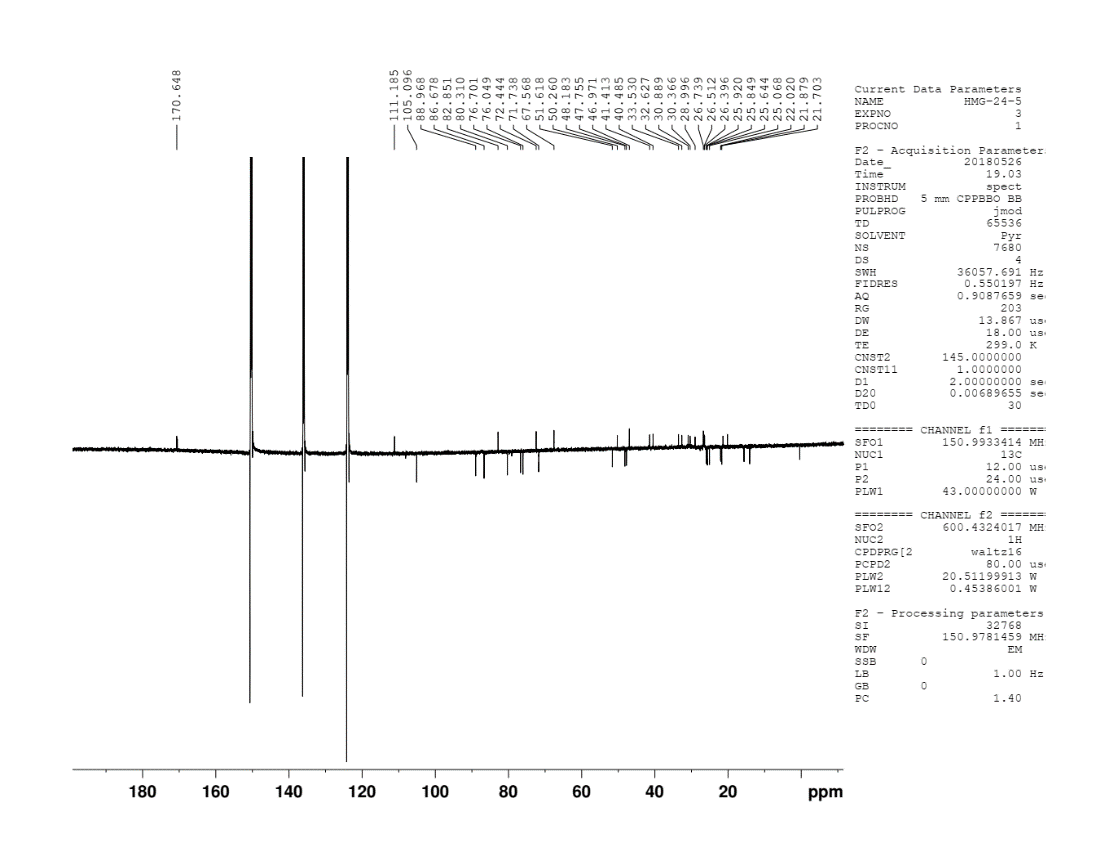


**Figure S20** ^13^C-APT (150 MHz, C5D5N) spectrum of **3**


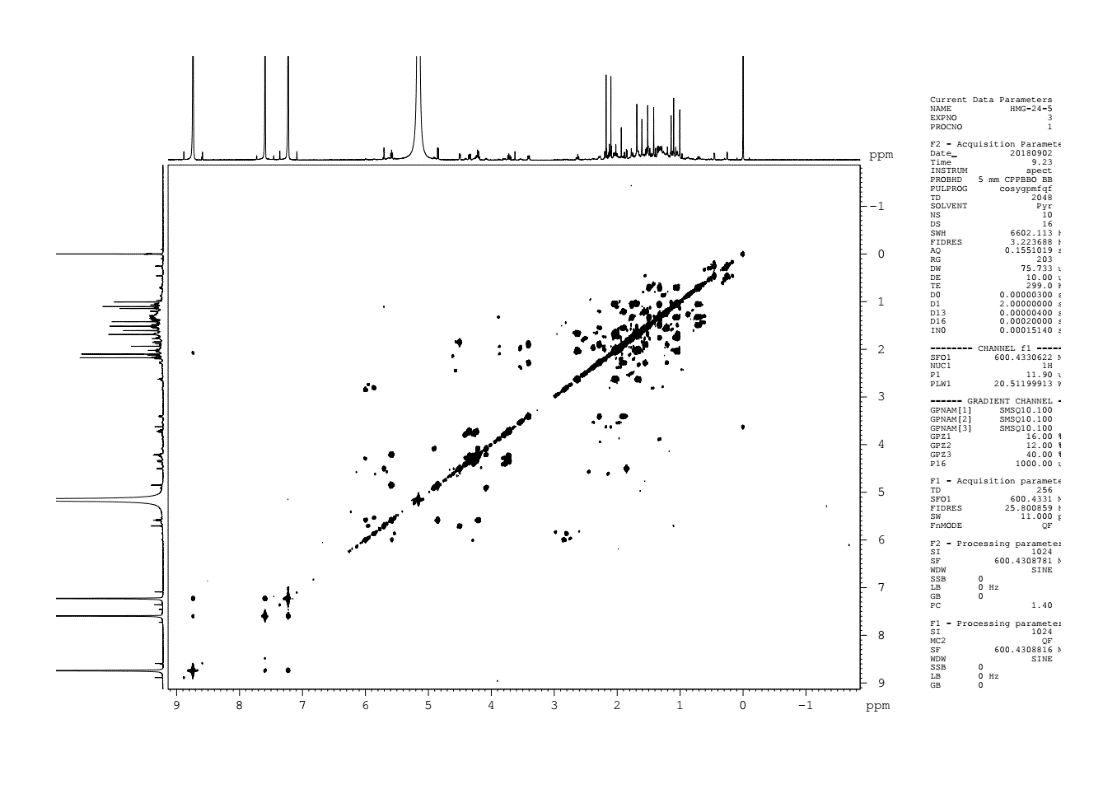


**Figure S21** ^1^H -^1^H COSY (C5D5N) spectrum of **3**


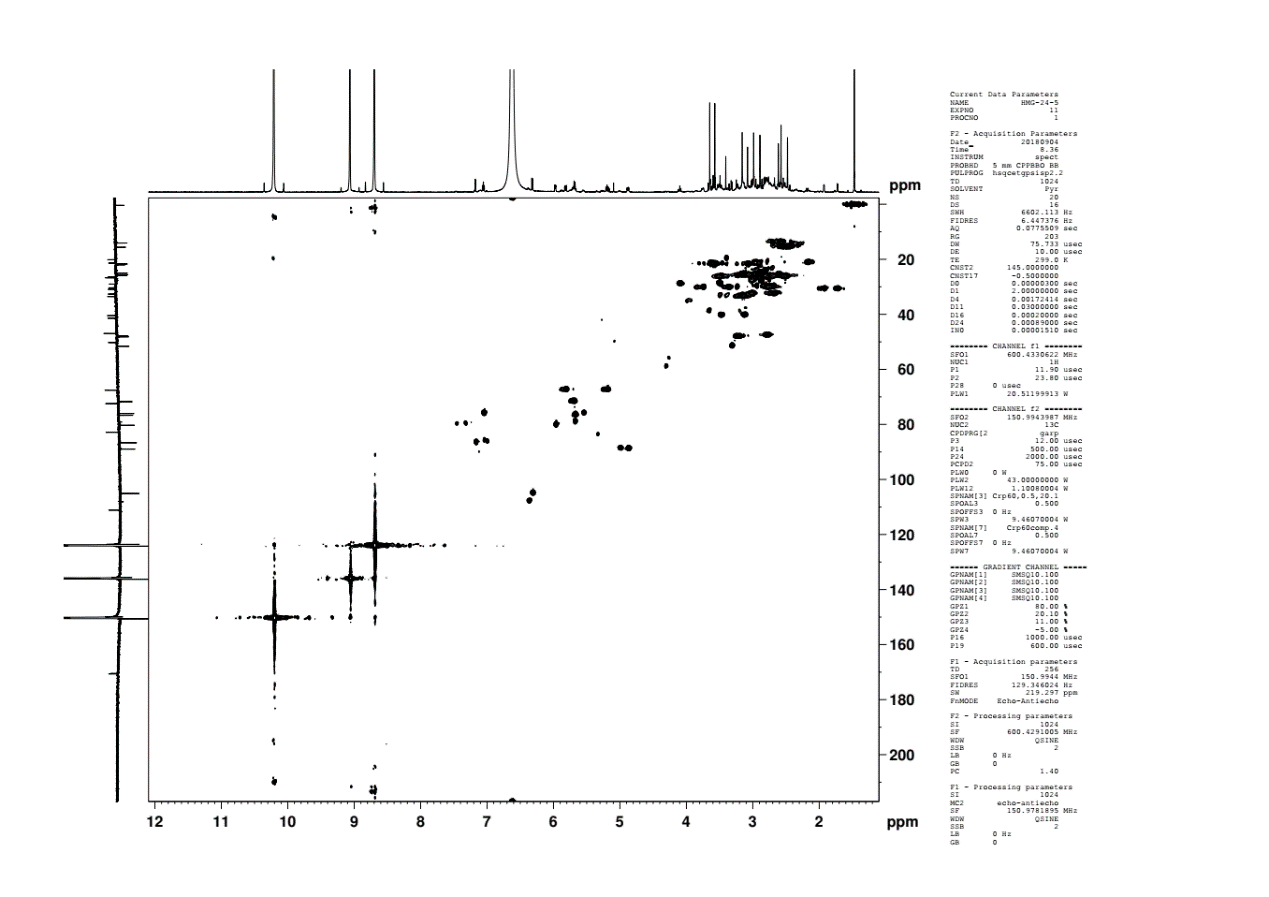


**Figure S22** HSQC (C5D5N) spectrum of **3**


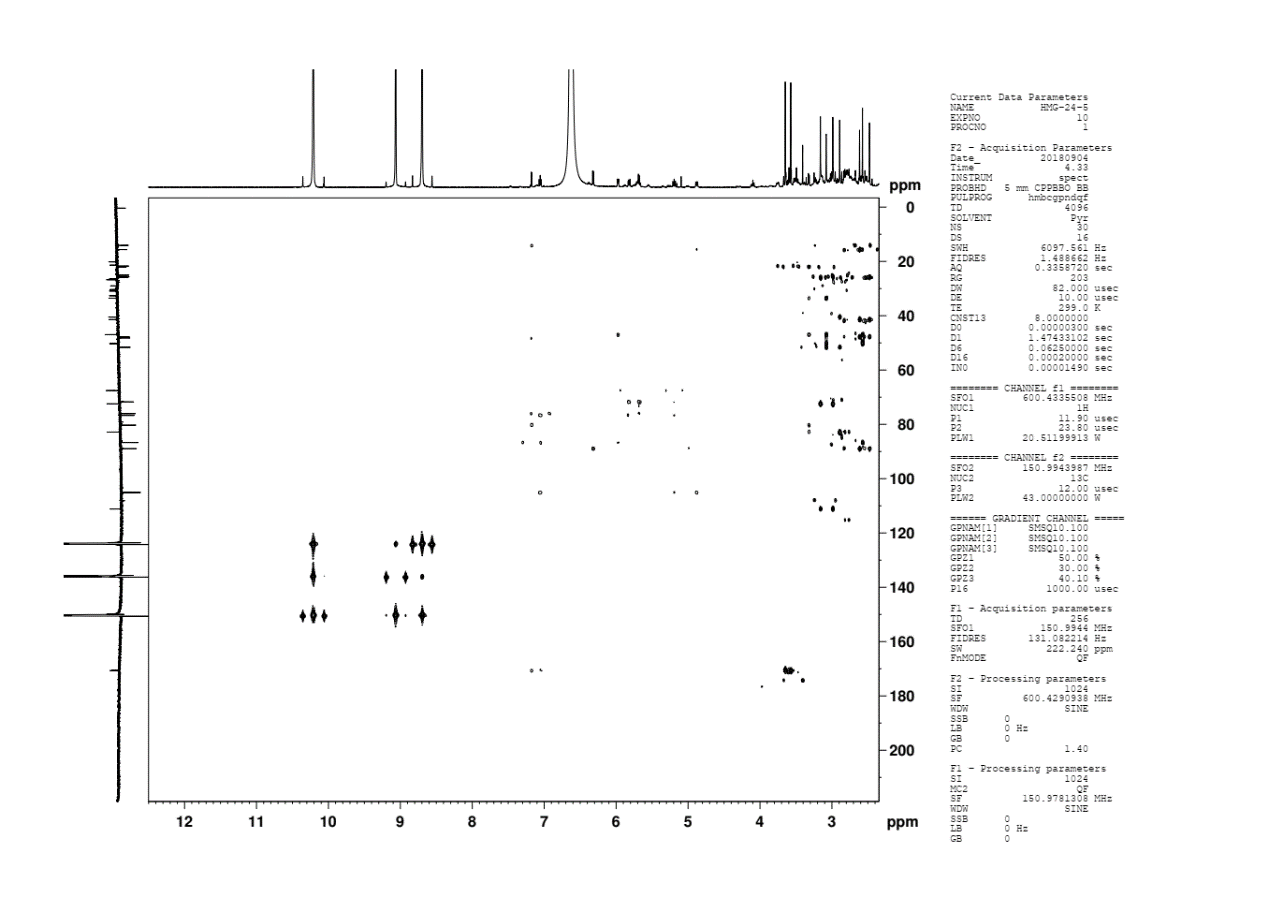


**Figure S23** HMBC (C5D5N) spectrum of **3**


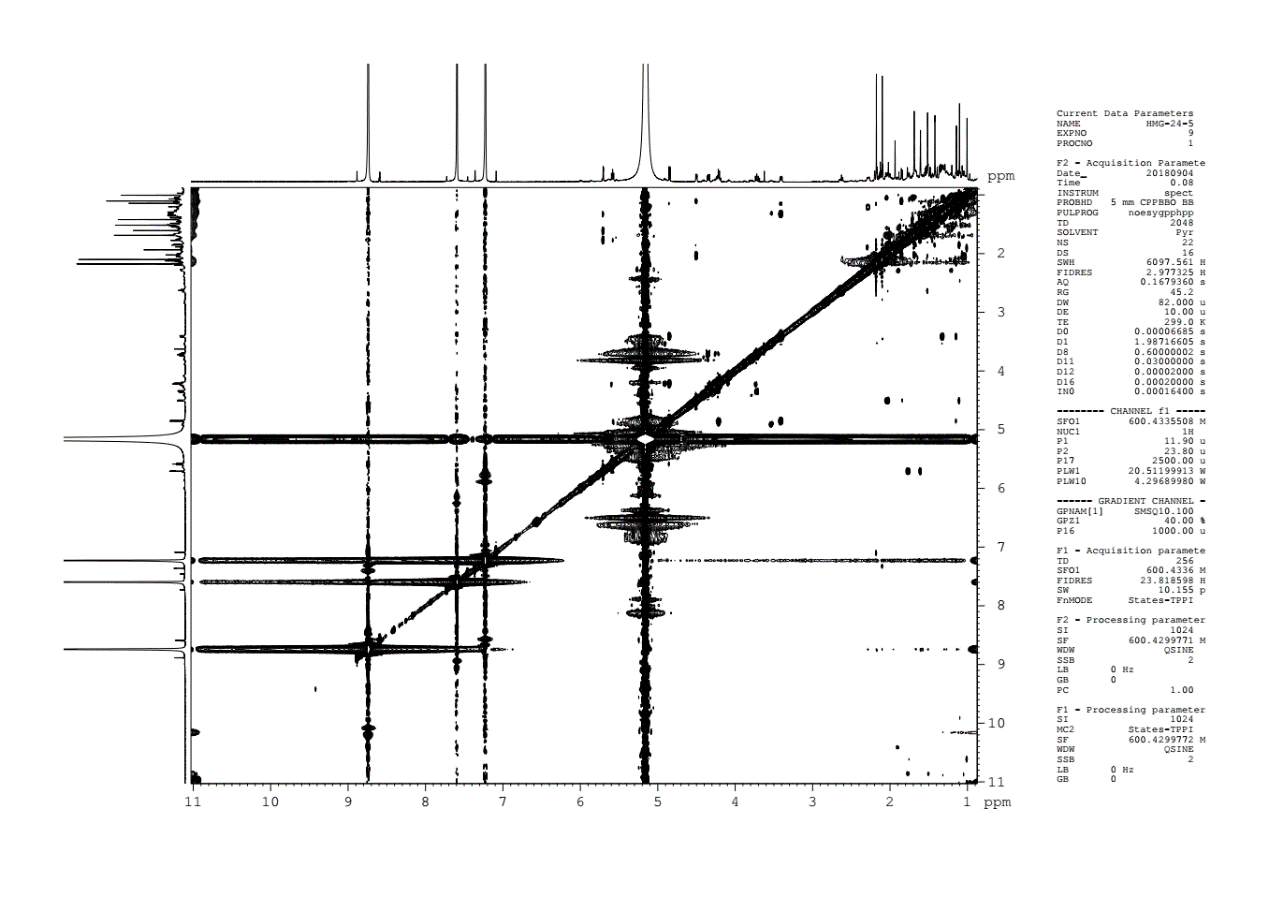


**Figure S24** NOESY (C5D5N) spectrum of **3**


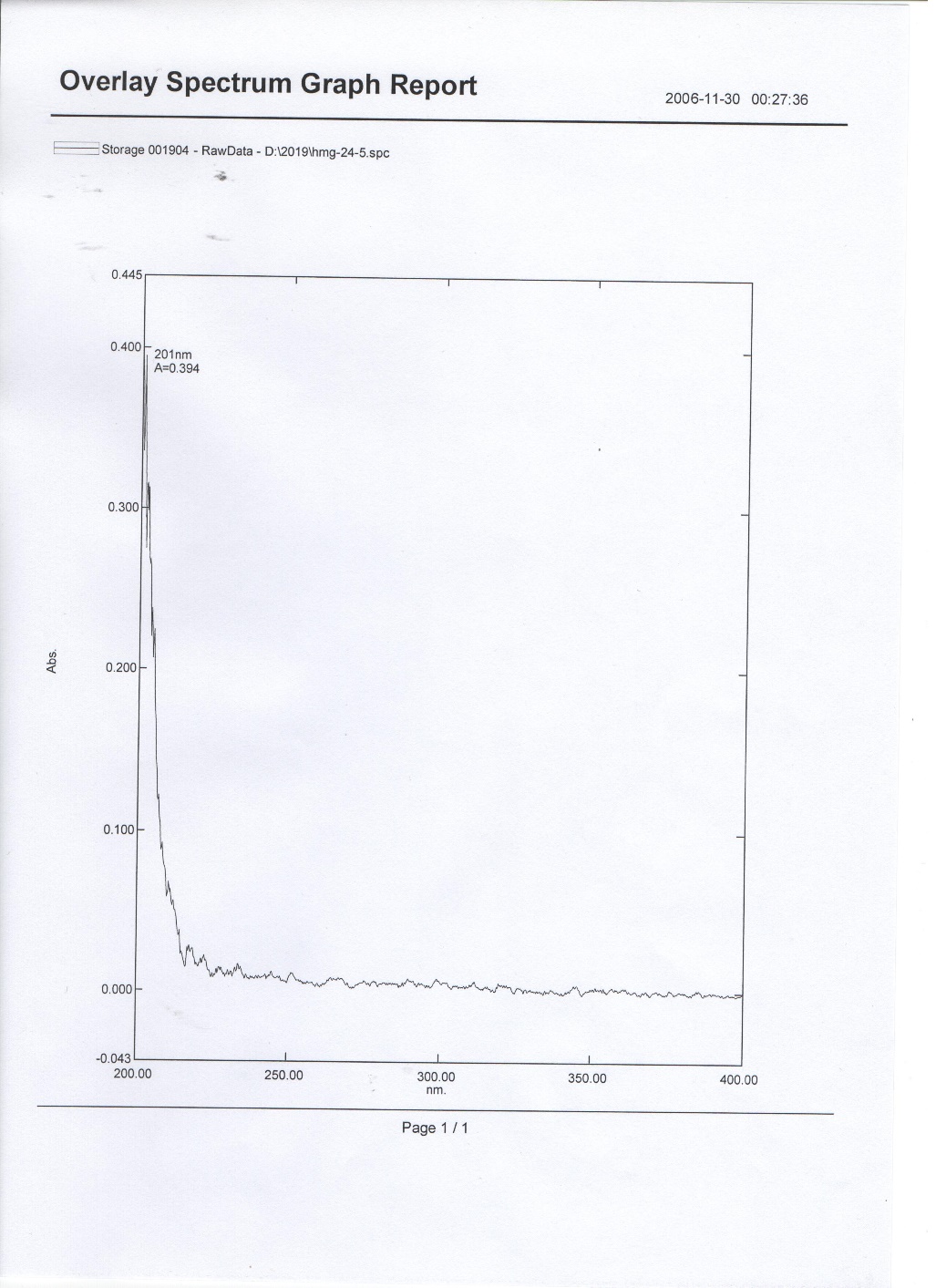


**Figure S25** UV spectrum of **3**


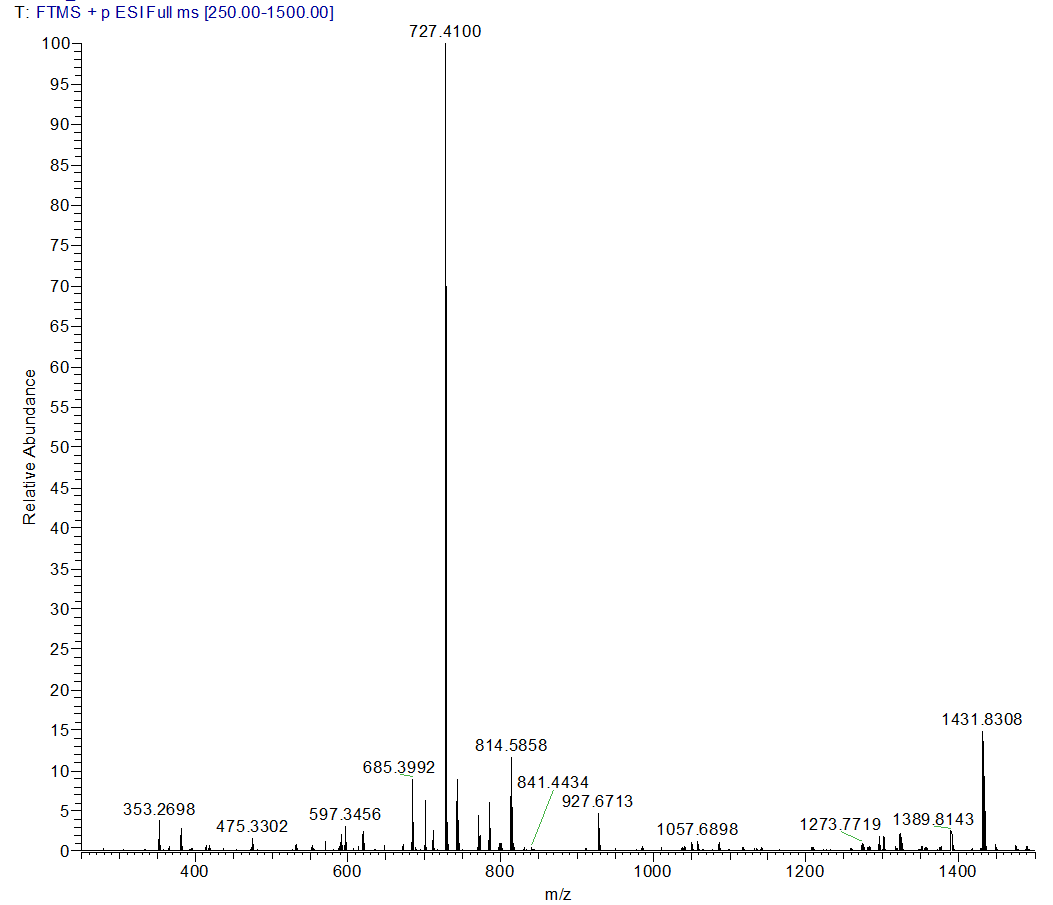


**Figure S26** HRESIMS spectrum of **3**


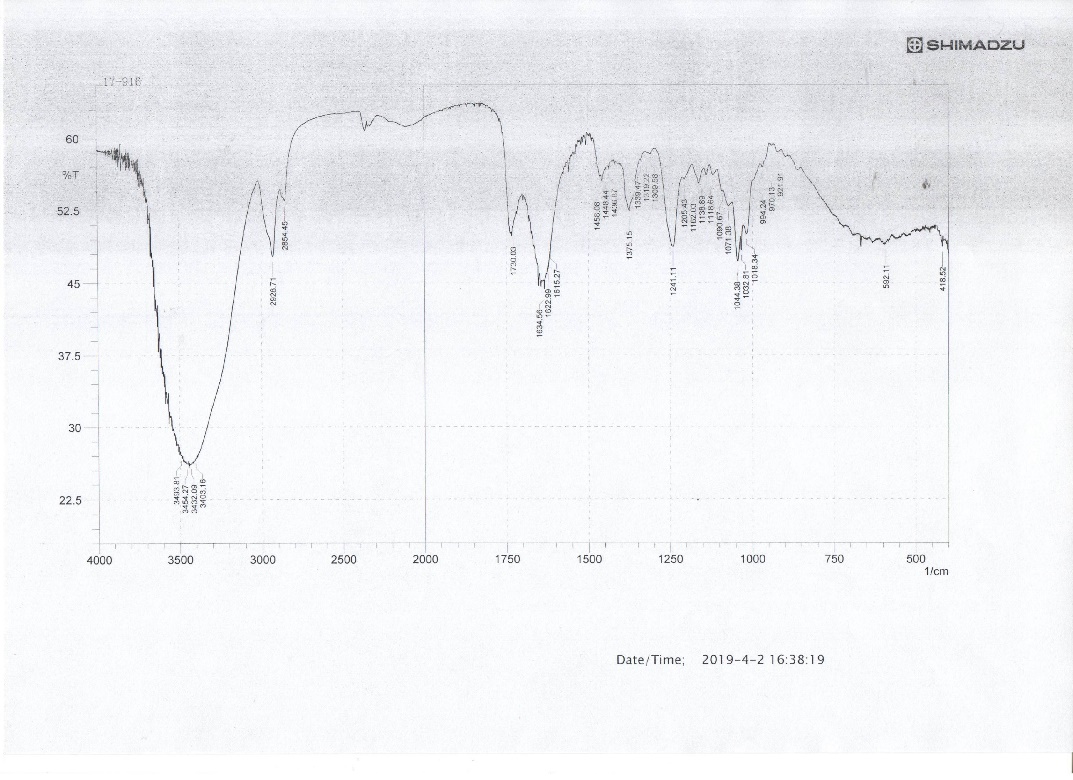


**Figure S27** IR spectrum of **3**


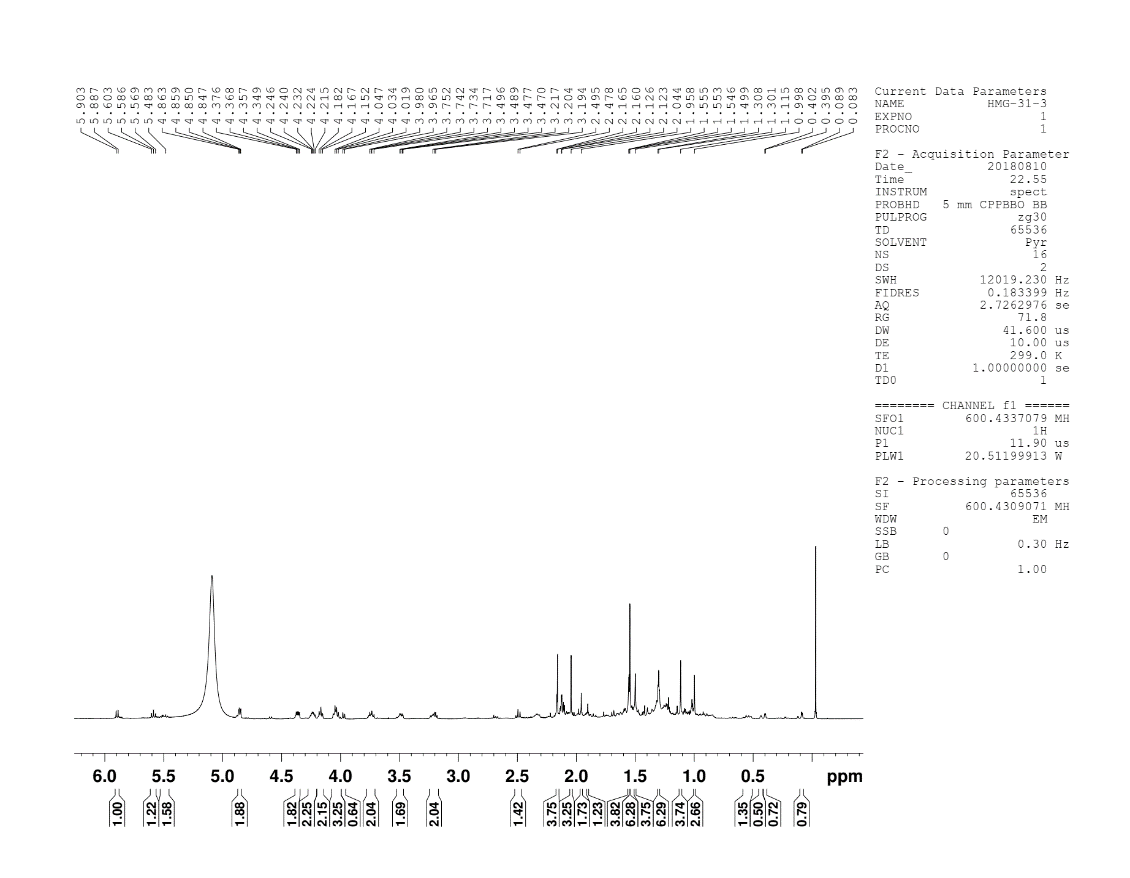


**Figure S28** ^1^H NMR (600 MHz, C5D5N) spectrum of **4**


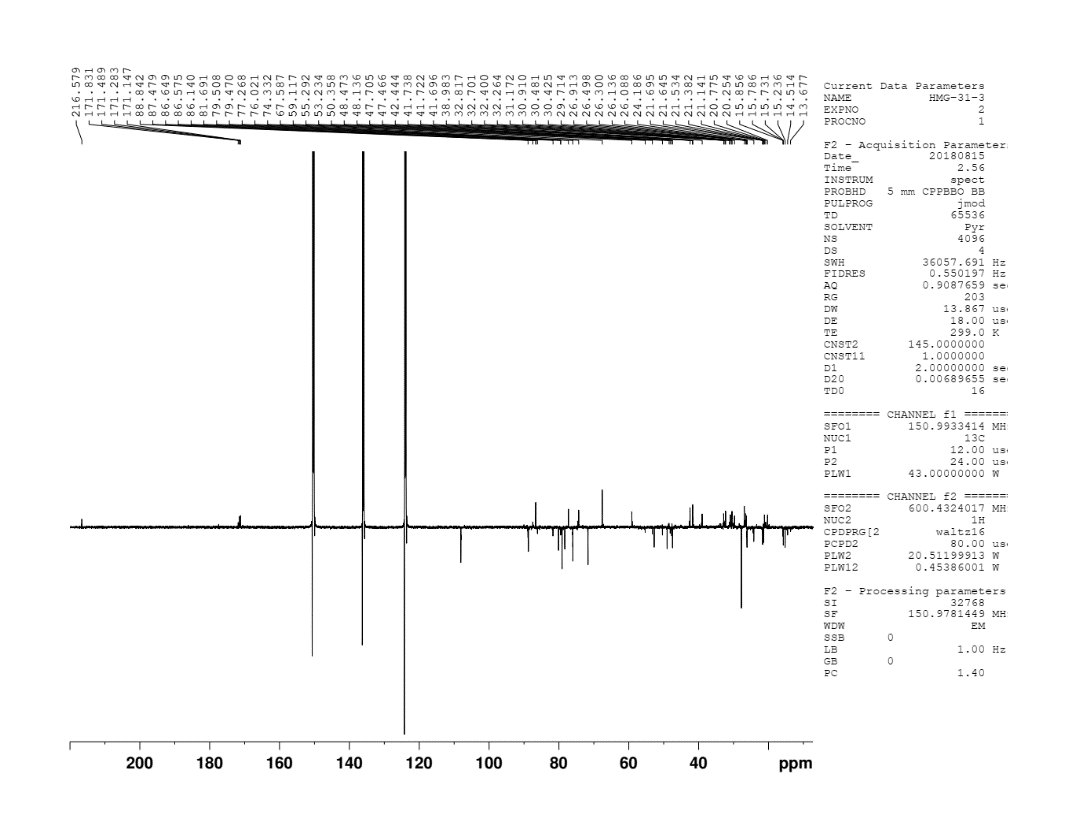


**Figure S29** ^13^C-APT (150 MHz, C5D5N) spectrum of **4**


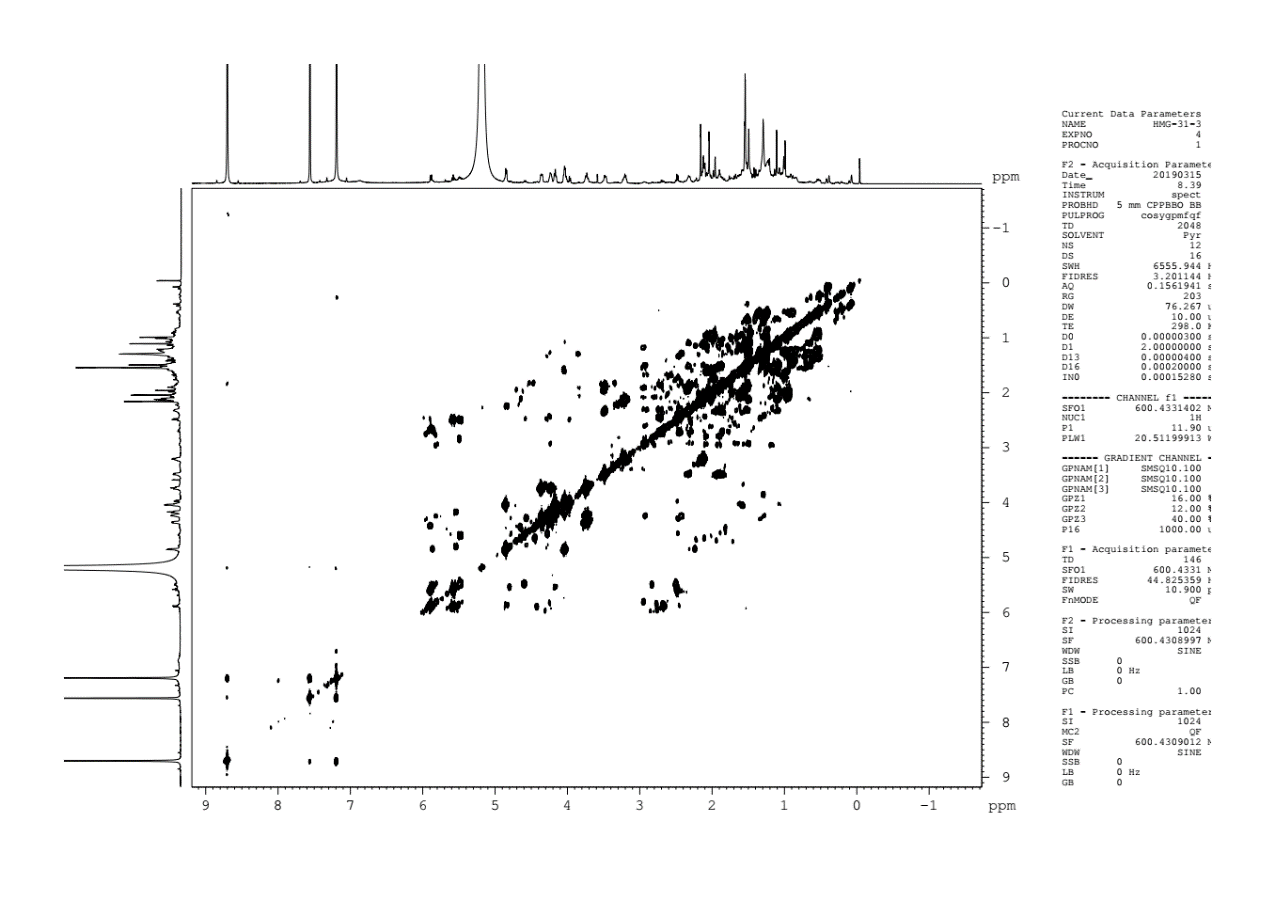


**Figure S30** ^1^H -^1^H COSY (C5D5N) spectrum of **4**


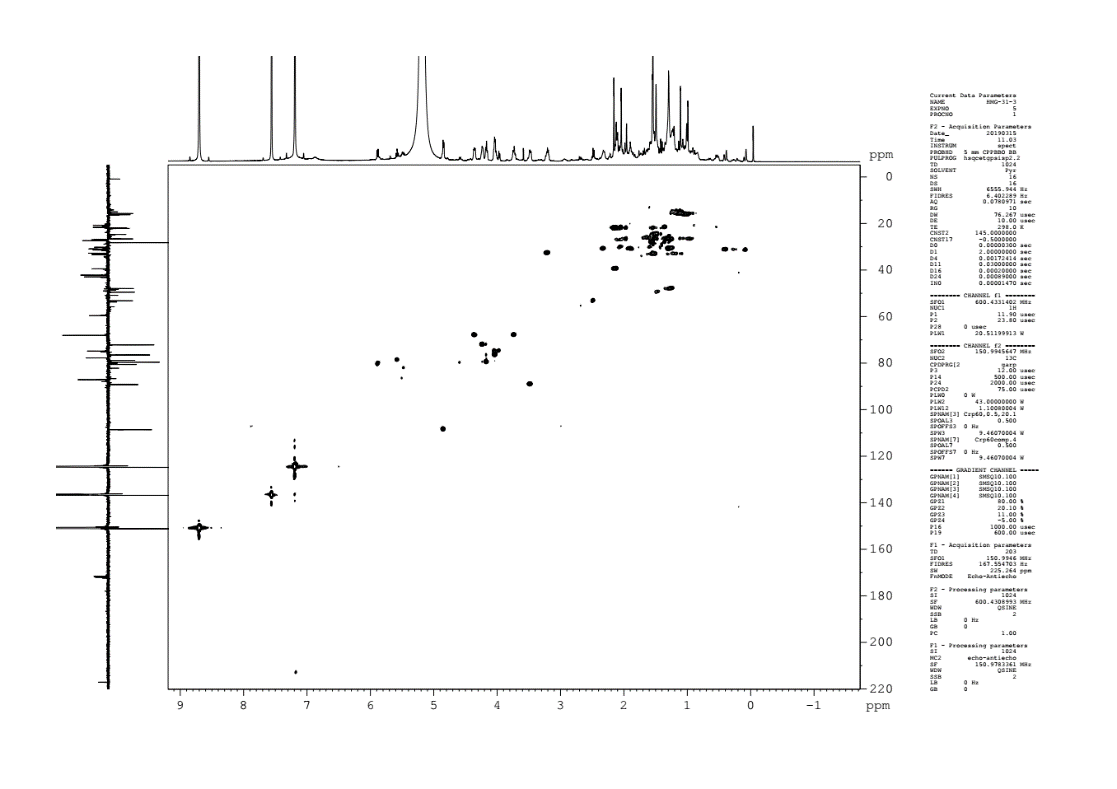


**Figure S31** HSQC (C5D5N) spectrum of **4**


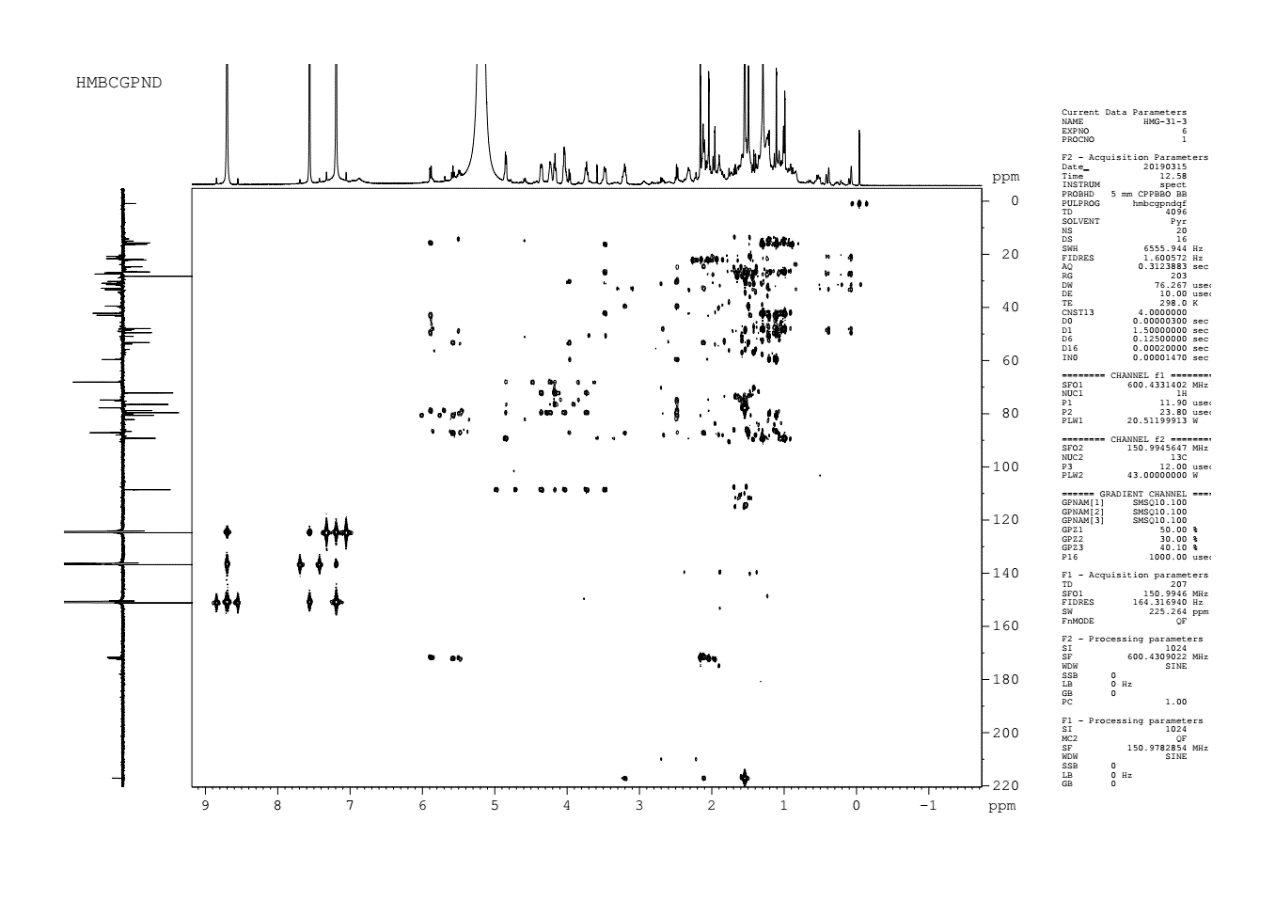


**Figure S32** HMBC (C5D5N) spectrum of **4**


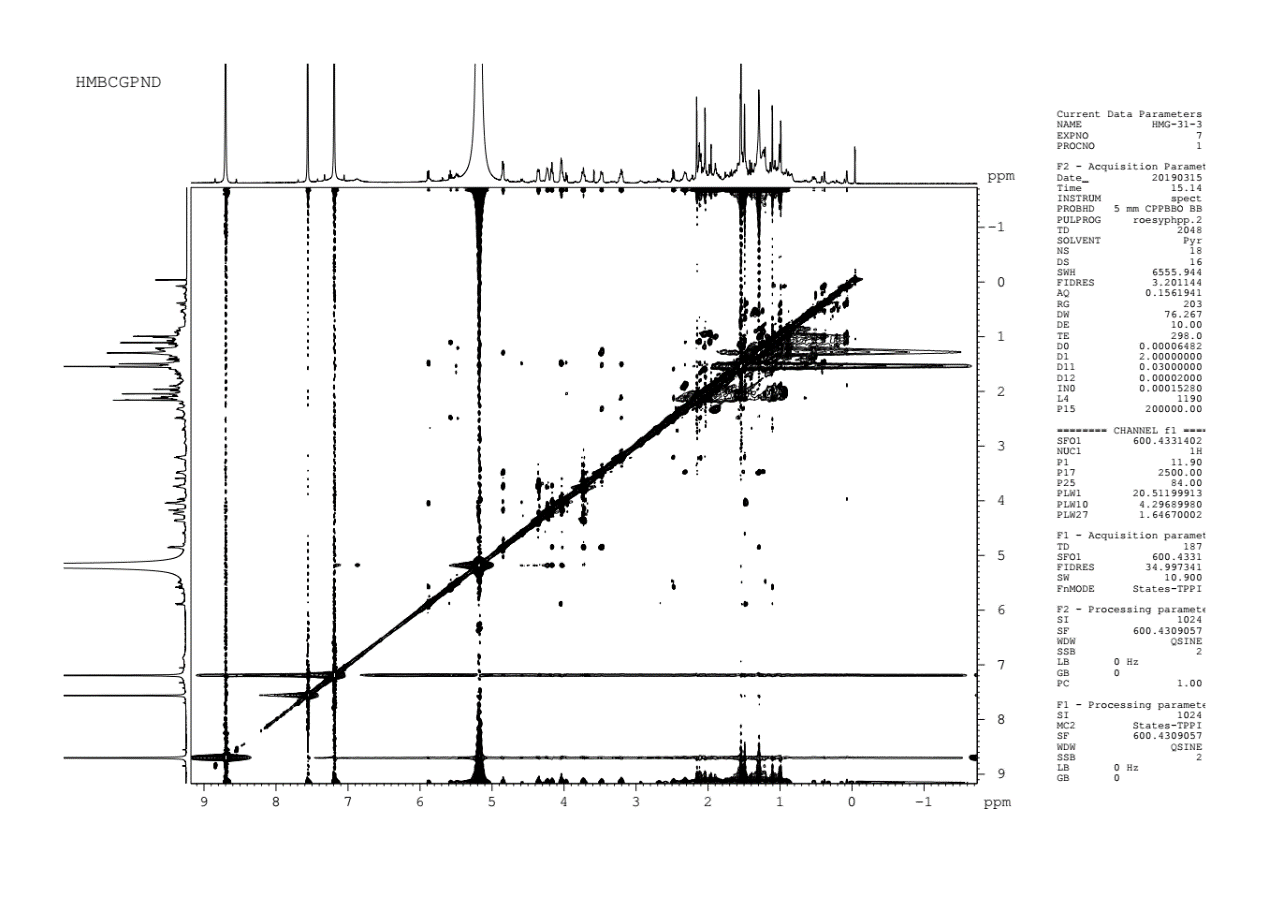


**Figure S33** NOSEY (C5D5N) spectrum of **4**


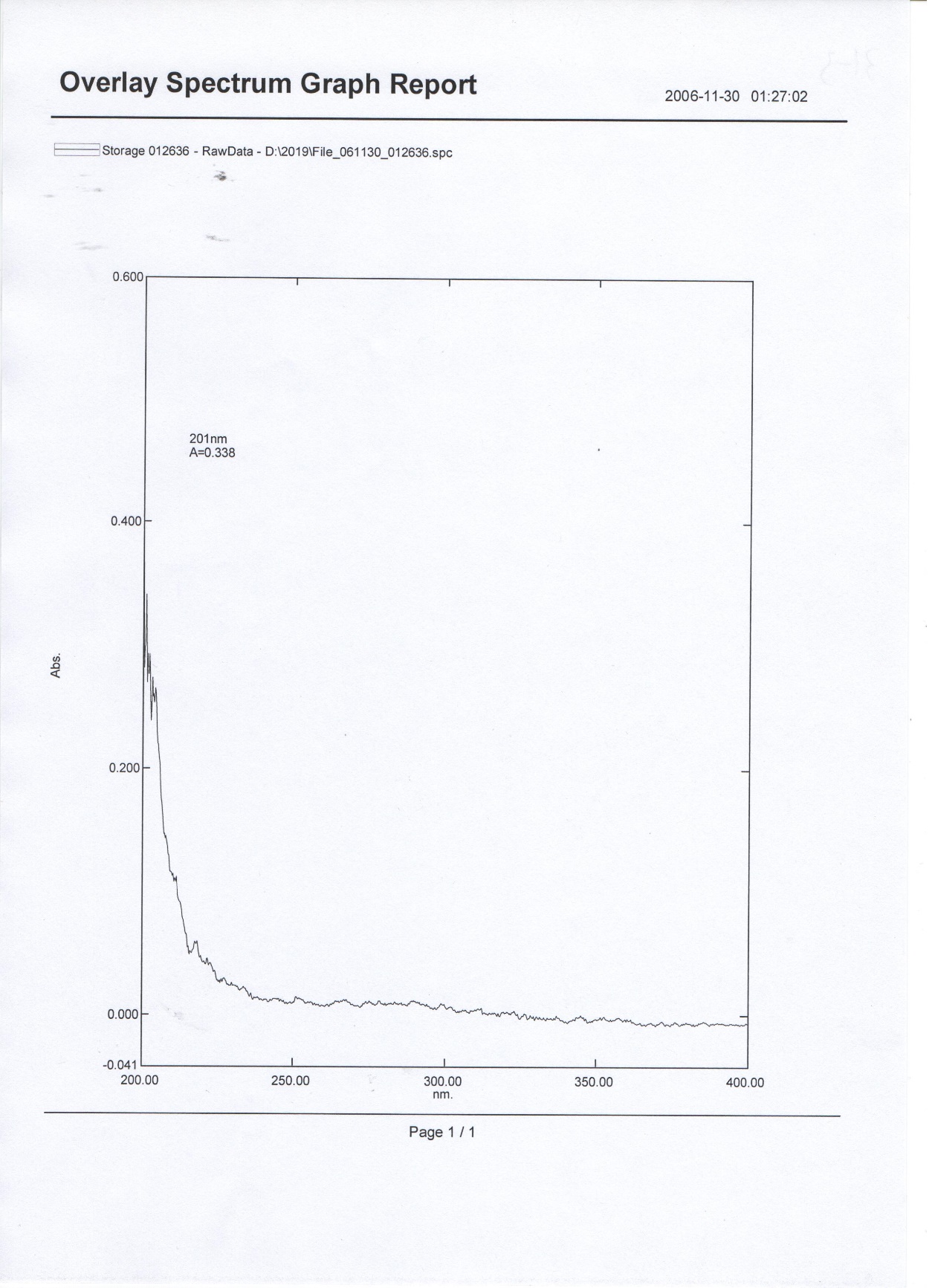


**Figure S34** UV spectrum of **4**


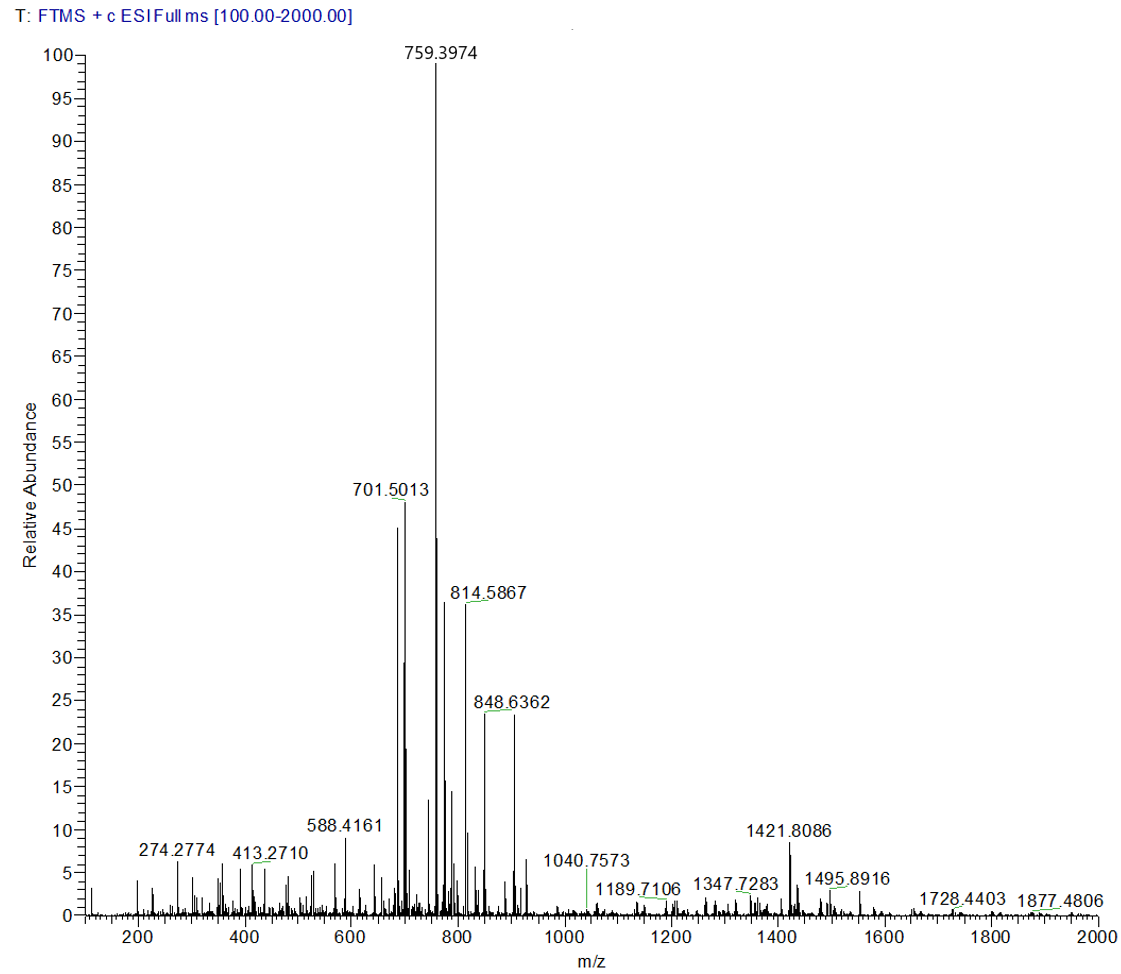
**Figure S35** **Figure S35** HRESIMS spectrum of **4**


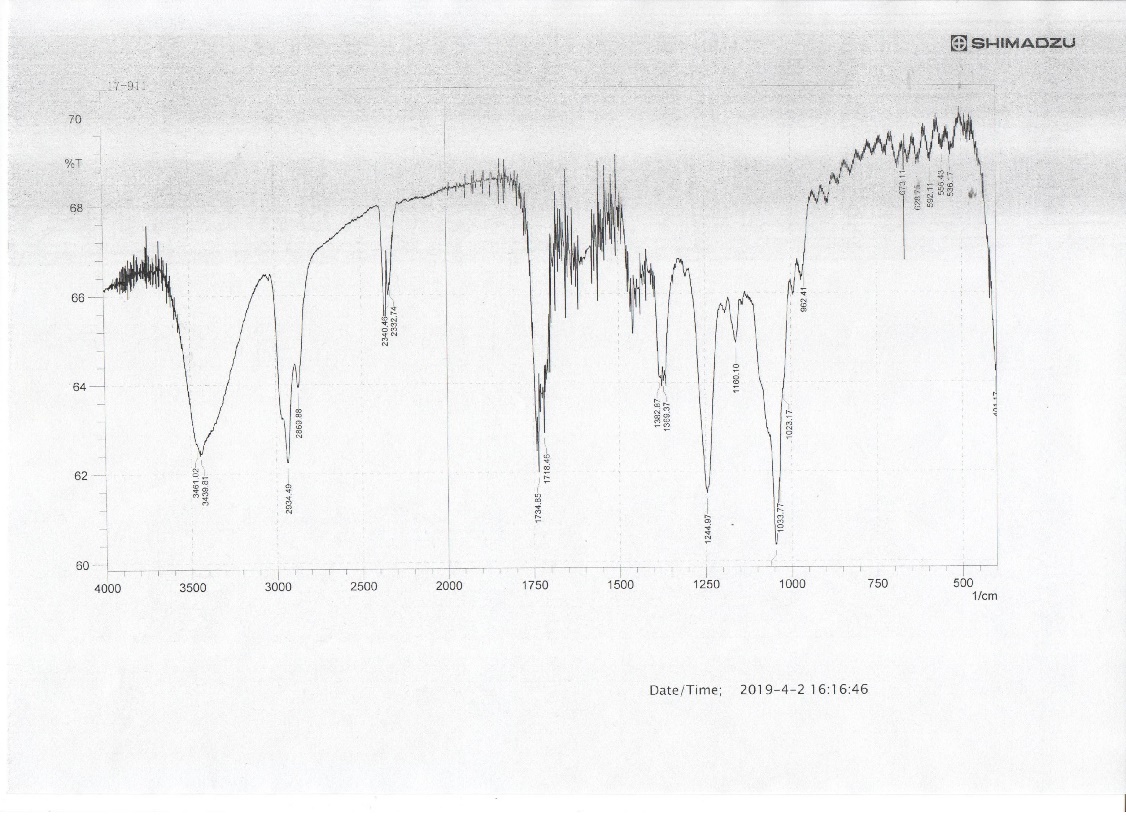


**Figure S36** IR spectrum of **4**


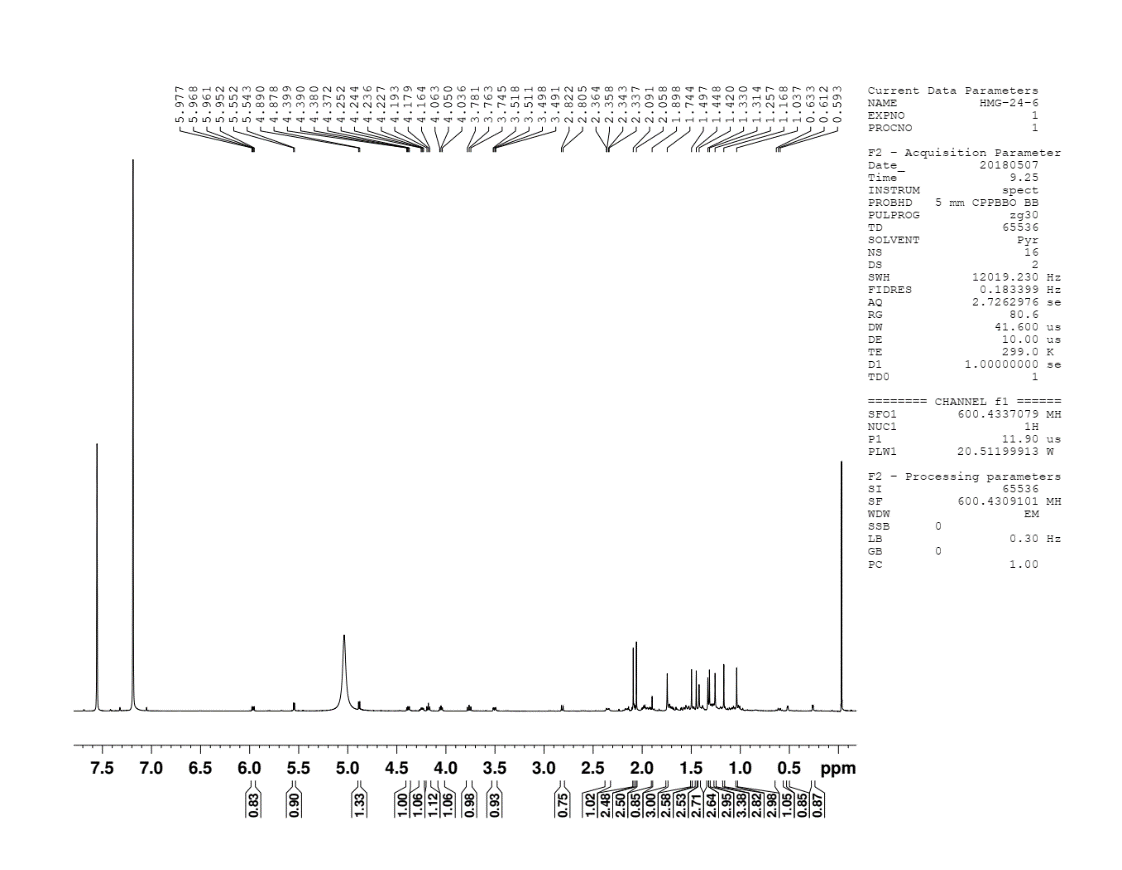


**Figure S37** ^1^H NMR (600 MHz, C5D5N) spectrum of **5**


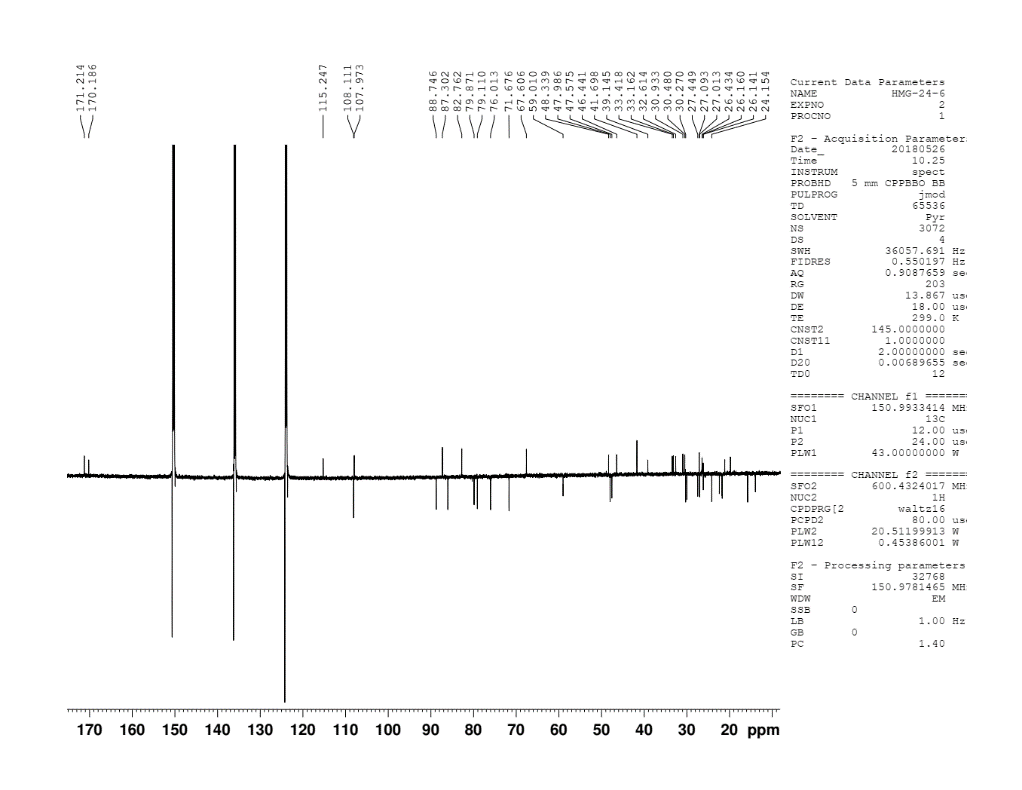


**Figure S38** ^13^C-APT (150 MHz, C5D5N) spectrum of **5**


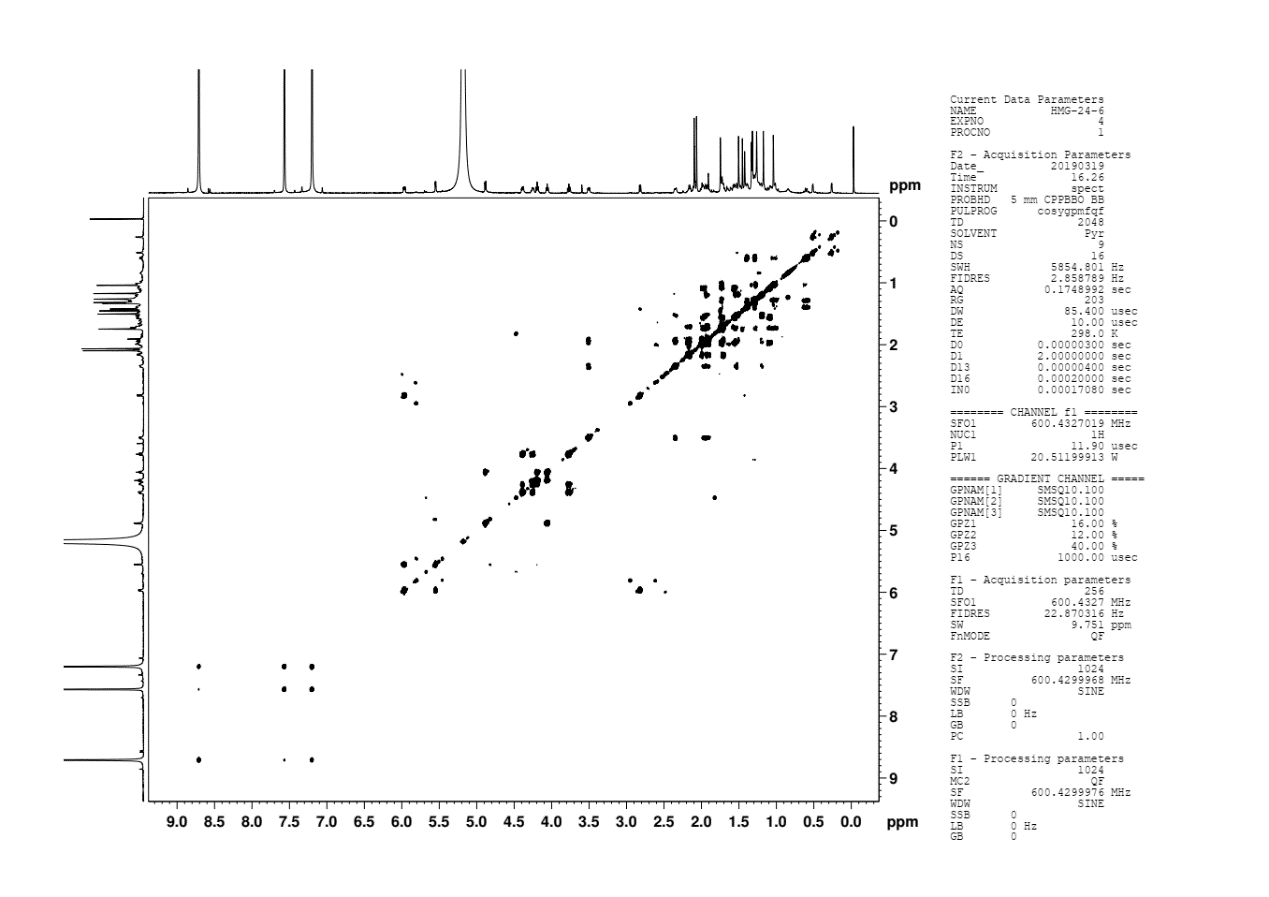


**Figure S39** ^1^H ^1^H COSY (C5D5N) spectrum of **5**


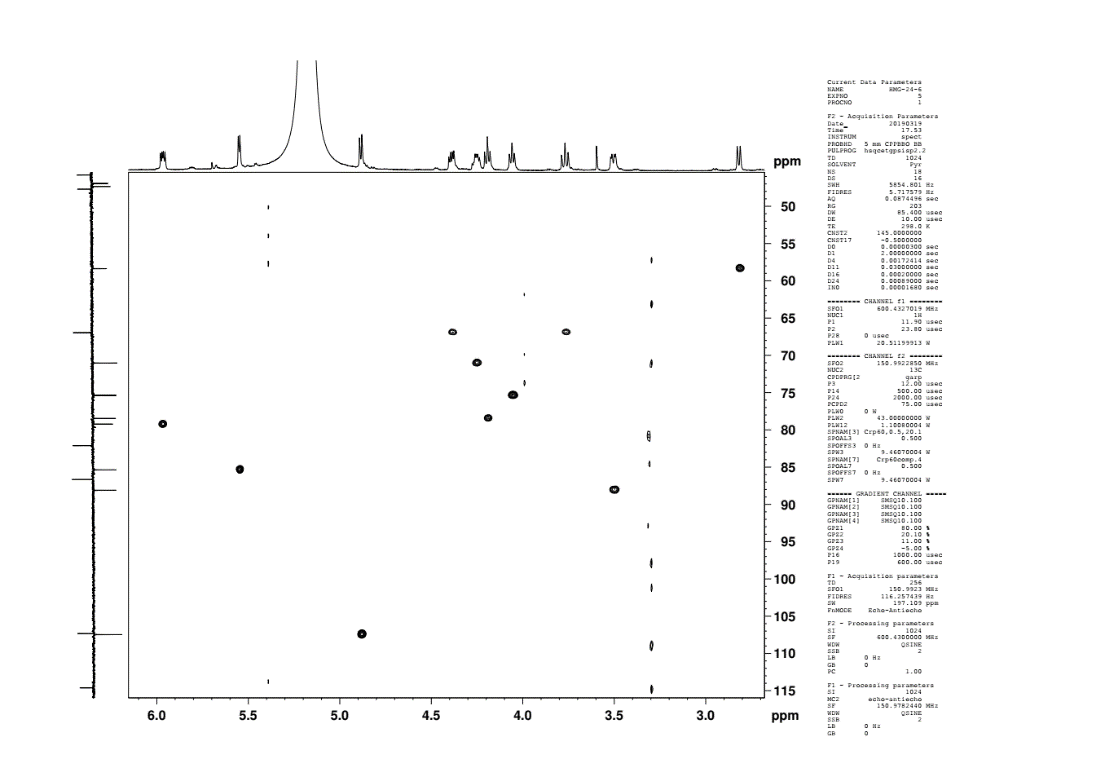


**Figure S40** HSQC (C5D5N) spectrum of **5**


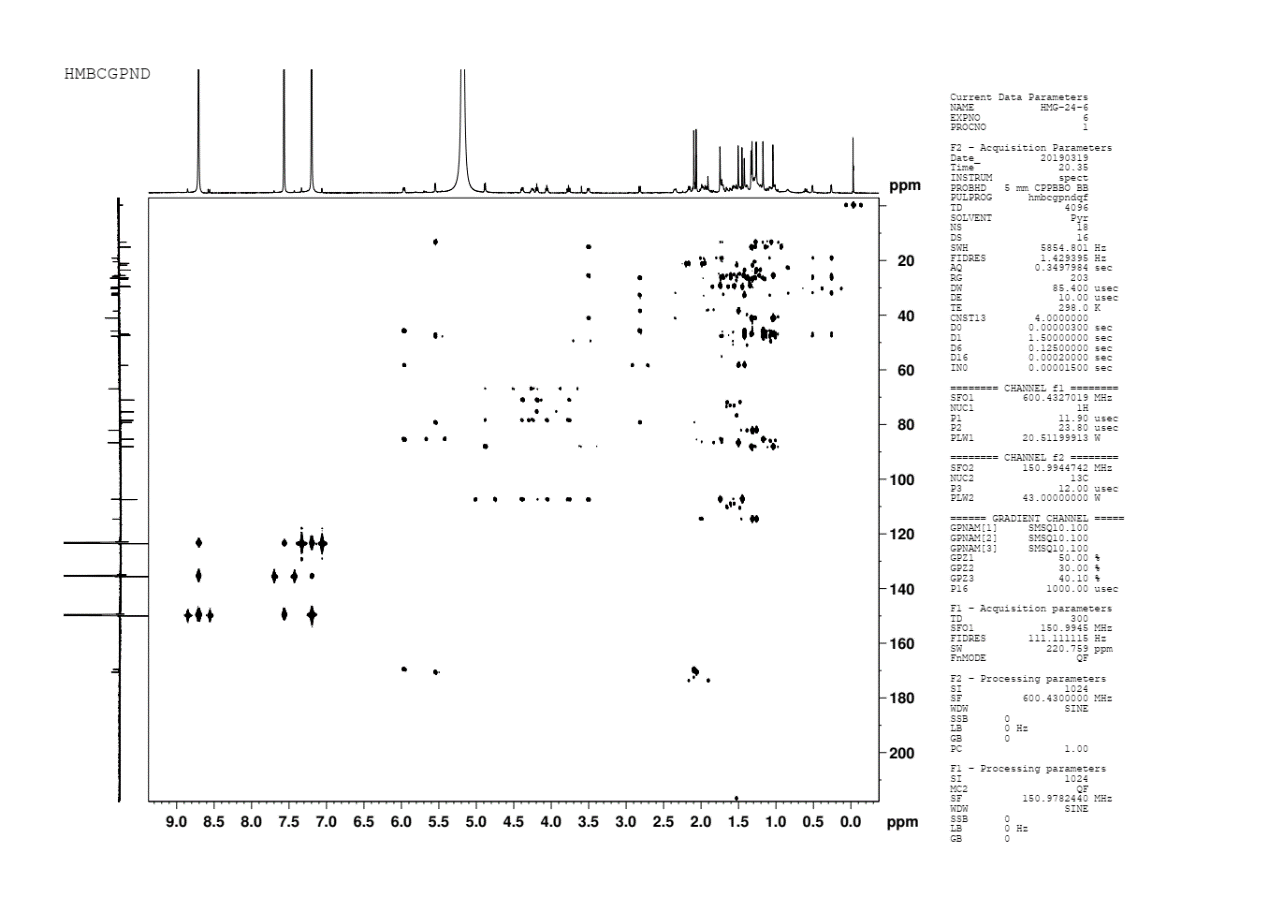


**Figure S41** HMBC (C5D5N) spectrum of **5**


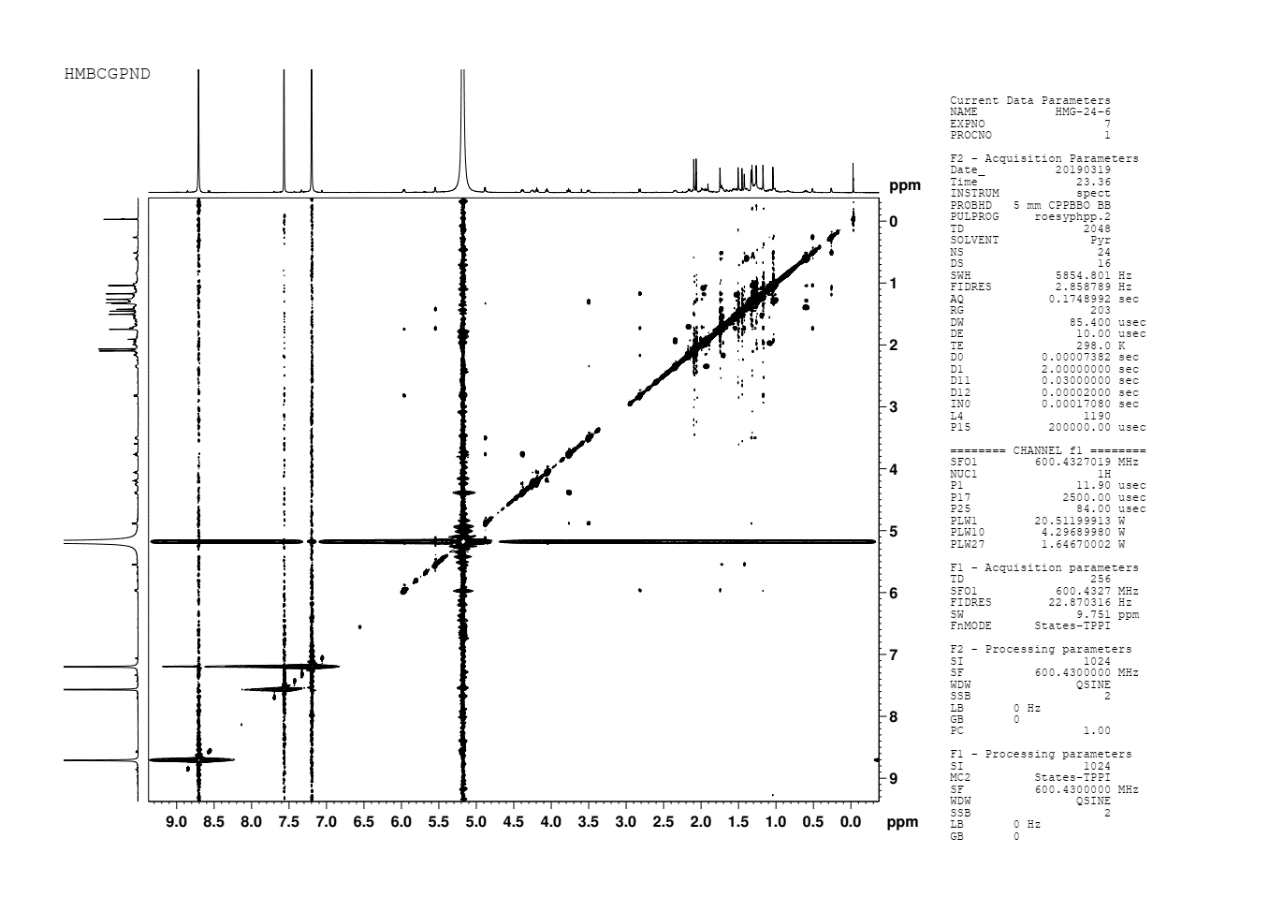


**Figure S42** NOSEY (C5D5N) spectrum of **5**


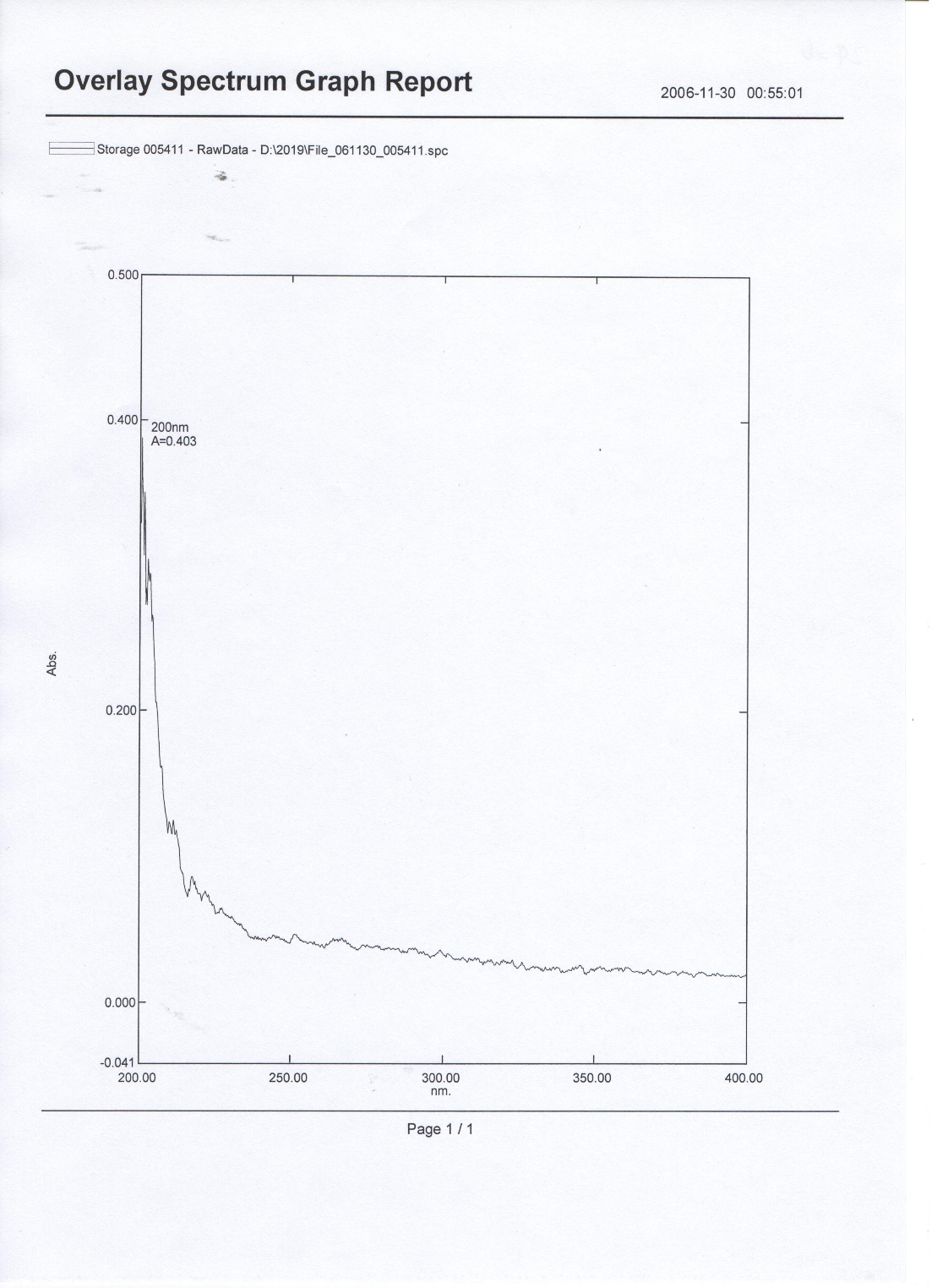


**Figure S43** UV spectrum of **5**


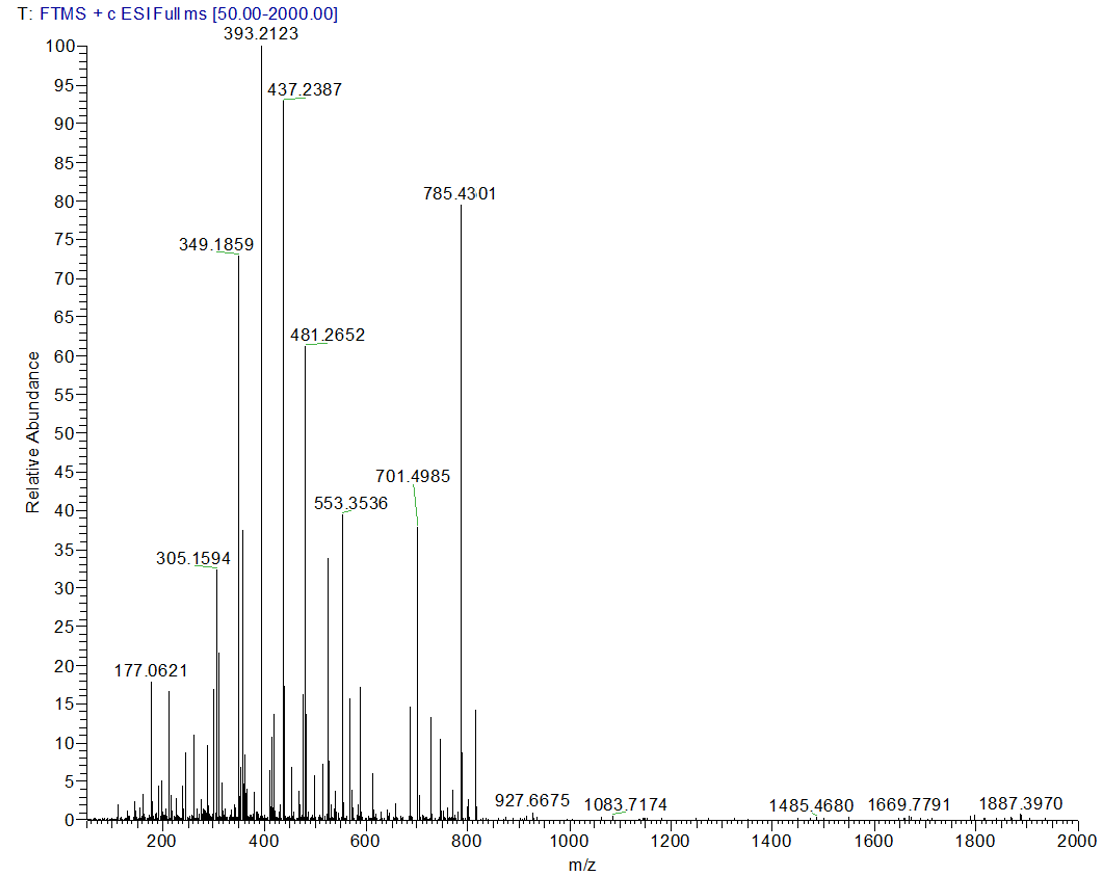


**Figure S44** HRESIMS spectrum of **5**


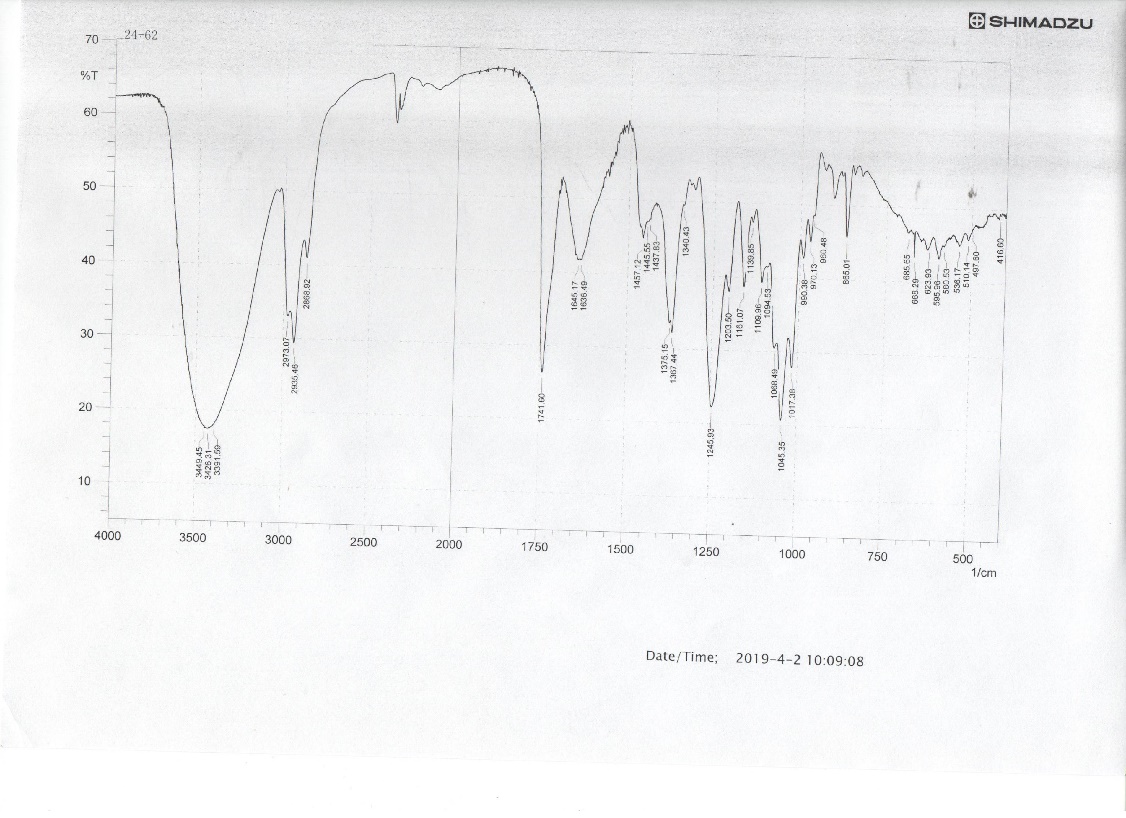


**Figure S45** IR spectrum of **5**


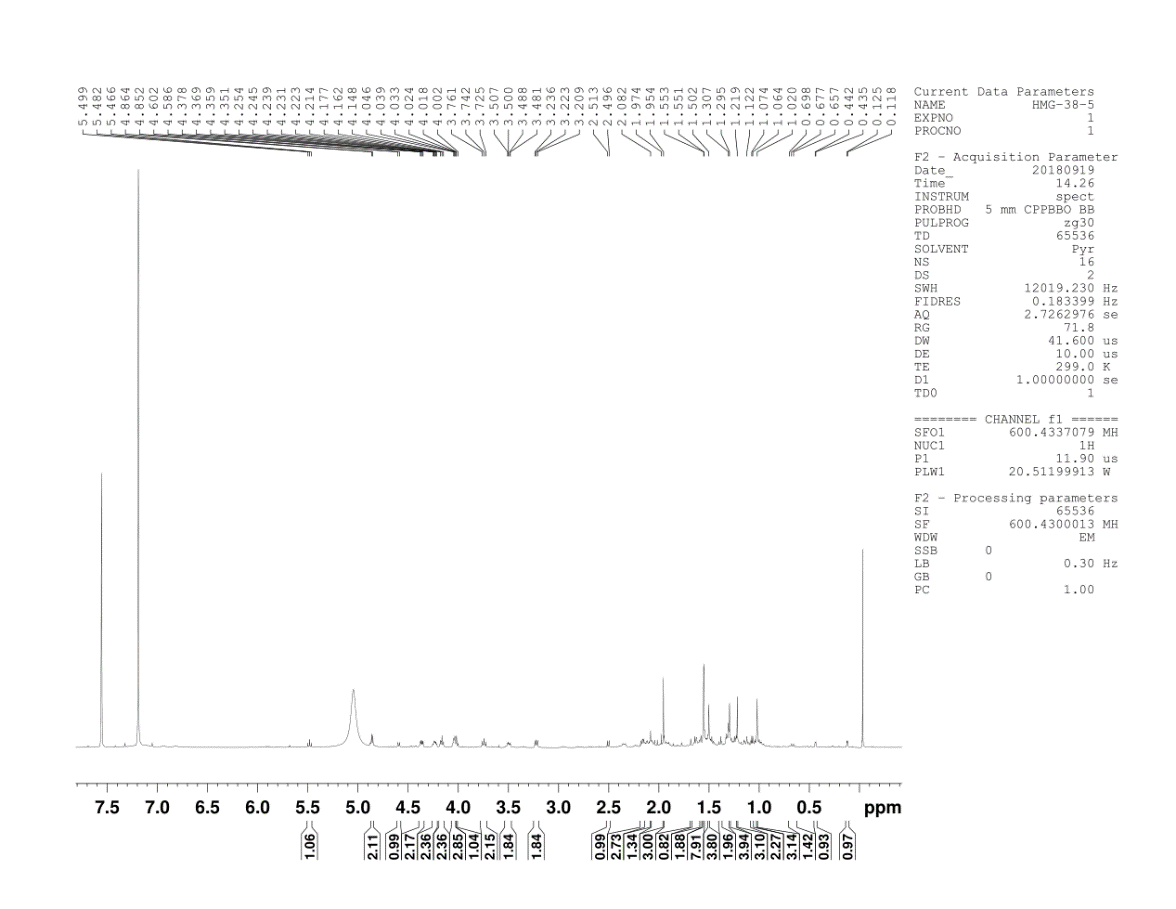


**Figure S46** ^1^H NMR (600 MHz, C5D5N) spectrum of **6**


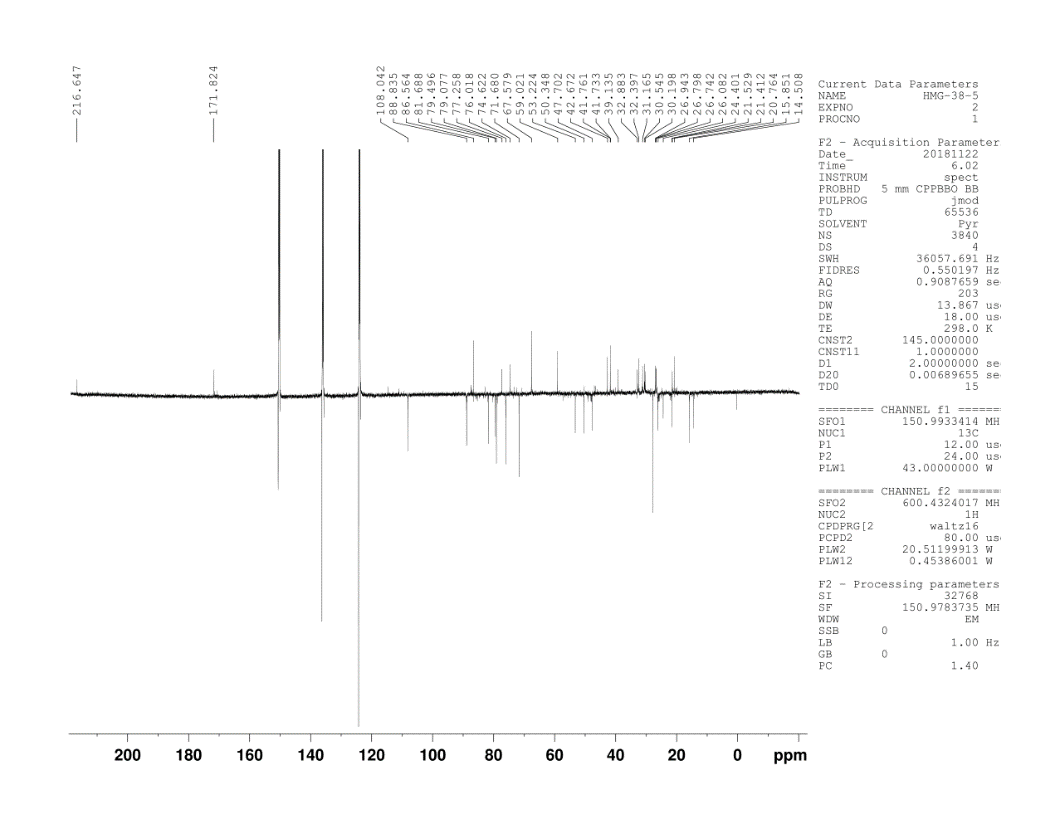


**Figure S47** ^13^C-APT (150 MHz, C5D5N) spectrum of **6**


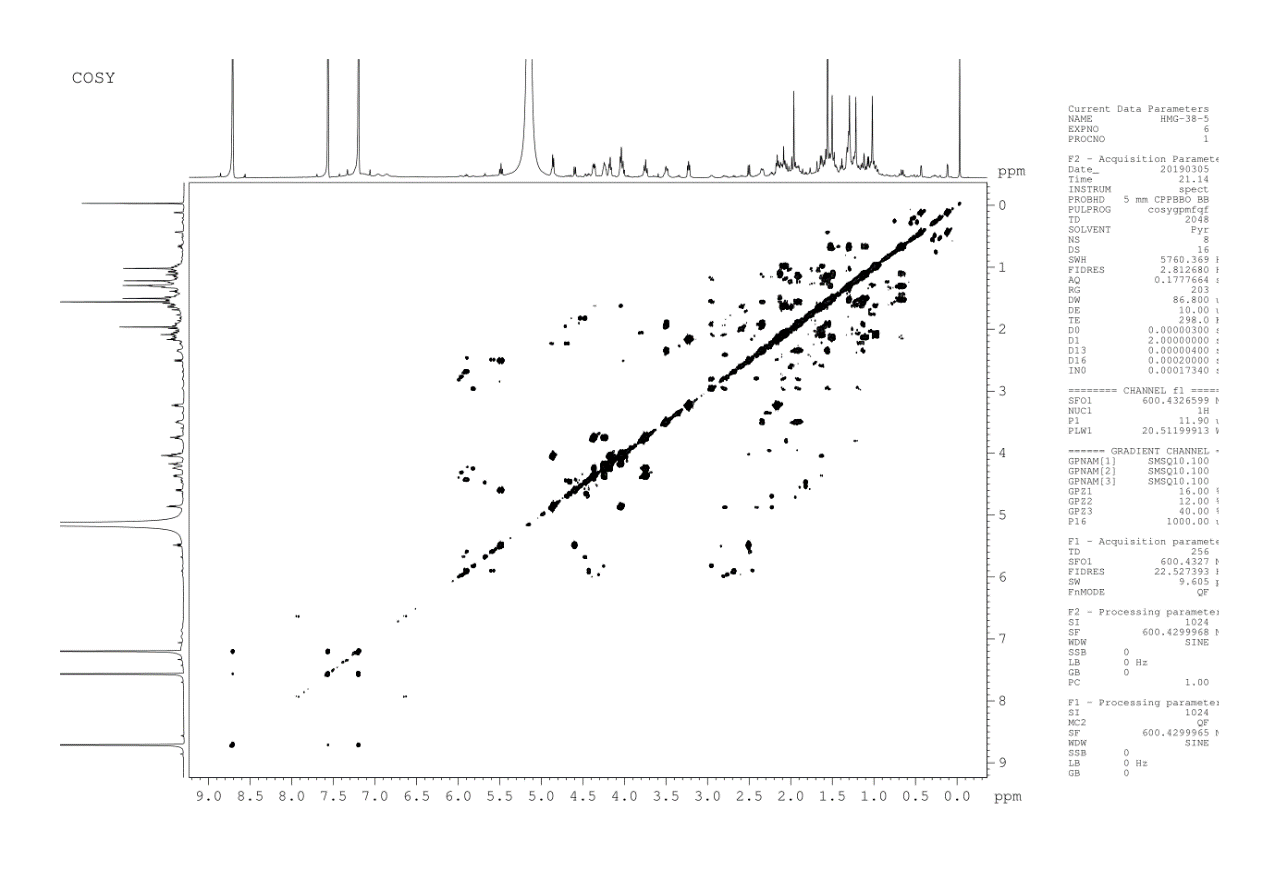


**Figure S48** ^1^H- ^1^H COSY (C5D5N) spectrum of **6**


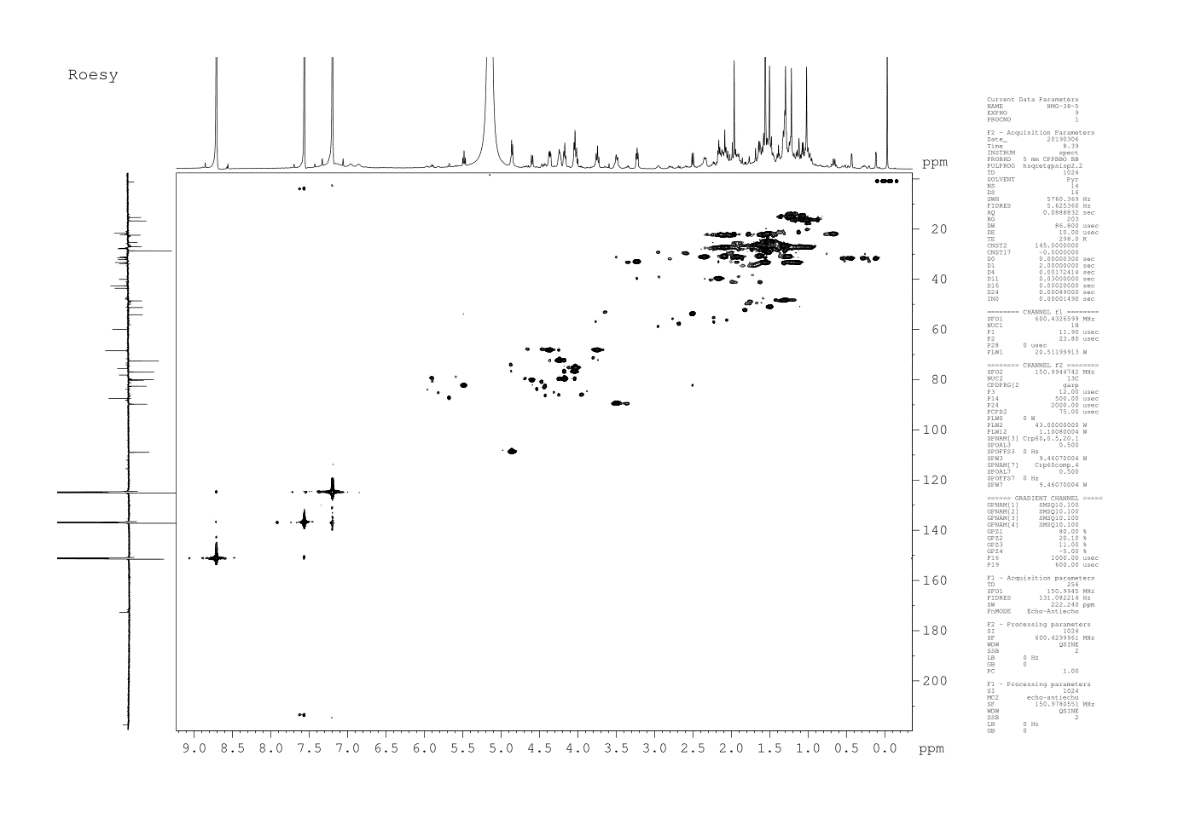


**Figure S49** HSQC (C5D5N) spectrum of **6**


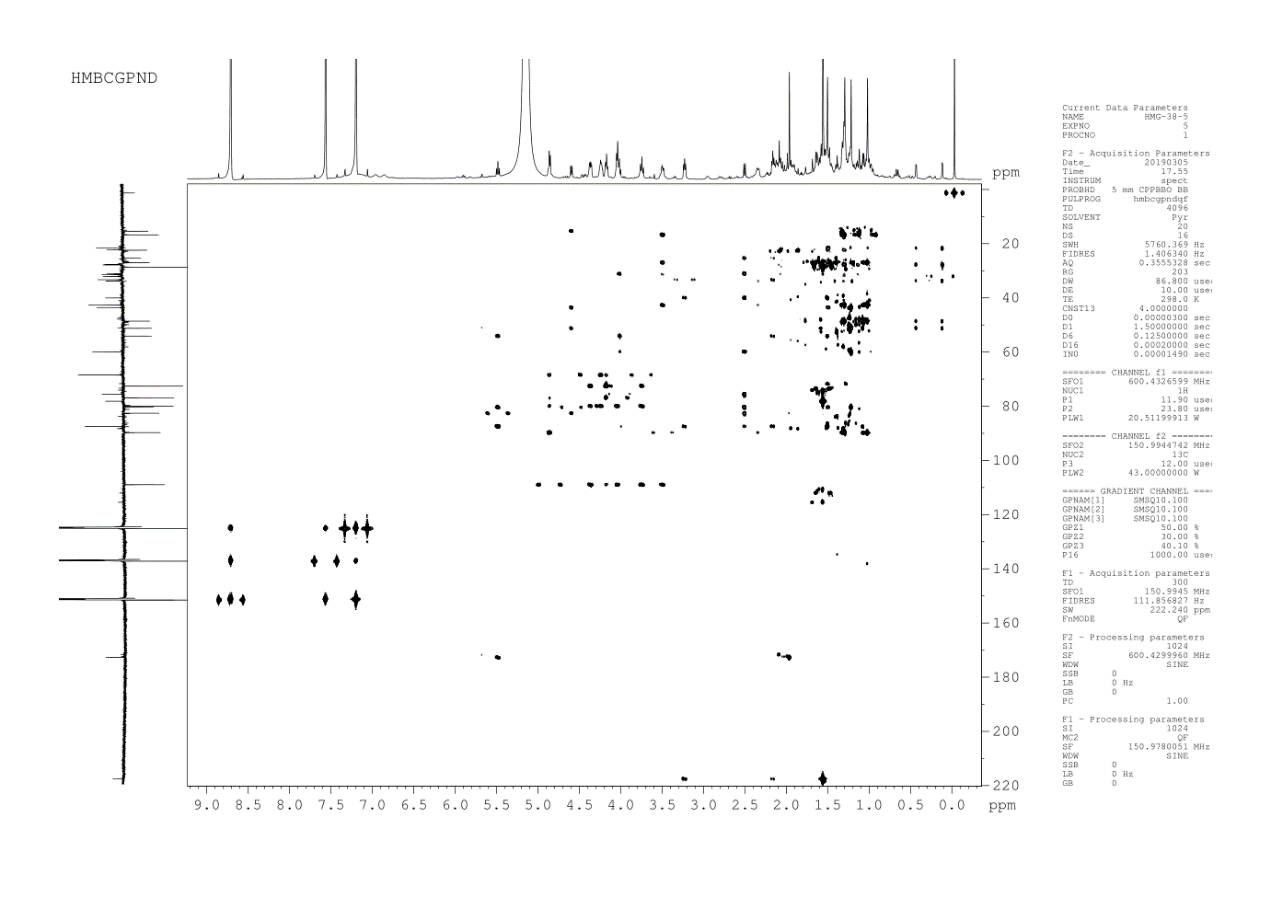


**Figure S50** HMBC (C5D5N) spectrum of **6**


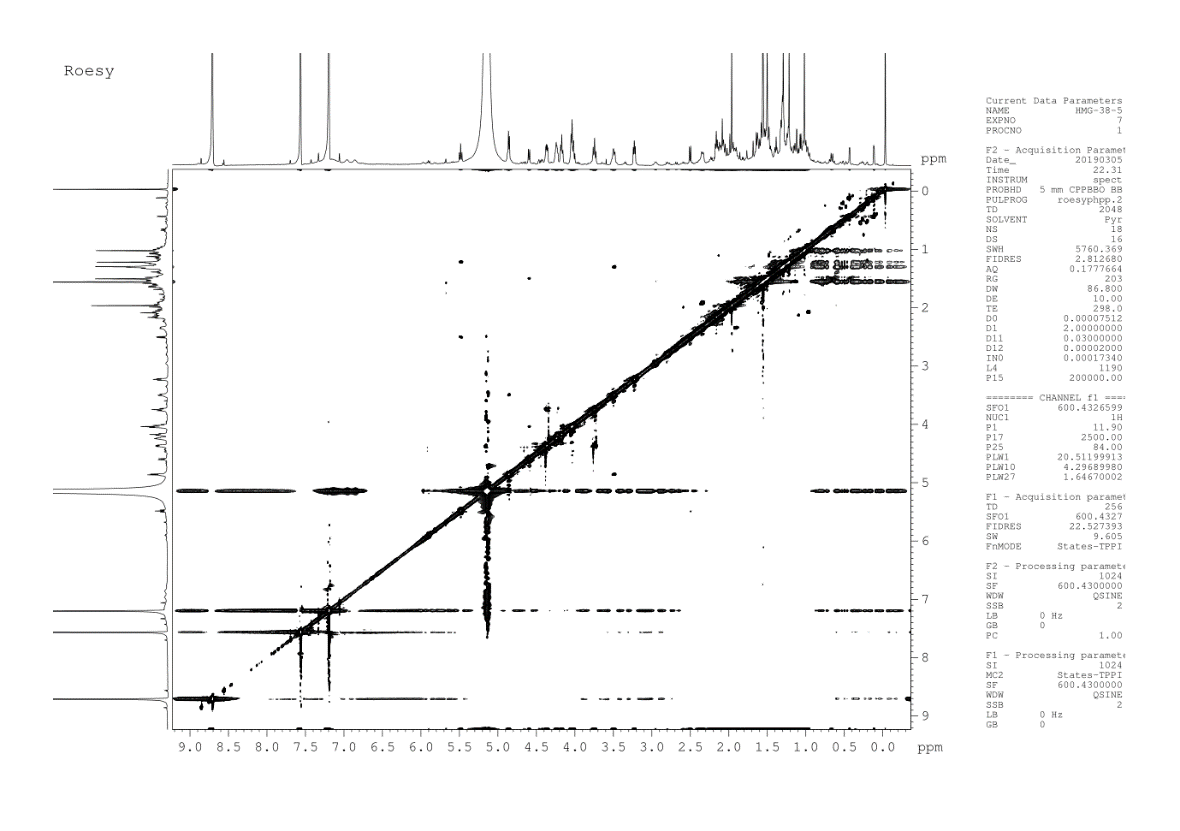


**Figure S51** NOESY (C5D5N) spectrum of **6**


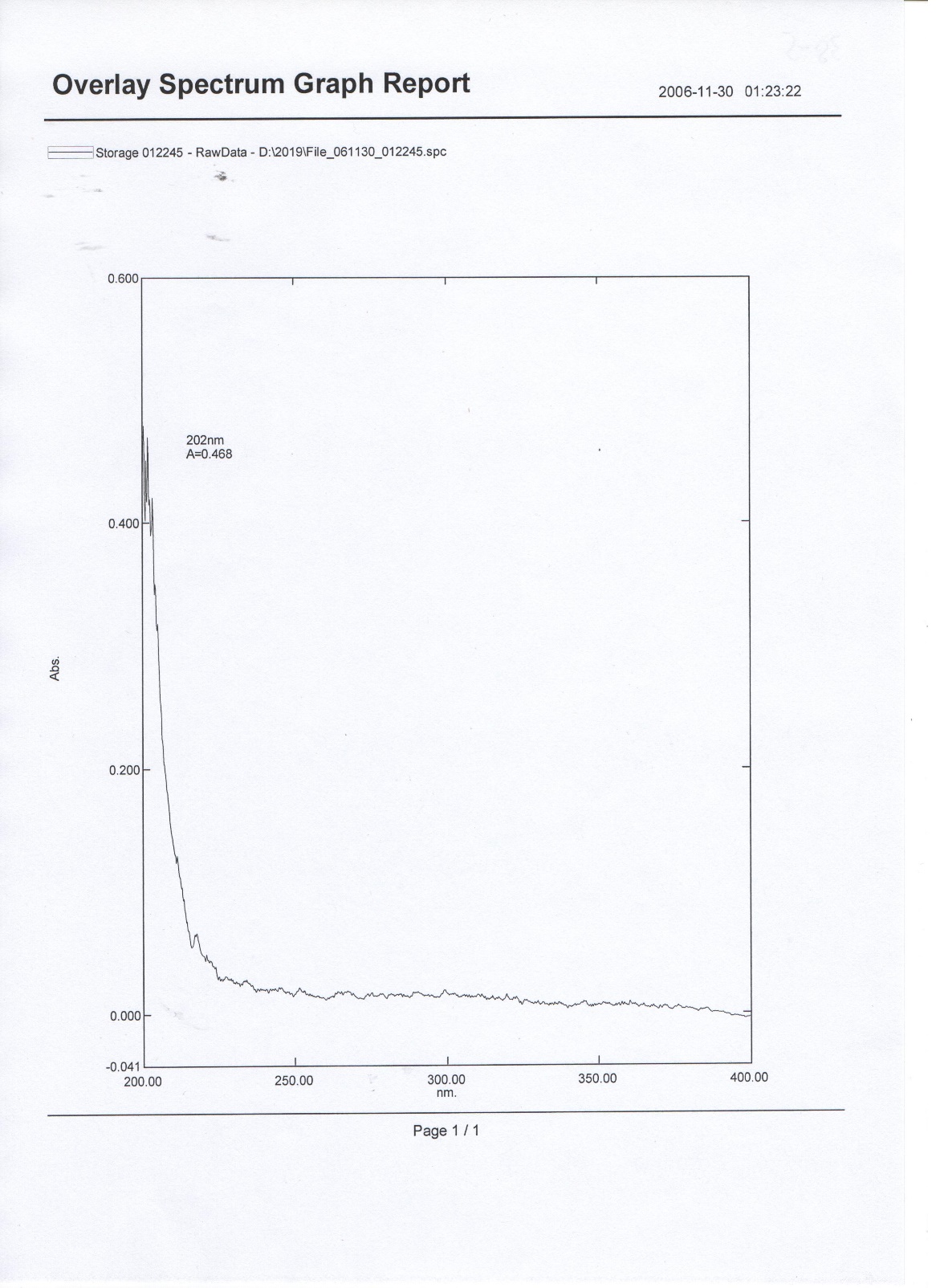


**Figure S52** UV spectrum of **6**


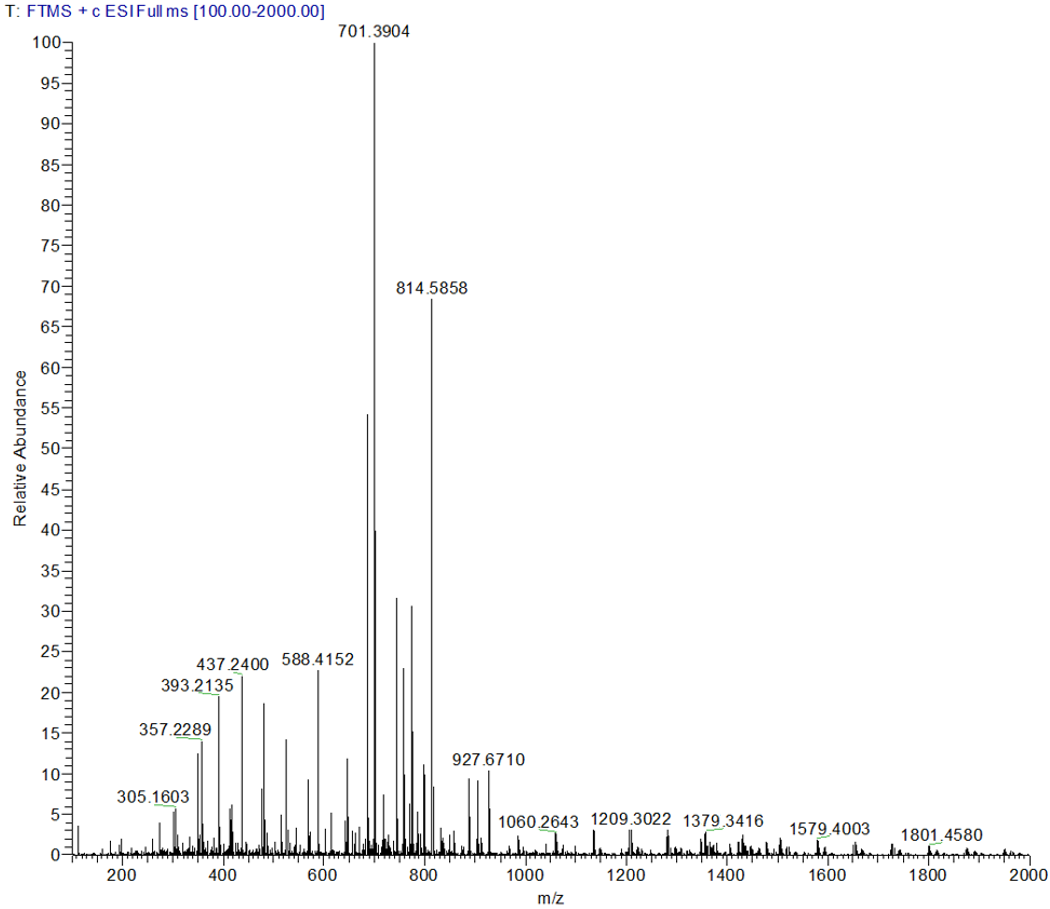


**Figure S53** HRESIMS spectrum of **6**


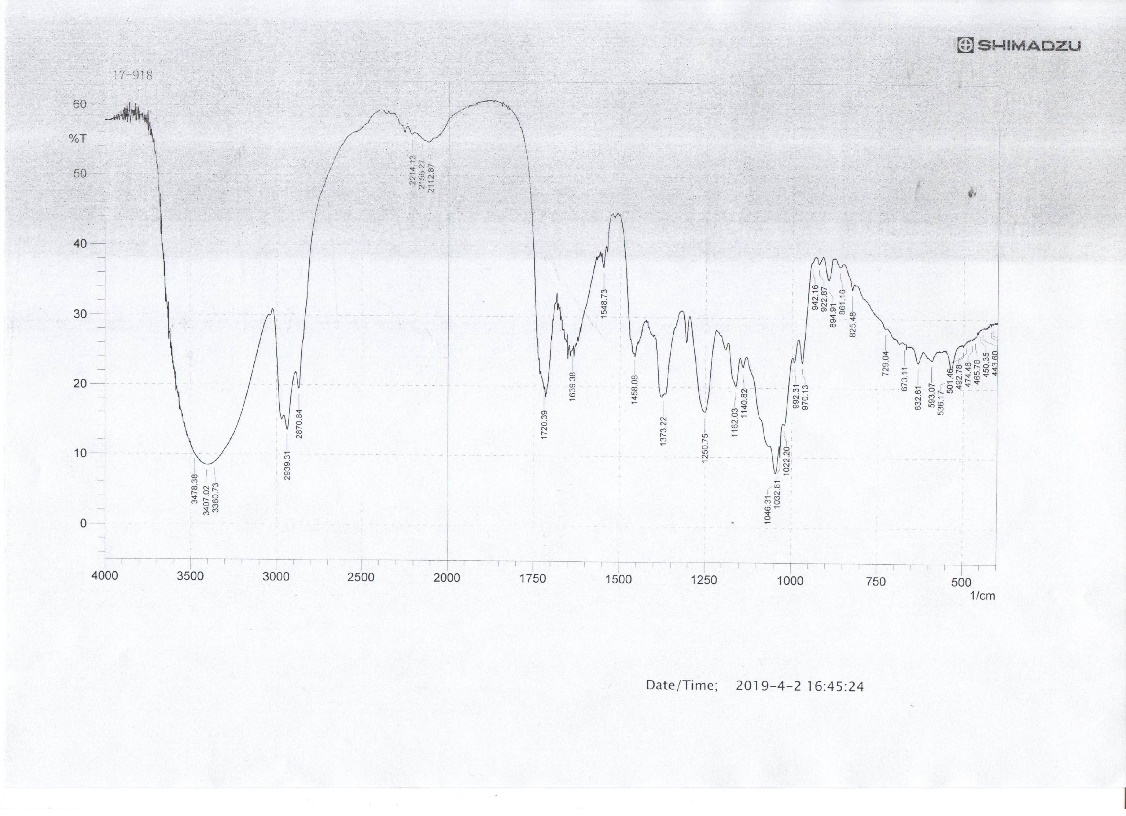


**Figure S54** IR spectrum of **6**


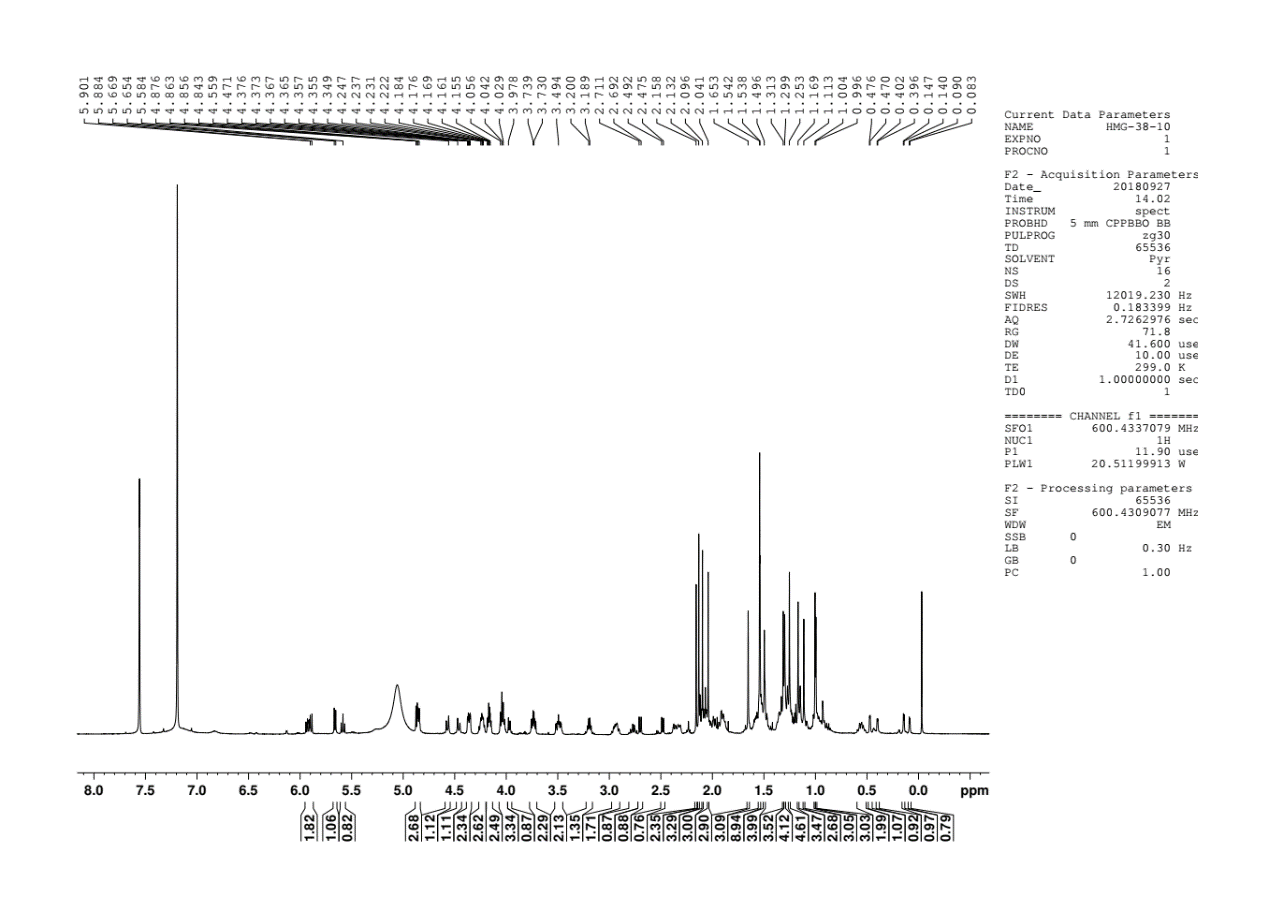


**Figure S55** ^1^H NMR (600 MHz, C5D5N) spectrum of **7**


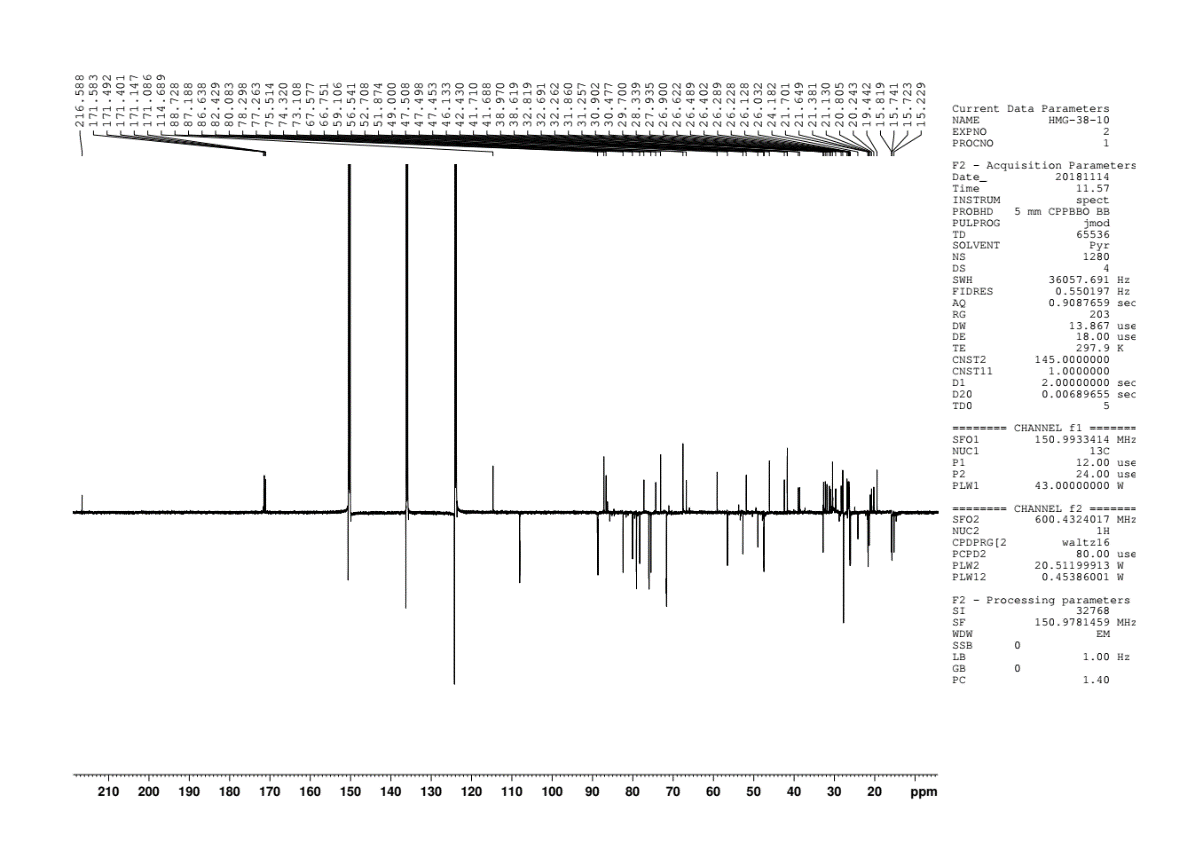


**Figure S56** ^13^C-APT (150 MHz, C5D5N) spectrum of **7**


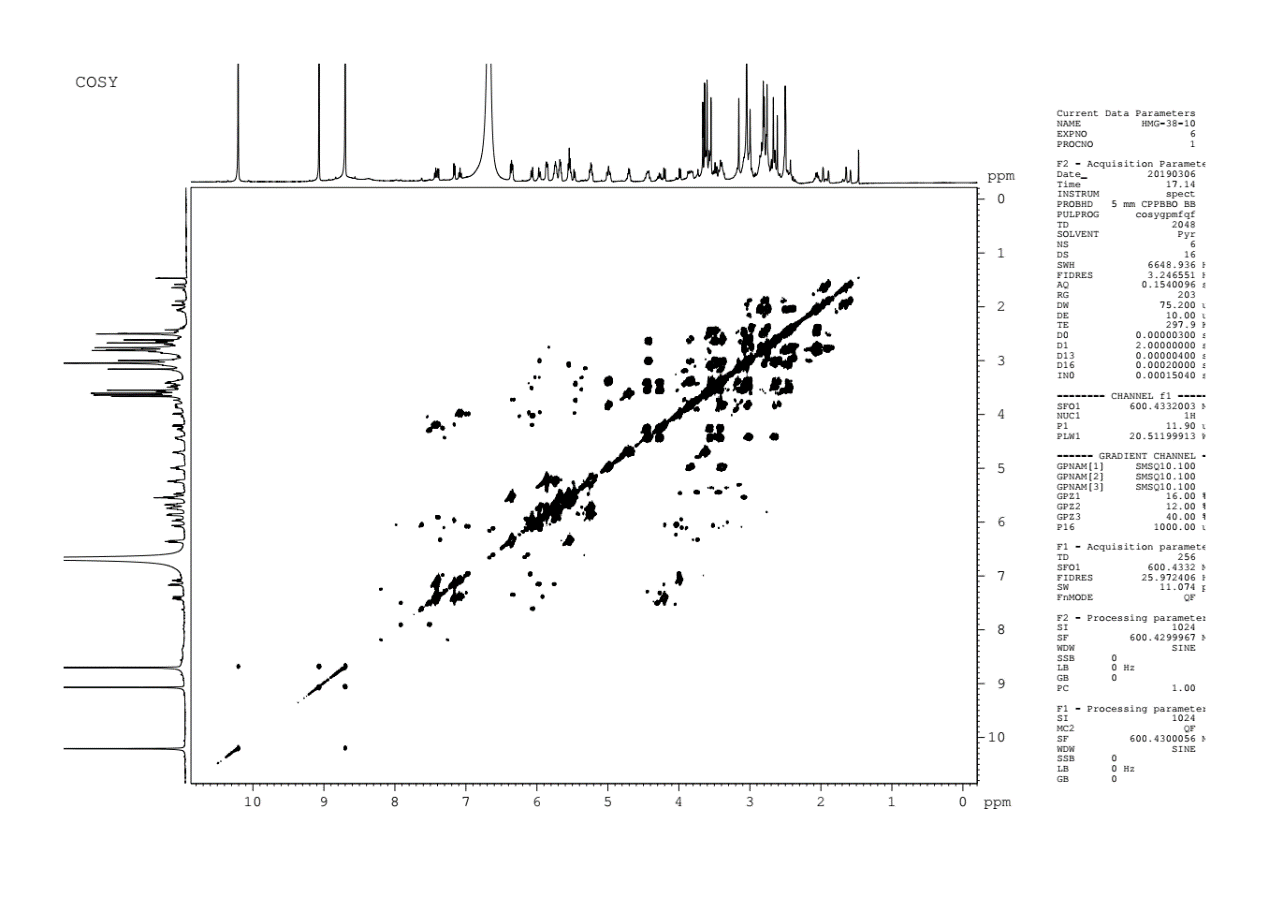


**Figure S57** ^1^H -^1^H COSY (C5D5N) spectrum of **7**


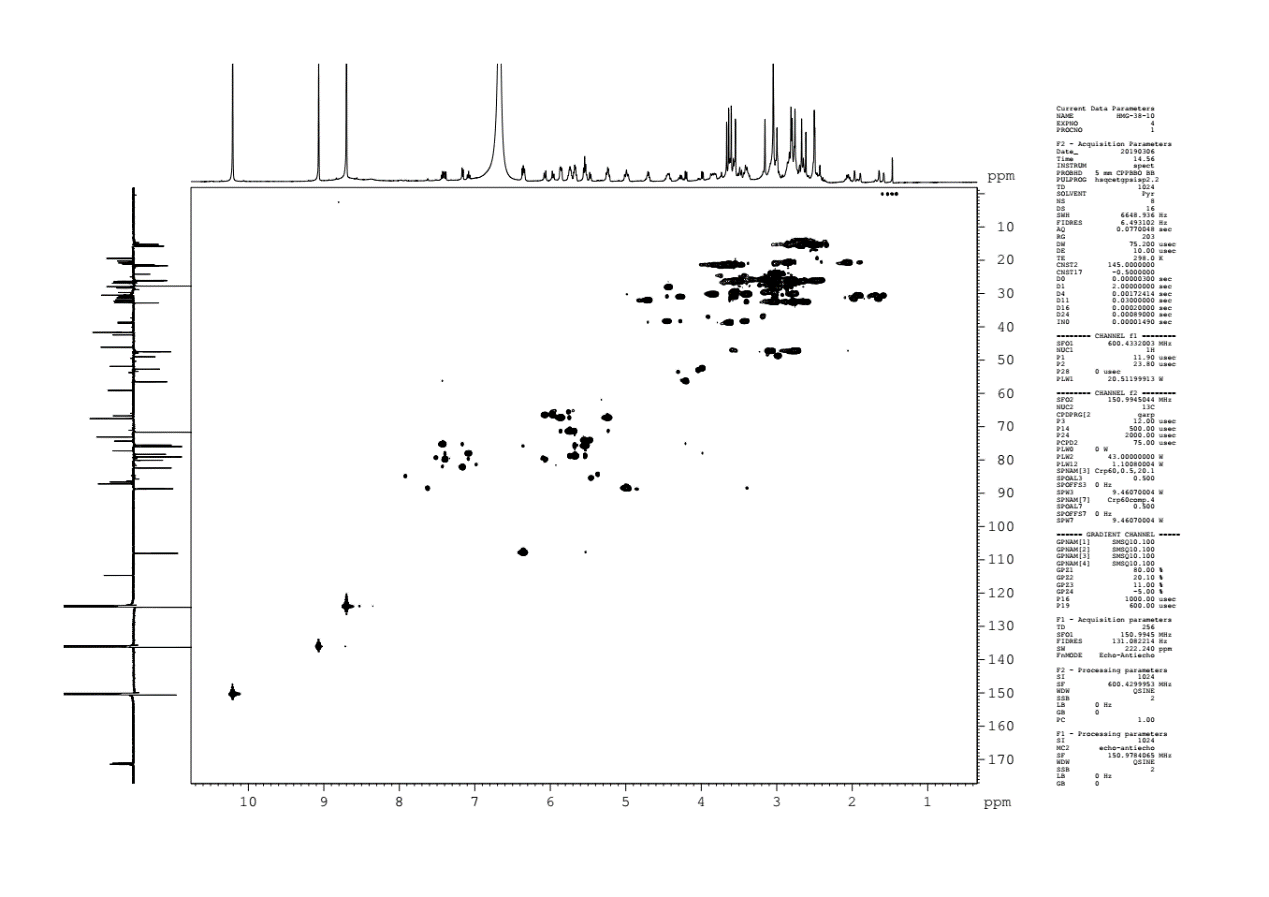


**Figure S58** HSQC (C5D5N) spectrum of **7**


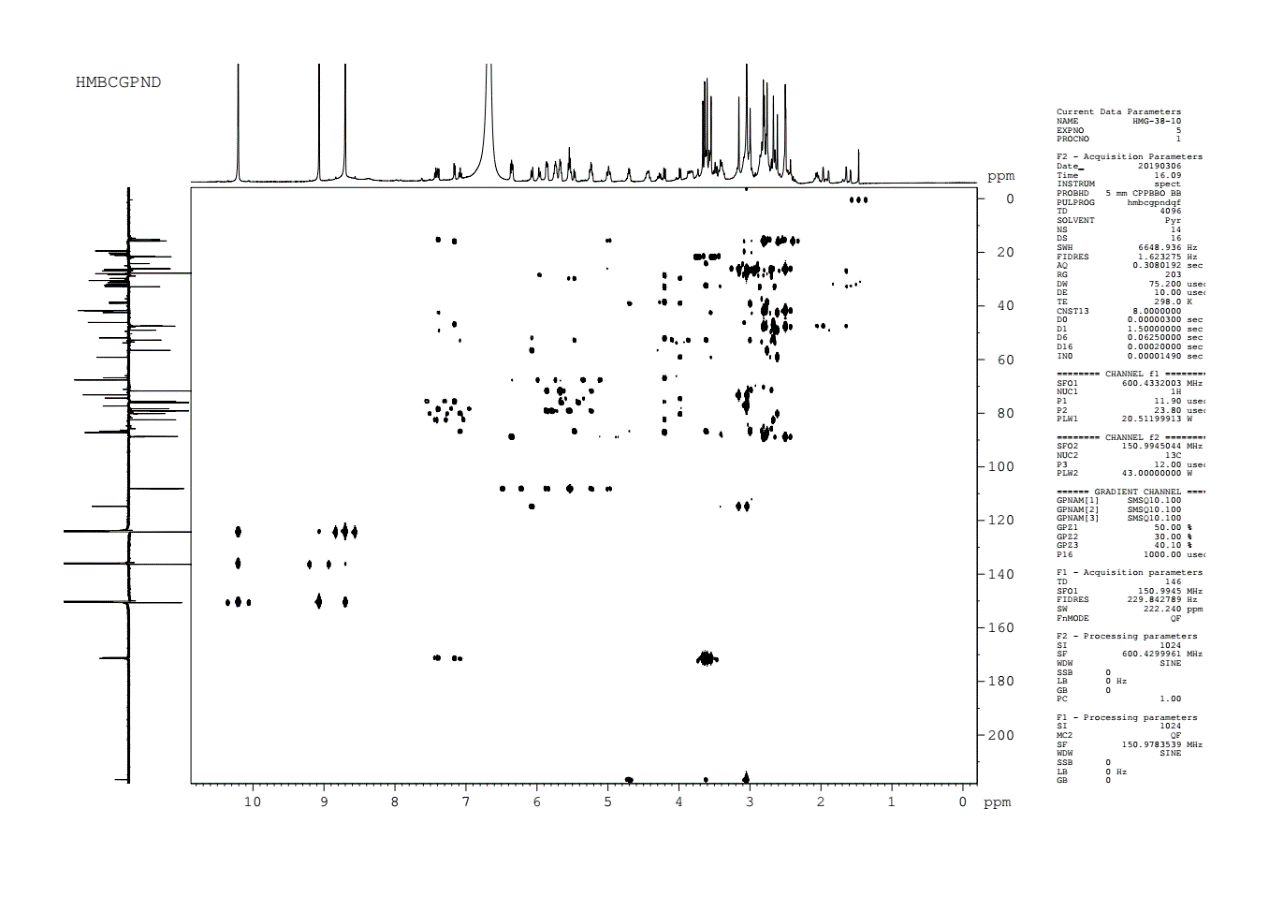


**Figure S59** HMBC (C5D5N) spectrum of **7**


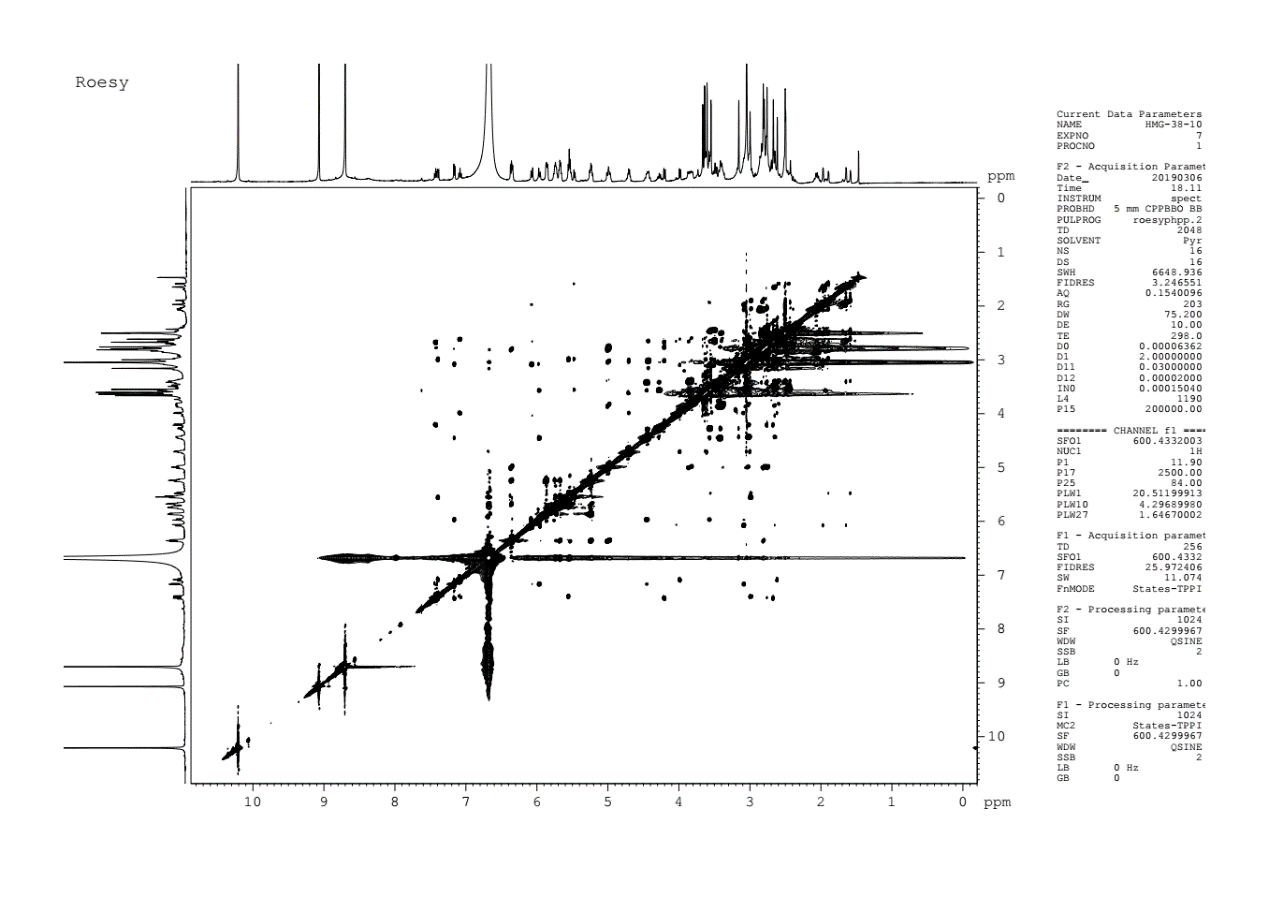


**Figure S60** NOESY (C5D5N) spectrum of **7**


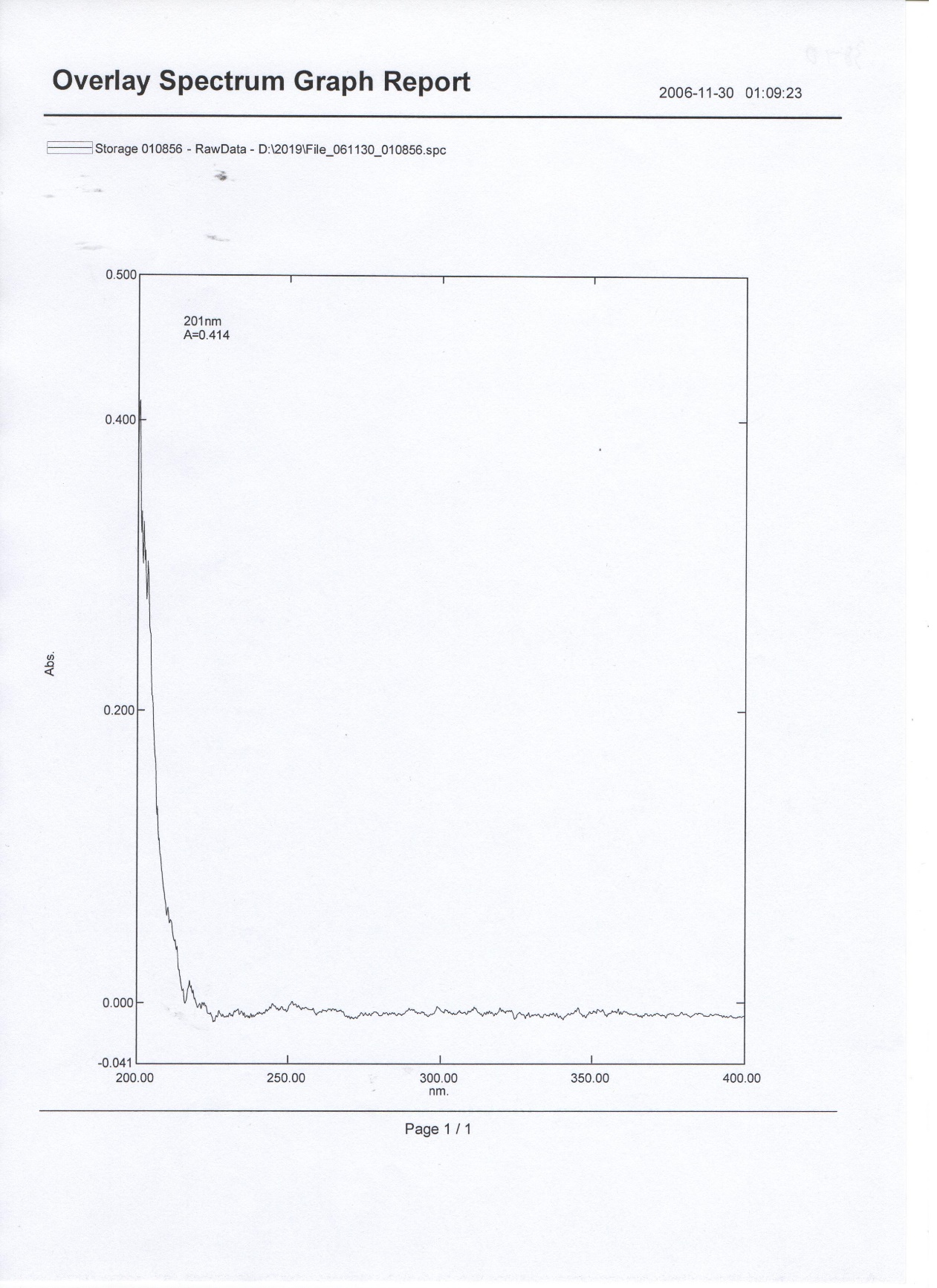


**Figure S61** UV spectrum of **7**


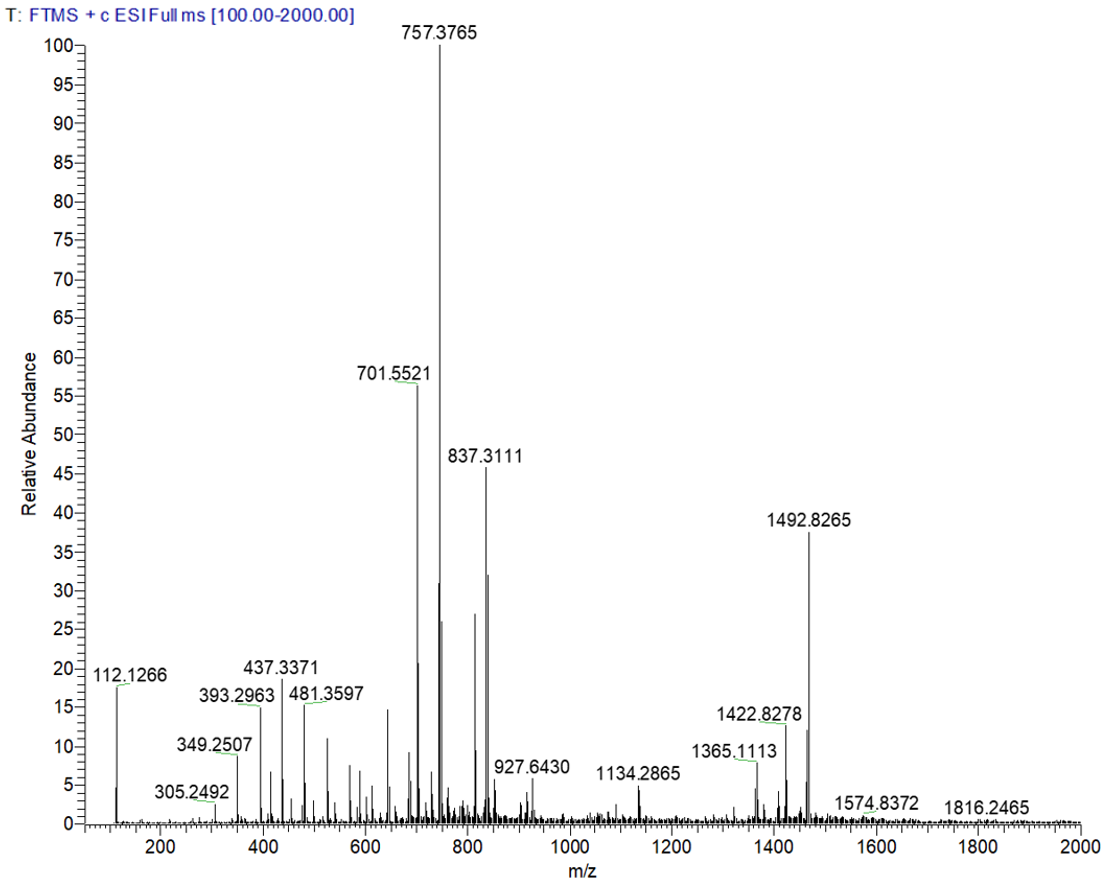


**Figure S55** HRESIMS spectrum of **7**


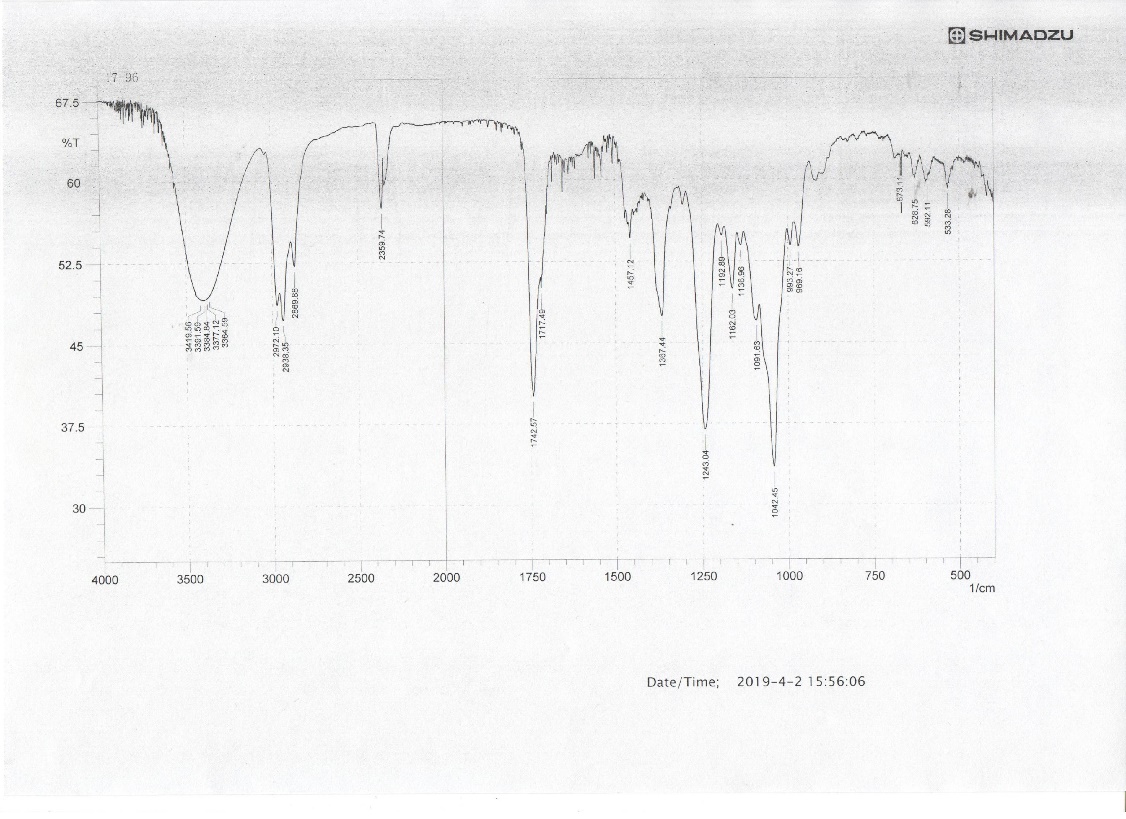


**Figure S56** IR spectrum of **7**
